# Photolytic Hydrophosphination: Insights Into Catalyzed and Uncatalyzed Processes

**DOI:** 10.1002/chem.71053

**Published:** 2026-04-27

**Authors:** Emma J. Finfer, Evelyn M. Kempf, Rory Waterman

**Affiliations:** ^1^ Department of Chemistry University of Vermont Burlington Vermont USA

**Keywords:** hydrophosphination, main‐group elements, metal‐free, photolysis, sustainable chemistry

## Abstract

In the course of studying control reactions for photocatalytic hydrophosphination, several key discoveries have been made, most notably that ambient photoexcitation cannot be ignored in future synthetic work. In this study, catalyst‐free photolytic hydrophosphination is demonstrated for vinyl arenes. These reactions are less efficient than many catalyzed examples, indicating that (photo)catalysis is still highly enabling and necessary for challenging substrates. Nevertheless, these reactions appear to be closed‐shell, affirming that even ambient light can impact a reaction—a potentially broad influence on even mundane reactions. Counterintuitively, polar protic solvents facilitate these reactions under thermal and photolytic conditions. An effort to extend that discovery to more classically facile substrates has revealed Click‐like efficiency in polar protic (alcohol) solvents for activated alkenes, regardless of irradiation. The photochemistry and catalysis help reconcile some divergent observations in the literature. Overall, these results avail new considerations for reactions in which nucleophilic reactivity is enhanced in protic solvent, which is possible due to the absence of a discrete anion. Alcohol solvents may provide greater reactivity than aprotic polar solvents, contrary to conventional wisdom, and even ambient light may be accelerating reactions through unanticipated substrate activation.

## Graphical abstract

Catalyst‐free photolytic hydrophosphination is demonstrated for vinyl arenes and activated alkenes in polar protic (alcohol) solvents. These reactions appear to be closed‐shell, affirming that even ambient light can impact a reaction—a potentially broad influence on even mundane reactions.

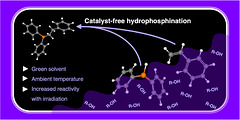

## 1 Introduction

A lynchpin step in the preparation of value‐added reagents and ligands is phosphorus–carbon (P−C) bond formation, but challenges remain [1, 2]. Hydrophosphination is a straightforward approach to P−C bond formation, offering atom economy, and tailored regioselectivity. While significant advances have been made using transition‐metal catalysts and strong bases [3, 4], these approaches can suffer from limitations including synthetically complex catalysts and harsh conditions, diminishing the sustainability of the system and the potential for use in biologically relevant materials [5, 6, 7].

Catalyst‐free hydrophosphination is possible and an attractive alternative, offering not only simplified reaction conditions but also improved environmental compatibility, further consistent with green and sustainable chemistry [8, 9]. Indeed, eliminating catalysts circumvents concerns regarding metal residues in final products, and is particularly beneficial for applications in pharmaceuticals [10, 11, 12, 13], agrochemicals [14, 15, 16, 17], and electronic materials [18, 19, 20]. Furthermore, catalyst‐free systems simplify reaction set‐up and purification, therefore improving scalability and reducing the environmental footprint associated with many catalysts [21].

In prior hydrophosphination catalytic studies, we have found that catalysts were necessary, except for reactions with highly activated alkene substrates, which is consistent with an introductory organic level understanding of these reactions. That trend was upended with studies in ethanol solvent (vide infra). More concerning, though, is that several catalyst‐free studies, thermal or solvent‐dependent, did not afford the same reactivity in our hands, even replicating the conditions to the best of our ability [22, 23, 24]. We have not conducted more detailed study to rationalize those discrepancies. Rather, a deeper consideration of control reactions from photocatalysis has identified even simpler and more green approaches to catalyst‐free hydrophosphination. Furthermore, the observation that catalyst‐free hydrophosphination can be photolytic provides excellent reasons for disparities with prior studies and an important consideration for ambient photoactivation of substrates.

**FIGURE 1:**
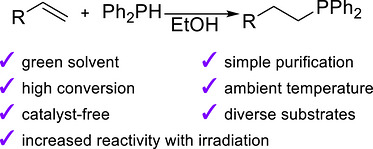
Summary of ambient temperature hydrophosphination in polar protic solvent with the archetypal ethanol shown.

In this study, we report a catalyst‐free hydrophosphination of alkenes in ethanol, providing direct access to P−C bond‐containing products under mild conditions (i.e., ambient temperature, Figure 1). To our best understanding, this approach introduces the mildest conditions to form these secondary and tertiary phosphines. Moreover, these reactions minimize the risk to personnel and environmental footprint associated with classic approaches to hydrophosphination. Catalyst‐free photolytic hydrophosphination for less activated vinyl arenes demonstrates how ambient photolysis may have impacted prior catalyst‐free studies and underscores the continued necessity of catalysis in hydrophosphination.

## 2 Results and Discussion

In a prior study, group I alkoxides and Cu(acac)_2_ were screened in EtOH solution for hydrophosphination [24]. Control reactions in that analysis revealed something intriguing. The catalyst‐free reactions saw increased conversion under both photolytic and ambient conditions. When irradiated at 360 nm for 5 h, a solution of equimolar styrene and diphenylphosphine in ethanol (EtOH) afforded 60% of the anti‐Markovnikov hydrophosphination product. This control reaction was a significant outlier where reactions in other solvents failed to exceed 30% conversion (Table S1) [24]. Yet more interesting, the reaction of diphenylphosphine and ethyl acrylate in EtOH delivered quantitative conversion in merely 10 min under ambient conditions. It is important to note that catalyst‐free hydrophosphination has been reported in 2016 by Alonso and coworkers [23]. Those reactions were conducted neat and primarily heated to 70°C, but in our hands here and as previously reported [24], those conversions could not be replicated for any substrates except activated alkenes. Given that even base metals (e.g. iron) can initiate radical‐induced hydrophosphination and highly active s‐block catalysts are known [2, 5, 25, 26, 27, 28, 29], trace metal contamination of any kind may provide results under forcing conditions. We had not considered ambient irradiation playing a role.

Given the explosive success of s‐block metals in hydrophosphination catalysis, we considered the idea of trace contamination as the culprit for discrepancies between reported acatalytic reactions and our observations. Notably, for both zirconium and calcium photocatalysts, the initial discoveries of photocatalysis were made on the cusp of meaningful absorbance for both metals. A reasonable hypothesis arose: Is the metal needed at all for photolytic hydrophosphination?

### 2.1 Photolytic Hydrophosphination of Vinyl Arenes

**SCHEME 1:**
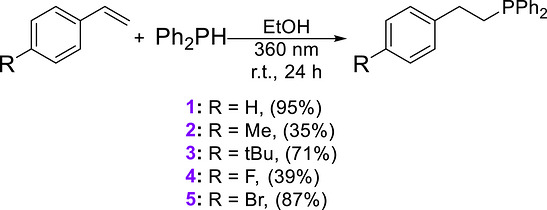
Scope of the reaction of diphenylphosphine with styrene substrates. General reaction conditions: styrene substrate (0.38 mmol), Ph2PH (0.38 mmol), in solvent (0.4 mL). Conversions were the average of at least three trials as determined by 31P NMR spectroscopy.

For catalytic hydrophosphination, styrene has become a benchmark substrate because it differentiates catalysts capable of a conjugate addition mechanism versus those that can access a broader array of substrates. Despite the absence of catalyst, it was a natural place to extend this discovery. It was found that ambient temperature reactions in ethanol were ineffective, and prior instances of elevated temperature reactions showed these to be of limited activity. Reactions were then screened under temperature‐controlled irradiation centered at 360 nm. Thus, equimolar styrene and diphenylphosphine were irradiated with a lamp centered at 360 nm light for 24 h in selected solvent (Scheme 1), which afforded excellent conversion with the caveat that many catalysts are more efficient with this substrate [5, 24, 29, 30]. Solvent effects for this reaction were considered (see Supporting Information), but there was no clear pattern among solvents based on typical solvent parameters like dielectric constant or any Kamlet‐Taft solvent parameters. Ethanol was superior to those screened and continued in these studies.

To gain additional understanding, five *para*‐substituted vinyl arenes were considered under these reaction conditions. Irradiation of equimolar styrene substrate and diphenylphosphine in EtOH was monitored by ^31^P NMR spectroscopy over a 24 h period (Scheme 1). The conversions defied a pattern based on Hammett considerations in which styrene afforded the greatest conversion and there being no correlation of electron donating or withdrawing substituents on conversion. An exploration of solvent demonstrated that alcohols were superior, and organic solvents gave mixed results (Table S3). Germane to prior uncatalyzed reports, alcohol solvents were among the few to exceed the performance of a neat reaction between alkene and diphenylphosphine.

**SCHEME 2:**
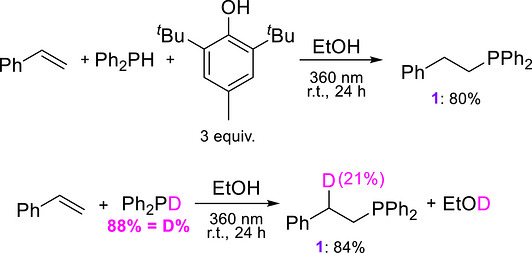
Reactions inconsistent with significant radical behavior. An attempted trapping reaction with excess BHT (above) and selective deuterium incorporation at the a position using diphenylposphnine‐d1 under standard conditions.

Despite the tolerance for styrene, which can be deactivated in radical‐initiated hydrophosphination [25], these observations might imply a radical pathway. However, a silent EPR spectrum of this reaction at ambient temperature under irradiation is inconsistent with that assertion. A reaction run in the presence of three equivalents of BHT gave only slightly reduced conversion (80%), providing no strong indication other than not an outwardly radical‐based reaction (Scheme 2). Likewise, selective deuteration is also inconsistent with a radical process (Scheme 2). Additionally, chloromethylcyclopropane was used as a potential radical clock, added into reactions. The formation of 1‐butene other ring opening products, the expected degradation product in the presence of radicals, was not detected, and only chloromethylcyclopropane was recovered.

**SCHEME 3:**
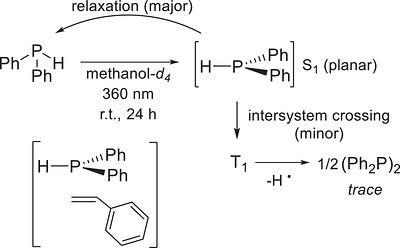
Extended photolysis of diphenylphosphine without alkene substrate in methanol‐d4 over 24 h. Gradual exchange of the phosphine proton is observed with solvent (65% Ph2PD at 24 h, not depicted) and a potentially favorable substrate approach to the planar S1 intermediate (lower left).

The reason for this reactivity is captured in the photophysics of the diphenylphosphine substrate. Analysis of spectra for this reagent have been on‐going for decades, and the spectroscopic similarity between diphenylphosphine and diphenylamine allow for some reasonable mechanistic hypotheses to be made from more detailed spectroscopy and experimentation on the latter [31, 32, 33, 34, 35, 36, 37]. Excitation of these Ph_2_EH compounds results in a planar‐at‐E S_1_ state [32]. These compounds can engage in intersystem crossing to a triplet (T_1_) that is known to decompose by hydrogen atom loss [32], but the photoionization of such compounds is much less in alcohols than polar organics [31]. Therefore, the S_1_ state is either more stable or longer lived, resulting in either relaxation to the ground state without decay or productive hydrophosphination. The T_1_ state remains accessible, and extended irradiation of pure samples of diphenylphosphine in alcohol solvent reveals reliable though trace formation of tetraphenyldiphosphine, the product of hydrogen atom loss (Scheme 3). This radical reactivity is so limited, it escapes detection by EPR spectroscopy under irradiation and must have a noncompetitive rate relative to productive hydrophosphination when a vinyl arene is introduced (vide infra).

The hydrophosphination event is different than reported photocatalyst based on more detailed study of transition‐metal catalysts that form metal‐phosphide intermediates [30, 38, 39, 40]. Indeed, there is no metal‐phosphorus bond to affect insertion at all. The excited state study of Ph_2_EH compound provides a reasonable mechanistic hypothesis for the hydrophosphination reaction. Because the S_1_ state is essentially planar at E, that element is effectively rehybridized to sp^2^. Symmetry prohibitions on the cycloaddition reactions have now changed, and reactivity of the S_1_ with alkene has changed from a conjugate addition. An analogy for such a change in reactivity of the phosphine under these conditions is ruthenium‐catalyzed hydrosilylation reported by Tilley in which a canonical resonance structure becomes dominant to allow a terminal silylene ligand to engage in hydroborylation‐like reactivity [41]. This hypothesis is buttressed by the selectivity for vinyl arene and inactivity toward α‐olefins (vide infra), where the former can engage in an encounter complex with the S_1_ state that is stabilized with π‐π interactions.

**SCHEME 4:**
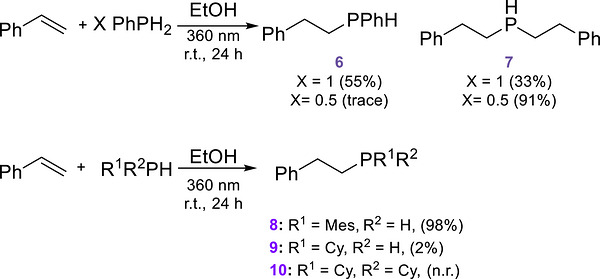
The substrate scope of catalytic hydrophosphination of styrene with unique primary and secondary phosphines in EtOH at ambient temperature. For phenylphosphine, full consumption of styrene was observed in all cases, but a mixture of single and double activation products were observed unless the reaction was run in a 2:1 ratio of substrates to obtain product **7**.

To verify these suppositions, changes in phosphine substrate were explored. The spectroscopy of Ph_2_EH and PhEH_2_ compounds exhibit high similarities [33, 36]. Therefore, phenylphosphine was a natural choice to explore the effectiveness of this photolytic chemistry. Indeed, reaction of equimolar phenylphosphine and styrene in ethanol under irradiation centered at 360 nm resulted in complete consumption of styrene substate to a mixture of single and double P−H bond activation product (Scheme 4). The reaction run with 0.5 equiv. of phenylphosphine generated almost exclusively the double P−H bond activation product **7**. This heightened reactivity was entirely consistent with the proposed photolytic hypothesis because phenylphosphine has a lower absorbance energy that is closer to the emission energy of the light source, despite the slightly greater ε values for the secondary phosphine [34]. Additionally, the increase in possible π‐π interactions of the single P−H bond activation intermediate **6** may facilitate the second addition reaction, explaining the facile conversion to the double P−H bond activation product. Mesitylphosphine, with its greater steric profile, afforded exclusively the single P−H bond activation product under irradiation with equimolar styrene, consistent with the electronics of the proposed mechanism but steric factors now play a role in selectivity.

Cyclohexyl‐substituted phosphines provided key negative results that affirm this working hypothesis. Dicyclohexylphosphine afforded no conversion under reaction conditions. This substate access the same planar S_1_ state at higher energy per the UV‐vis spectra, which may allow for unprotective photoexcitation of the unsaturated substrate prior to productive hydrophosphination, but it is also a highly encumbered and challenging substrate [5, 27]. Less bulky cyclohexylphosphine also fails to provide substantive conversion under these conditions, further supporting that the electronic structure of phosphine is a critical component and consistent with prior reports of uncatalyzed hydrophosphination that have been exclusive to diphenylphosphine [22, 23]. An alternative mechanistic hypothesis of spontaneous P–H dissociation has been advanced for uncatalyzed hydrophosphinylation under neat conditions [42]. The significantly greater pK_a_ values for both alkyl phosphines compared to phenyl‐ and diphenylphosphine suggests that ionization is not unreasonable pathway to explain the reactivity difference [43]. However, the substantially divergent relative rates in uncatalyzed reactions for the phosphorus(V) compounds that have more similar pK_a_ values to those of arylphosphines is difficult to rationalize with P–H ionization, especially given the pK_a_ of ethanol.

From the data in these experiments, prior results, and the spectroscopic study of these substrates, it is clear that the photolytic reaction in the absence of a catalyst is substantially different from the photo*catalytic* reactions [30, 38, 39, 40]. Nevertheless, there is significant conversion under these conditions, better than many reported catalysts [5]. These observations indicate that ambient photolysis is a nontrivial consideration, especially because commonplace fluorescence lighting invariably includes some UV emission [44]. The degree of ambient photolysis may also provide a basis for reconciling the disparities between reports of spontaneous hydrophosphination reactions and repeated instances of little to no reactivity for control reactions as part of catalytic studies.

### 2.2 Activated Alkenes

In contrast, activated alkenes are anticipated to proceed catalyst‐free in hydrophosphination, but the control reactions noted substantially exceeded other examples at ambient temperature [22]. The potential for highly efficient catalyst‐free hydrophosphination at ambient temperature, which is unprecedented, demanded further exploration. In this analysis, solvent choice plays a critical and clear role in determining the relative reactivity for the hydrophosphination of these Michael acceptors and assists in elevating the sustainability of the transformation [45]. Ethanol is a safe and scalable reaction medium due to its low toxicity, renewability, biodegradability, and widespread availability [46]. Compared to commonly employed aprotic organic solvents, ethanol is significantly greener, while retaining access to a wide range of functional groups [47].

**FIGURE 2:**
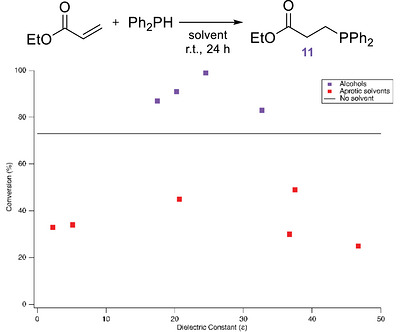
Plot of conversion versus dielectric constant for solvents screened in the hydrophosphination of ethyl acrylate with diphenylphosphine at ambient temperature. Relatively lengthy 24 h reaction time was used to provide meaningful conversions for aprotic solvents. Solvent‐free (neat) conversion at ambient temperature over the same reaction time is represented as a line. General reaction conditions: Ethyl acrylate (0.38 mmol), Ph_2_PH (0.38 mmol), in solvent (0.4 mL). Conversion determined by ^31^P NMR spectroscopy.

To ascertain the effect of solvent on hydrophosphination with activated alkenes, three classes of solvents were investigated: polar aprotic, nonpolar, and polar protic. Conversions for a standard reaction were plotted against solvent dielectric constants (Figure 2). In this analysis, equimolar diphenylphosphine and ethyl acrylate were allowed to react in the selected solvent at ambient temperature and progress was monitored by ^31^P and ^1^H NMR spectroscopy over 24 h. This analysis demonstrates that dielectric constant alone is an insufficient predictor of reactivity. Polar aprotic solvents [DMSO (ε = 46.7), DMF (ε = 36.7), acetone (ε = 20.7), and acetonitrile (ε = 37.5) [48] afford only modest conversion (25%–49%) underscoring that increased polarity does not directly correlate to enhanced reactivity, counter to textbook expectations for nucleophilic reactivity. Low polarity aromatic solvents (benzene and bromobenzene) also afford poor conversion of approximately 33% for each. Conversion for every aprotic solvent is less than that of the neat (i.e., solvent‐free) conditions, also at ambient temperature. Even within the wide range of conversion in an absolute sense (∼100% difference between greatest and least), the set of aprotic solvents tested exhibits a surprising consistency of poor conversion *despite* the relative polarity differences within the set sampled.

Alcohols, in contrast, demonstrate pronounced enhancement in reactivity among a sample of normal alcohols (MeOH, EtOH, *^n^* PrOH, and *^n^* BuOH) that all achieve conversions greater than the neat baseline (Figure 2). In that set, ethanol afforded the greatest conversion. In summary, not only does dielectric constant alone fail to provide predictive power for conversion, (*cf*. acetone with 45% at ε = 20.7 vs. *^n^* PrOH with 91% at ε = 20.3) but aprotic solvents appear to *inhibit* the reaction as compared to neat or polar protic conditions, regardless of polarity. The benefit of protic solvents may be connected to the final proton transfer after nucleophilic attack because there is not a discrete anion to be stabilized, which indicates why dielectric constant is not a predictor for reactivity. Charge stabilization may be a significant effect. In review, it was suggested that 1,1,1,3,3,3‐hexafluoro‐2‐propanol (HFP) may provide outsized reactivity if these effects are important. Replicating reactions of each styrene and ethyl acrylate with diphenylphosphine as above afforded two interesting observations. First, the reactivity of styrene in HFP was similar to, though not better than, the family of alcohols screened. Although, the reaction with ethyl acrylate afforded diminished conversion (50%). Second, reaction mixtures did not appear to be fully miscible in HFP solution. Due to the latter observation, it appears this experiment did not fully address that assertion and the question of charge stabilization after attack remains open.

**SCHEME 5:**
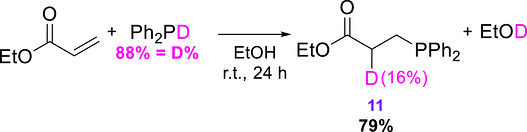
Deuterium labelling reactions with dipheneylphospine‐d1 and ethy lacrylate under standard conditions. Exchange with solvent is highly competitive, explaining the low deuterium incorporation but selectivity is unaffected.

In ethyl acrylate and diphenylphosphine‐*d_1_*, deuterium was added exclusively to the α‐carbon, consistent with a closed‐shell, nucleophilic mechanism (Scheme 5) [30]. More important, the deuterium incorporation was limited in this reaction due to exchange with solvent. However, the degree to which ethanol is deuterated in this reaction is greater than the control photolysis reaction of diphenylphosphine in methanol‐*d*_4_. This observation is parallels the higher relative reactivity of hydrophosphination tun in ethanol versus methanol (Figure 2) and the notion that alcohol may be playing an important role in proton transfer as a rationale for greater reactivity of alcohols in general.

**SCHEME 6:**
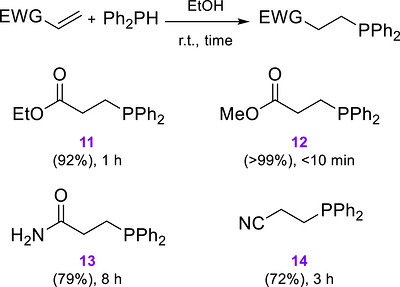
Products of ethanol‐mediated hydrophosphination of Michael acceptors.

Related activated alkenes, including other α,β unsaturated alkenes, were screened in ethanol as the optimal solvent. The unsaturated substrate was treated with one equiv. of diphenylphosphine in an EtOH solution and allowed to react at ambient temperature for no more than 3 h with periodic monitoring by ^31^P NMR spectroscopy. These conditions afforded the respective tertiary phosphine in good yields in all cases, with minimal energy input (Scheme 6). Nitrogen‐containing Michael acceptors (acrylamide and acrylonitrile) demonstrated the more lethargic relative rates of reaction when compared with nonnitrogen containing alkenes (Scheme 6). Methyl acrylate afforded rapid conversion to the corresponding tertiary phosphine **12** while ethyl acrylate afforded 92% conversion after only 1 h to **11**. The consistent conversions for activated alkenes elevates this transformation, meeting the criteria for a click reaction, like hydrothiolation [49].

**SCHEME 7:**
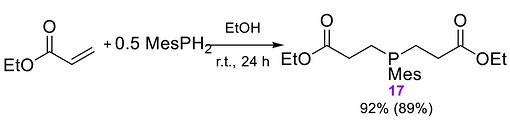
The double hydrophosphination of ethyl acrylate with mesitylphosphine. The value in parenthesis denotes isolated yield.

There are limits to the simple thermal reactivity in ethanol, but these can be surmounted. More substituted activated alkenes suffer from dramatically reduced conversion at ambient temperature. Given the transformative success of photocatalysis [24, 30, 39, 50], reactions of disubstituted activated alkenes were conducted under irradiation. Methyl methacrylate and cyclohexanone were each treated with diphenylphosphine in EtOH and irradiated with a lamp centered at 360 nm for 3 h (Scheme 7). Under these conditions, methyl methacrylate was subject to nearly quantitative conversion to **15**, while cyclohexanone afforded 56% of the tertiary phosphine product **16**. This discrepancy is likely attributed to the steric hinderance of the cyclohexanone. Under ambient light, methyl methacrylate only afforded 8% conversion in 3 h. Furthermore, gentle heating at 40°C gave only 11% conversion to **15** after 3 h. The ability to solicit greater reactivity despite steric limitations emboldened us to consider more challenging substrates.

**SCHEME 8:**
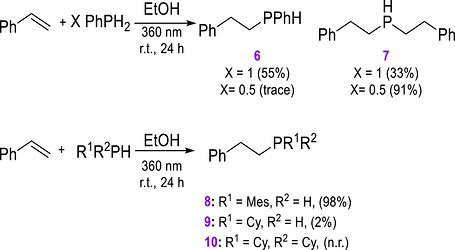
Products of ethanol mediated hydrophosphination of substituted Michael acceptors.

Mesitylphosphine afforded effectively quantitative conversion to product **17** (Scheme 8). Given the observed double P−H bond activation with styrene, a deliberate effort to solicit double P−H bond activation was attempted with mesitylphosphine. Ethyl acrylate was added to 0.5 equiv. of mesitylphosphine and allowed to react for 24 h, whereupon 92% conversion was measured by ^31^P NMR spectroscopy with subsequent isolation in 89% yield. Facile removal of EtOH and MesPH_2_ from the reaction mixture under reduced pressure with no further purification demonstrates the simplicity of this system for synthetic protocols.

## 3 Conclusion

Catalyst‐free hydrophosphination of alkenes is enabled by protic polar solvents, with ethanol being not only optimal in performance but also in convenience and sustainability. Across a wide variety of alkenes, ethanol consistently promotes enhanced reactivity relative to aprotic and nonpolar solvents and even other alcohols. For simple activated alkene substrates, hydrophosphination meets the definition of a Click reaction, demonstrating simplicity, high yield, and lack of complex purification [51]. When substrates become more challenging, catalyst‐free irradiation further enhances reactivity for less activated and more sterically encumbered alkenes. That result helps to reconcile differences in prior catalyst‐free reports and control reactions where many reports typically do not account for ambient illumination. Overall, substrate compatibility, including diverse alkenes and phosphines, emphasizes the generality of the approach. These results expand green hydrophosphination strategies and provide a straightforward platform for the synthesis of organophosphorus compounds.

### 3.1 Experimental Information

General procedure for catalyst‐free hydrophosphination reactions Alkene (0.38 mmol) was added to vial charged with diphenylphosphine (66 µL, 0.38 mmol) and solvent (0.4 mL). The solution was briefly mixed and then transferred to an NMR tube for NMR measurement. Where applicable, the NMR tube was then subjected to 360 nm centered irradiation. Note: Both the setup and an emission spectra for the 360 nm centered lamp have been previously published [30].

## Conflicts of Interest

The authors declare no conflicts of interest.

## Supporting information



The authors have cited additional references within the Supporting Information [52]. Supplementary information (SI): Experimental details and spectroscopic characterization of catalysis runs and products. Original data is available from the corresponding author upon request. See DOI: XXX
