# Supplementary material for: Photolytic Hydrophosphination: Insights Into Catalyzed and Uncatalyzed Processes
Source: Chemistry. 2026 Apr 27;32(31):e71053. doi: 10.1002/chem.71053 (PMC13485185; doi:10.1002/chem.71053)
Supplement: Supplementary file 1 [file CHEM-32-e71053-s001.pdf]

Supporting Information

for

Photolytic Hydrophosphination: Insights into Catalyzed and Uncatalyzed Processes

Emma J. Finfer, Evelyn M. Kempf, and Rory Waterman*

Department of Chemistry, University of Vermont, Burlington, VT 05405, USA
*E-mail : rory.waterman@uvm.edu

Table of Contents

General Information 2
Experimental Details 3
- General procedure for catalyst free reactions 3
- Control reactions from previous study[3] 3
- Hydrophosphination of ethyl acrylate with diphenylphosphine in various solvents 3
  - Solvent effects on the hydrophosphination of ethylacrylate and diphenylphosphine in various solvents 4
- Hydrophosphination of styrene with diphenylphosphine in various solvents 6
  - Solvent effects on the hydrophosphination of styrene and diphenylphosphine in various solvents 7
- Deuterium Labelling Experiments 10
  - Synthesis of diphenylphosphine-d_1 10
  - Deuterium labelling with diphenylphosphine-d_1 10
  - In situ formation of diphenylphosphine-d_1 15
- UV/Visible spectra of Ph_2PH in EtOH 15

NMR spectra for catalyst-free reactions 16
- Hydrophosphination of ethyl acrylate in various solvents 16
- Hydrophosphination of styrene in various solvents 27
- EtOH-mediated hydrophosphination of alkenes 38

References 53

General Information

All the reactions were carried out under a purified N_2 atmosphere using standard Schlenk or glovebox techniques. Diphenylphosphine was prepared by the following literature report.[1] EtOH was stirred over Na and subsequently distilled, and stored over activated 3 Å molecular sieves. All other solvents were dried according to literature procedures. Styrene and its derivatives were treated with three freeze-pump-thaw cycles, distilled, and stored under N_2. M/ethyl acrylate, acrylonitrile, and any other liquid substrates used for hydrophosphination were vacuum distilled over CaH_2 or sodium metal. All alkenes were stored at –30 °C after purification. ^{1}H, ^{2}H, and $^{31}P\{^{1}H\}$ NMR spectra (^{1}H, 500 MHz; ^{2}H, 77 MHz; ^{31}P, 202 MHz) were recorded at 298 K on a Bruker AXR 500 MHz spectrometer. Spectra were reported in δ (ppm) and referenced to $SiMe_4$ (δ = 0.00) for ^{1}H NMR spectra or H_3PH_4 (δ = 0 ppm) for $^{31}P\{^{1}H\}$ NMR spectra. All ^{1}H and $^{31}P\{^{1}H\}$ spectra of hydrophosphination products are consistent with those previously reported in the literature.

Experimental Details

General procedure for catalyst free reactions

Alkene (0.38 mmol) was added to vial charged with diphenylphosphine (66 μL, 0.38 mmol) was and solvent (0.4 mL). The solution was briefly mixed and then transferred to an NMR tube for NMR measurement. Where applicable, the NMR tube was then subjected to 360nm-centered irradiation.

Note: Both a photo of the setup used and an emission spectra for the 360nm-centered lamp have been previously published.[2]

Control reactions from previous study[3]

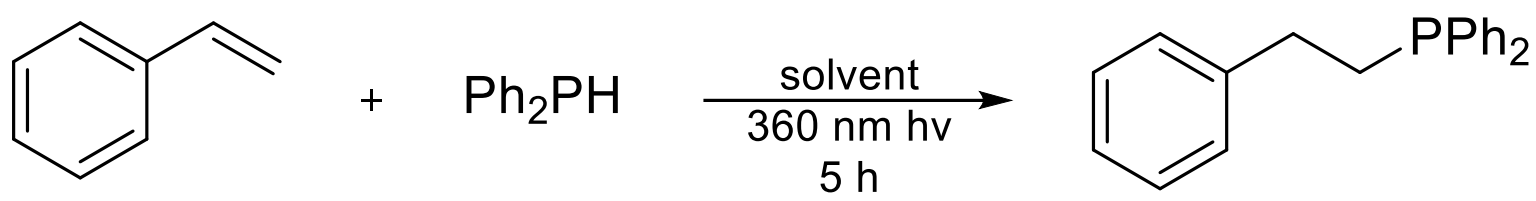

solvent	dielectric constant (ε)	conversion (%)
EtOH	24.5	60
EtOAc	6.02	33
2-MeTHF	6.97	26
cyrene	3.4	23
CPME	4.76	32

Reaction conditions: Styrene (0.38 mmol), diphenylphosphine (0.38 mmol), and solvent were irradiated under 360 nm-centered light for 5h in a PTFE capped NMR tube. Conversions were determine by 31P NMR spectroscopy.

Table S 1. Control reactions of styrene and diphenylphosphine in various solvents from Green Chem. 2025, 27 (2), 432-437.

Hydrophosphination of ethyl acrylate with diphenylphosphine in various solvents

The tabulated values from **Figure** 1 in the main text are described below:

Entry	Solvent	Dielectric Constant (ε)	Conv. (%)
1	Acetone-d_6	20.7	45
2	neat	0	73
3	DMF	36.7	30
4	MeCN	37.5	49
5	C_6H_6	2.27	33

6	C_6H_5Br	5.17	34
7	DMSO-d_6	46.7	25
8	n-BuOH	17.5	87
9	n-PrOH	20.3	91
10	EtOH	24.5	99
11	MeOH	32.7	83

Reaction condition: diphenylphosphine (0.38 mmol), ethyl acrylate (0.38 mmol), solvent (0.4 mL). Conversion was determined by ^{31}P NMR spectroscopy.

Table S 2. Tabulated values of the hydrophosphination of ethyl acrylate with diphenylphosphine in the selected solvents after 24 h at ambient temperature.

<u>Solvent effects on the hydrophosphination of ethylacrylate and diphenylphosphine in various solvents</u>

Key:

Methanol
Acetonitrile
Acetone
Ethanol
DMF
DMSO

*Neat reaction is denoted by flat, solid line.

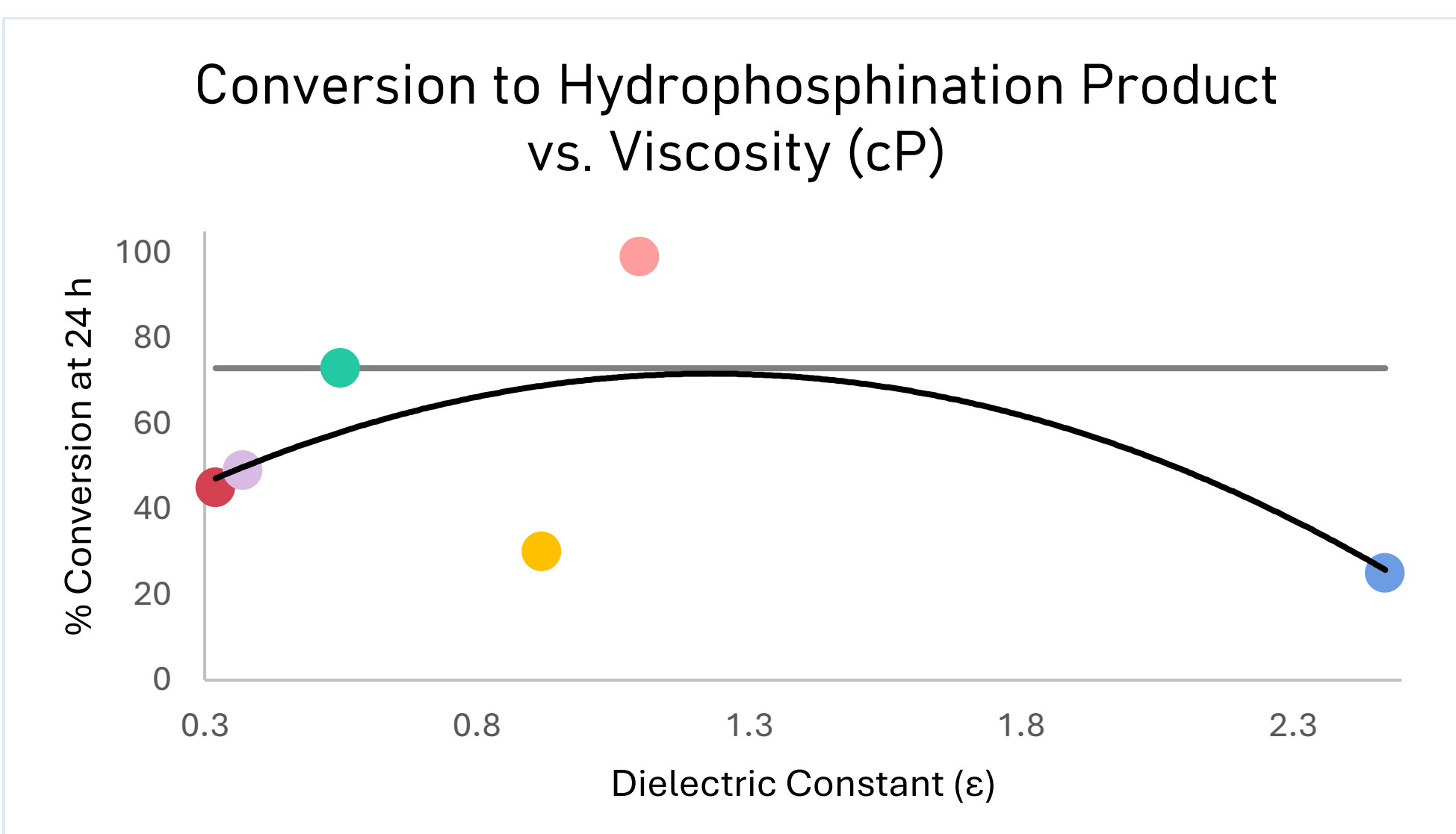

Figure S 1. Product conversion vs viscosity of ethylacrylate and diphenylphosphine in solvent after 24 h.

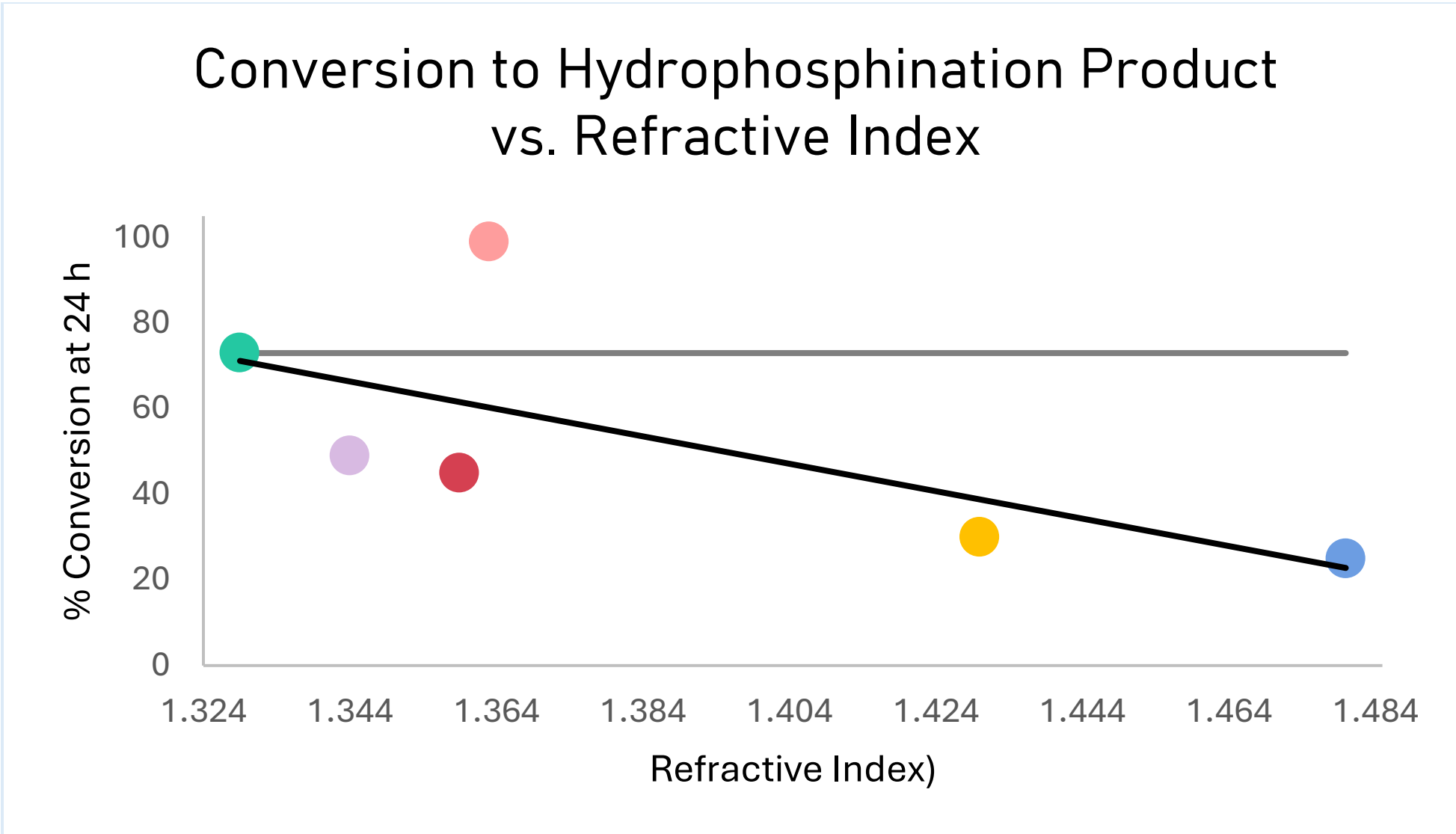

Figure S 2. Product conversion vs refractive index of ethylacrylate and diphenylphosphine in solvent after 24 h.

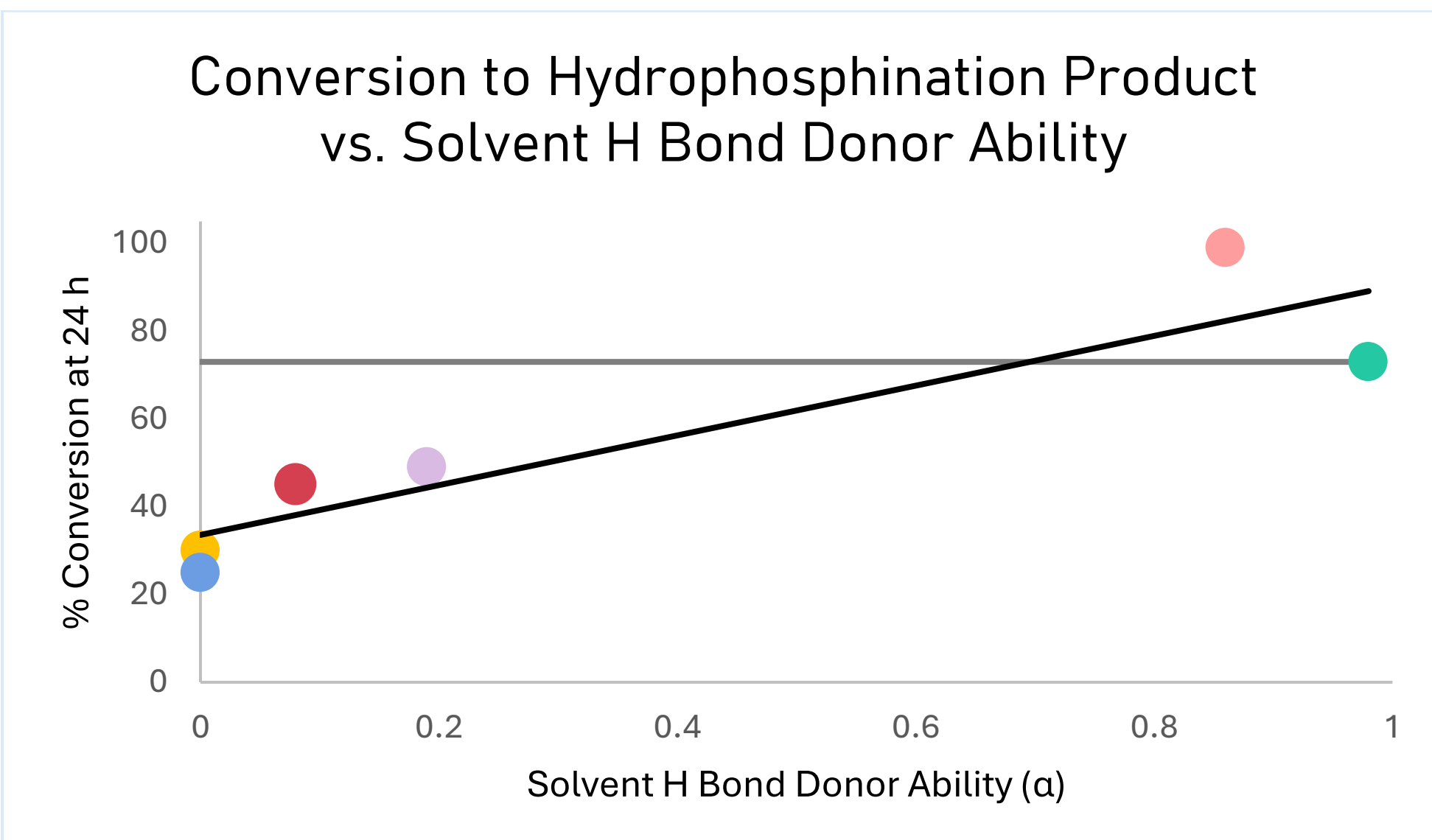

Figure S 3. Product conversion vs H-bond donor ability of ethylacrylate and diphenylphosphine in solvent after 24 h.

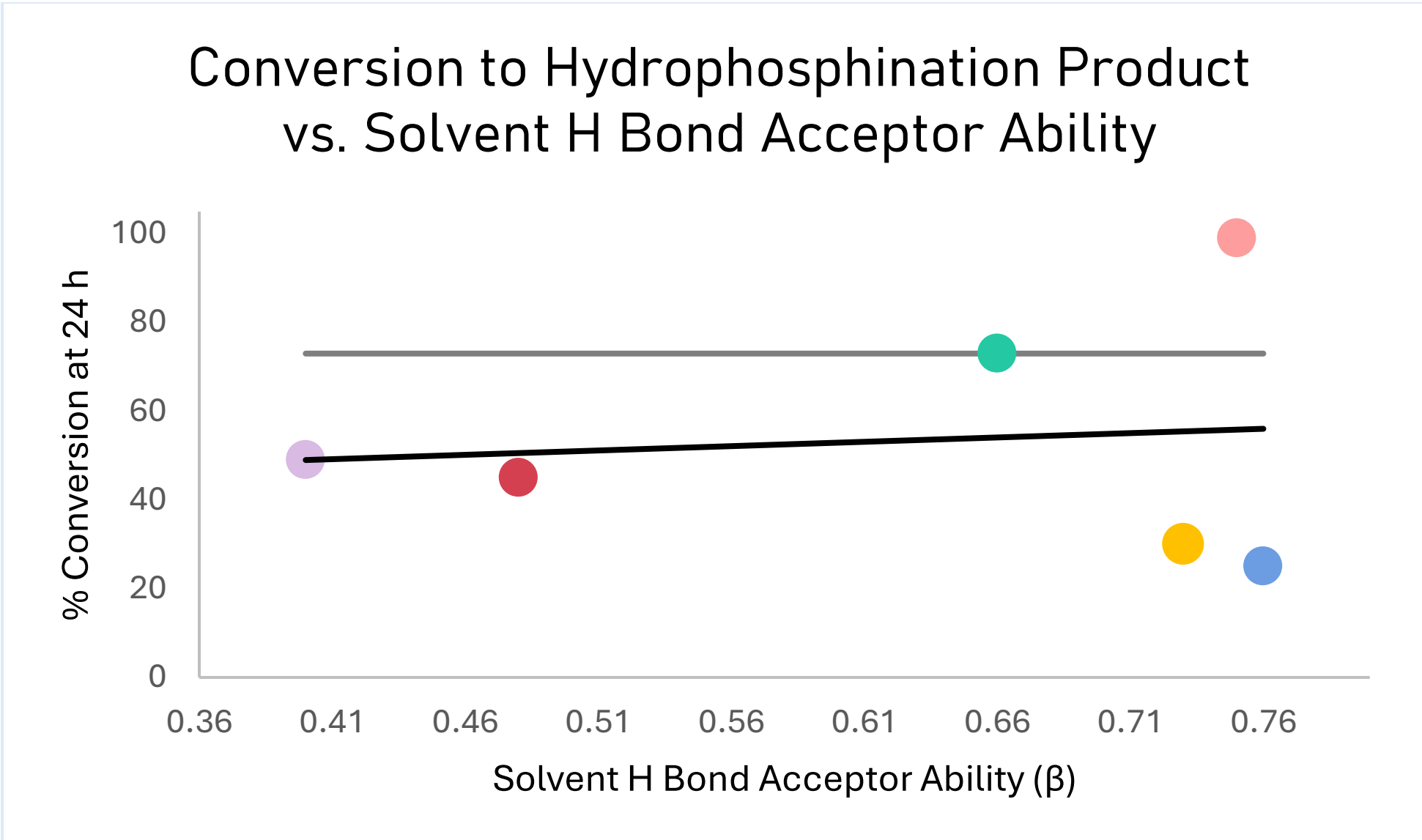

Figure S 4. Product conversion vs H-bond acceptor ability of ethylacrylate and diphenylphosphine in solvent after 24 h.

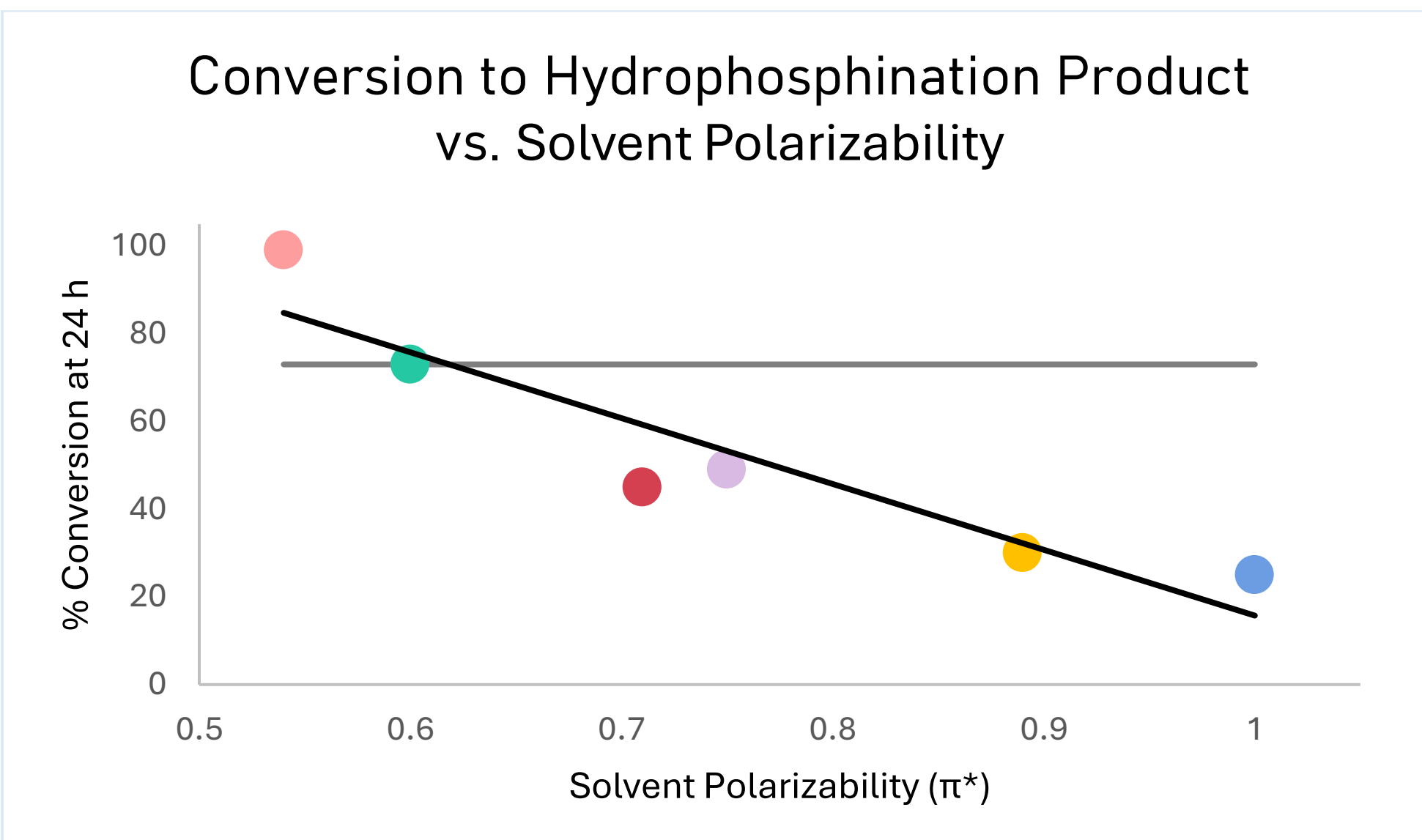

Figure S 5. Product conversion vs solvent polarizability of ethylacrylate and diphenylphosphine in solvent after 24 h.

Hydrophosphination of styrene with diphenylphosphine in various solvents

Entry	Solvent	Dielectric Constant (ε)	Conv. (%)
1	Acetone-d_6	20.7	15
2	neat	0	61
3	DMF	36.7	28

4	MeCN	37.5	23
5	C_6H_6	2.27	62
6	C_6H_5Br	5.17	98
7	DMSO-d_6	46.7	63
8	n-BuOH	17.5	83
9	n-PrOH	20.3	94
10	EtOH	24.5	96
11	MeOH	32.7	80

Reaction condition: diphenylphosphine (0.38 mmol), styrene (0.38 mmol), solvent (0.4 mL). Conversion was determined by ^{31}P NMR spectroscopy.

Table S 3. Tabulated values of the hydrophosphination of styrene with diphenylphosphine in the selected solvents after 24 h irradiation at 360 nm.

Solvent effects on the hydrophosphination of styrene and diphenylphosphine in various solvents

Key:

Methanol
Acetonitrile
Acetone
Ethanol
DMF
DMSO

*Neat reaction is denoted by flat, dotted line.

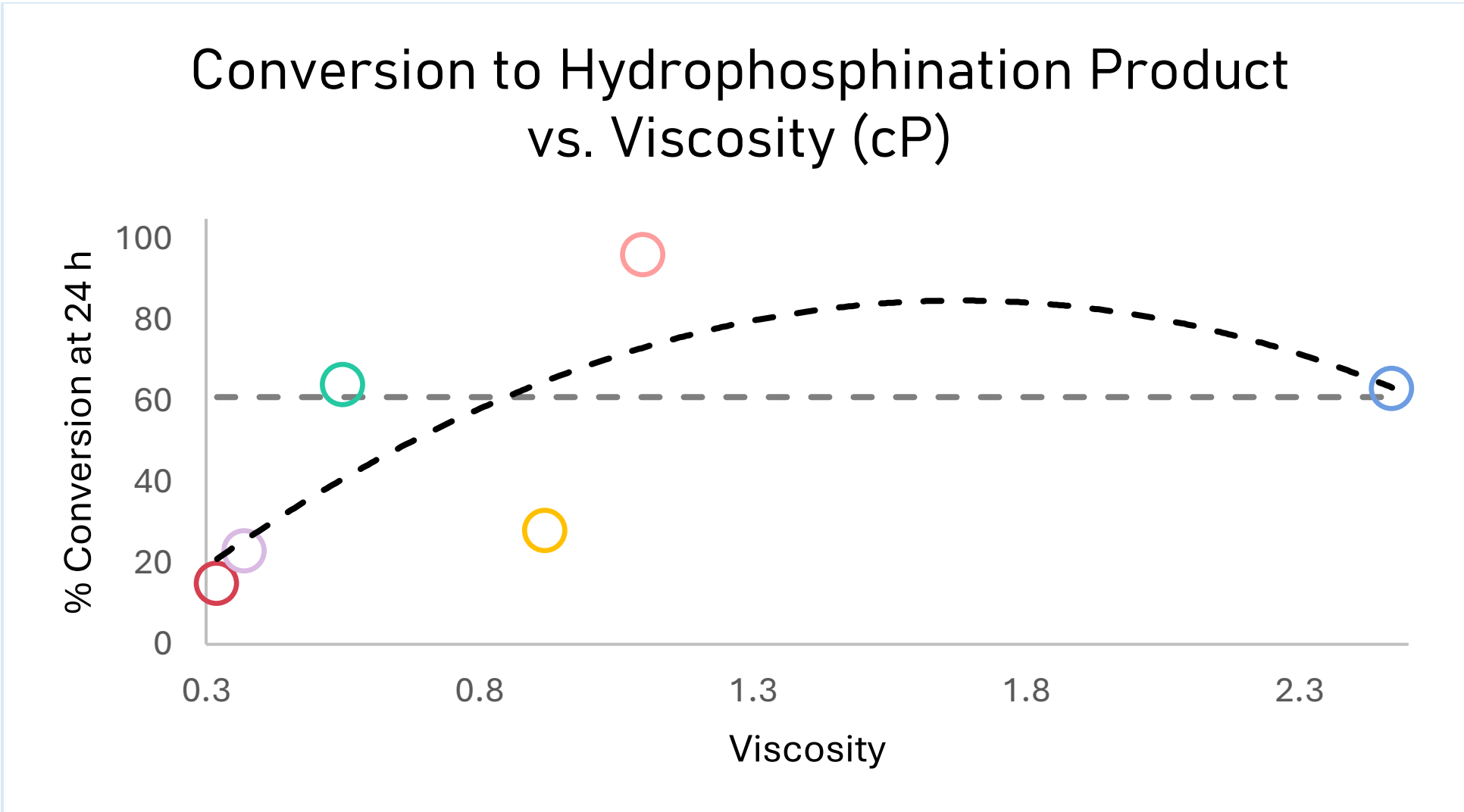

Figure S 6. Product vs viscosity of styrene and diphenylphosphine in solvent after 24 h.

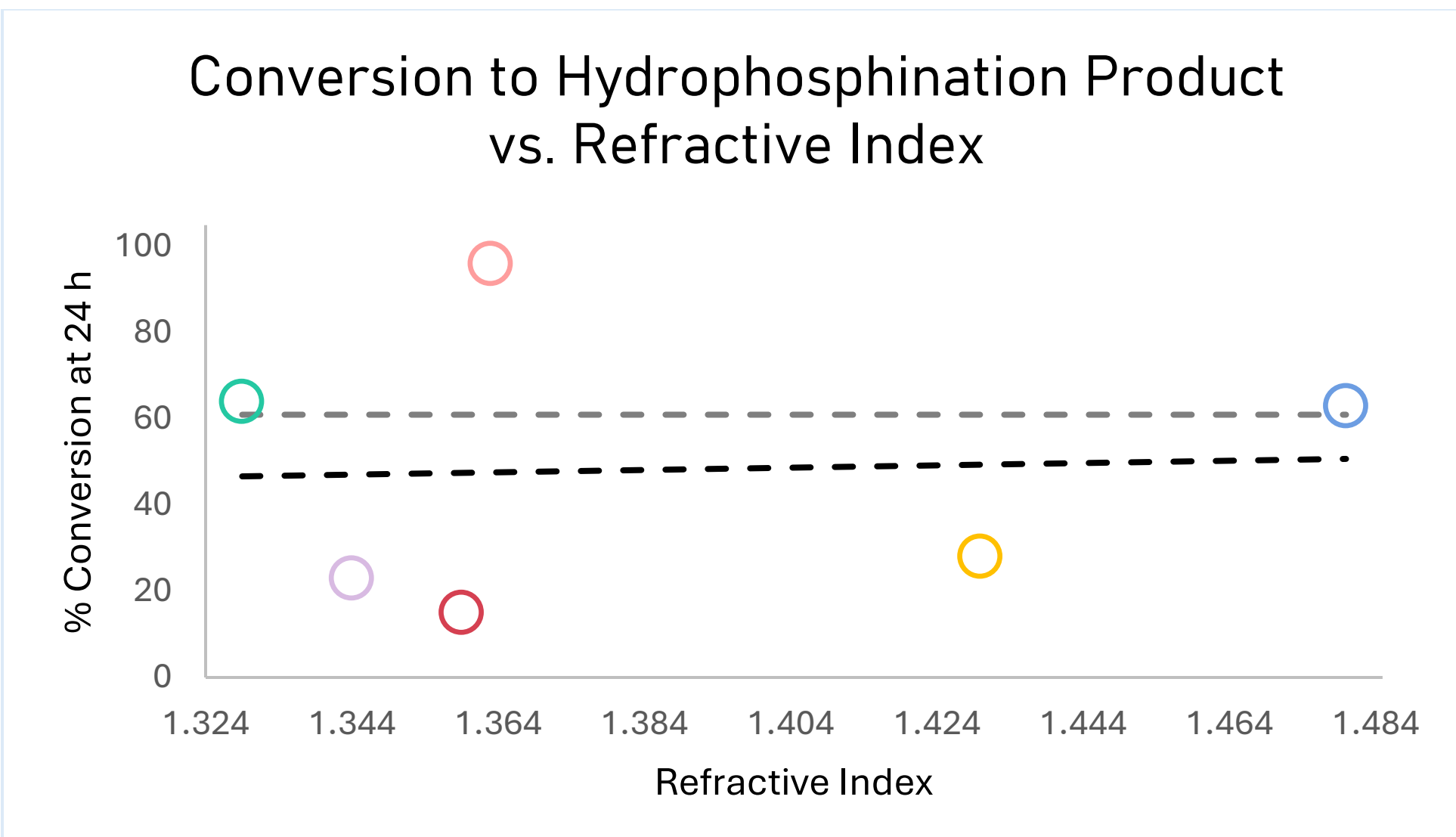

Figure S 7. Product vs refractive index of styrene and diphenylphosphine in solvent after 24 h.

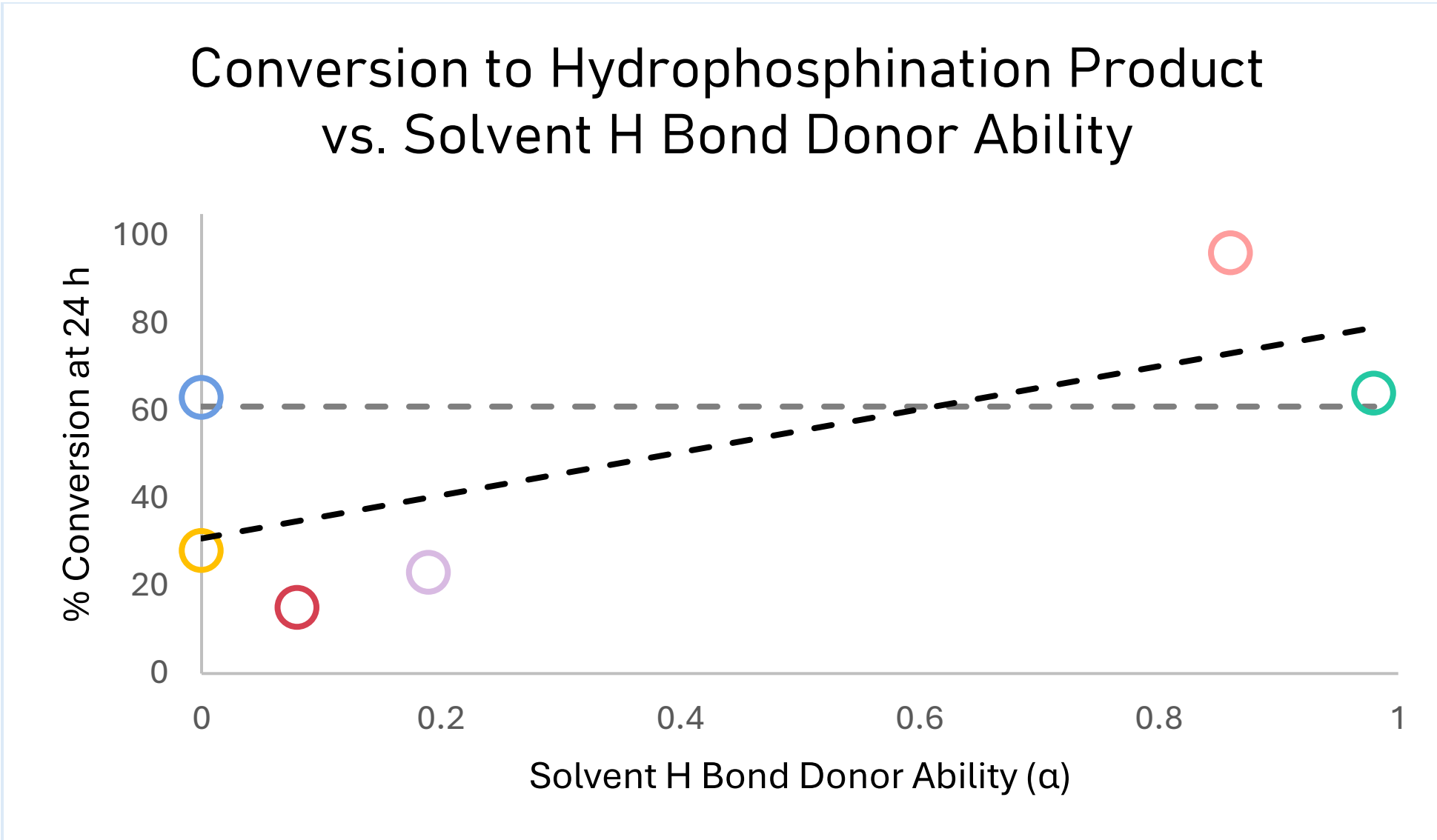

Figure S 8. Product vs H-donor ability of styrene and diphenylphosphine in solvent after 24 h.

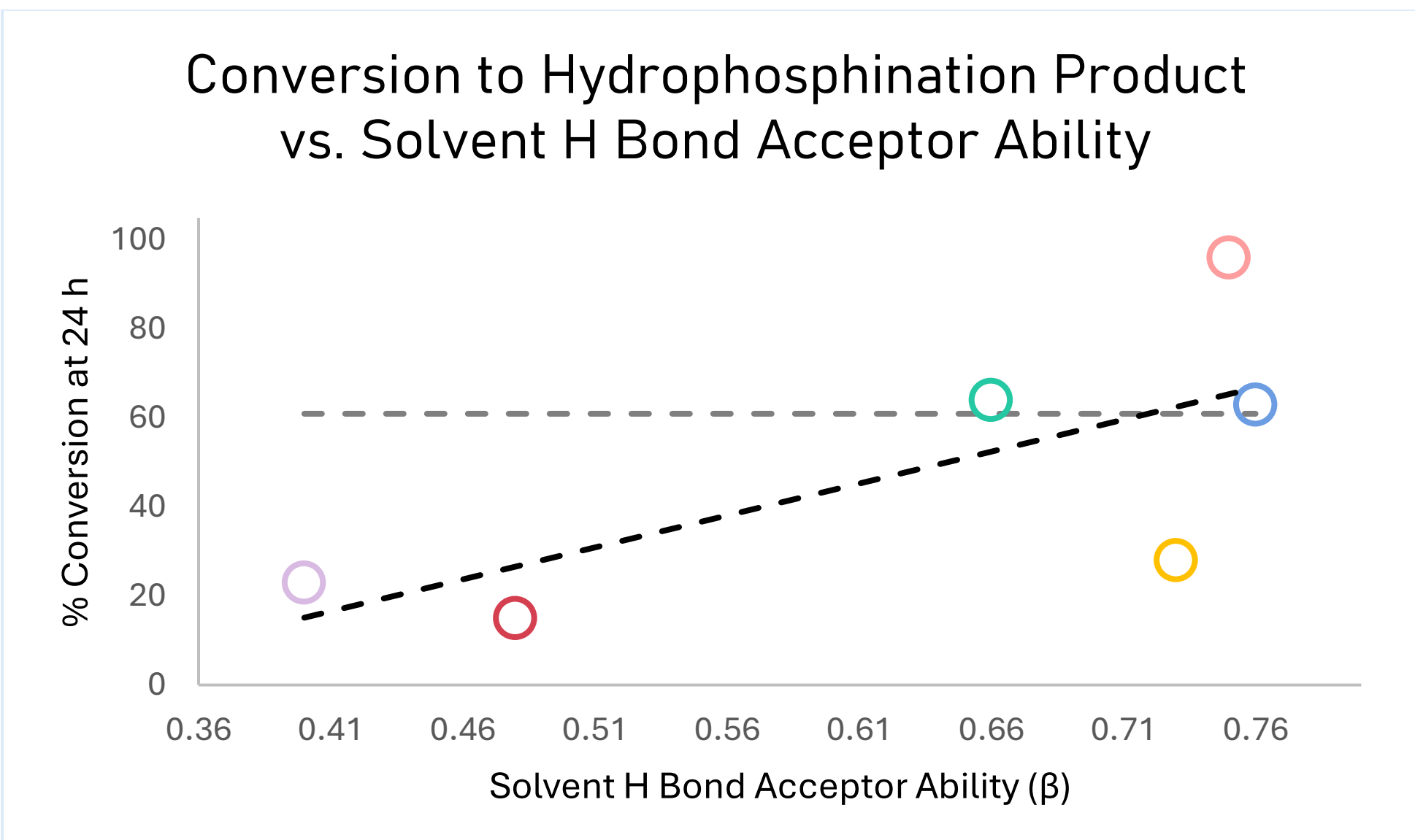

Figure S 9. Product vs H-bond acceptor ability of styrene and diphenylphosphine in solvent after 24 h.

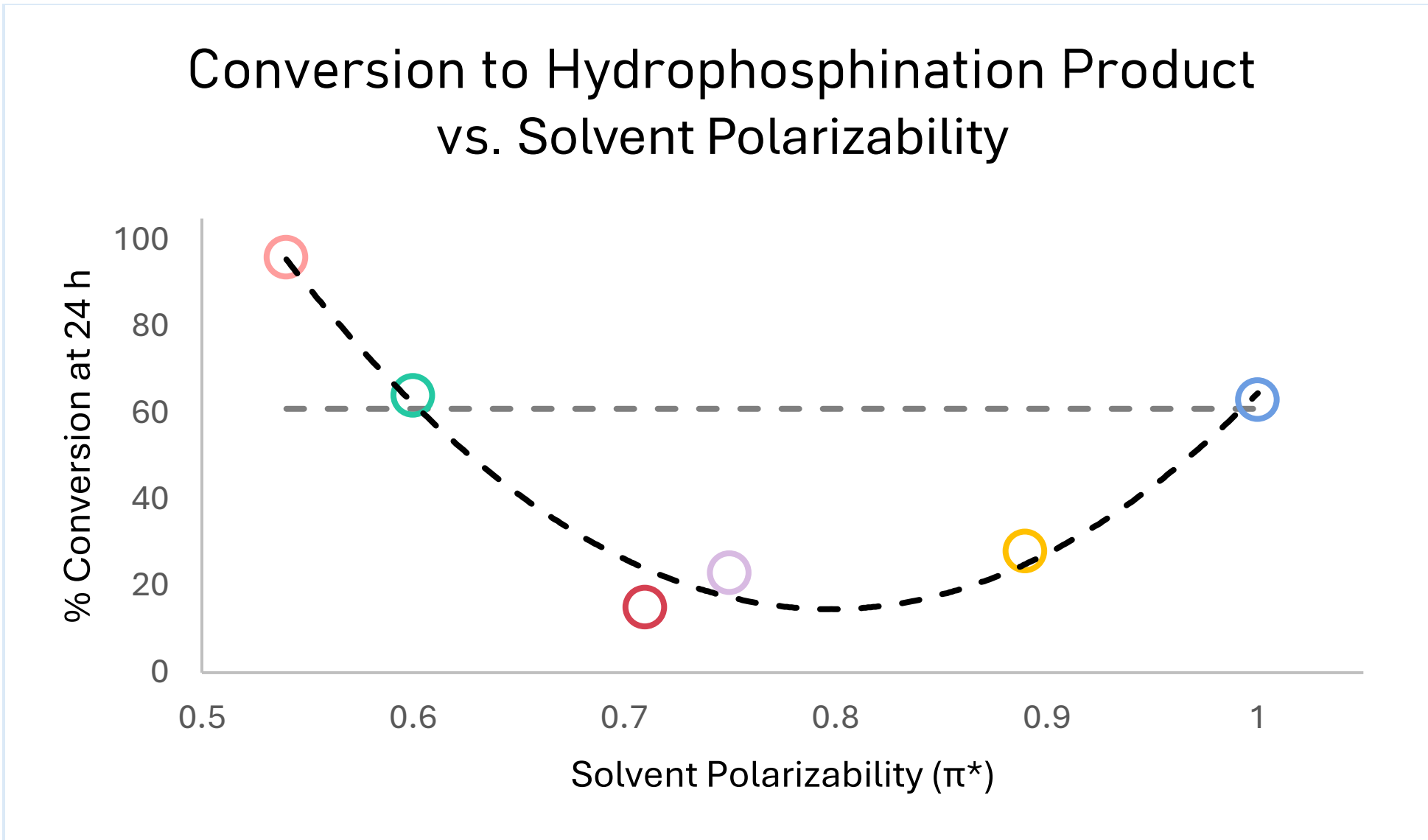

Figure S 10. Product vs solvent polarizability of styrene and diphenylphosphine in solvent after 24 h.

Deuterium Labelling Experiments

Synthesis of diphenylphosphine-d_1

A solution of PPh_3 (25 mmol) was dissolved in 25 mL of dry THF in a flame dried 100 mL Schlenk flask, and Na (75 mmol, 1,73 g) was then added to stir at room temperature for 24 h. The suspension turned from clear to dark ruby, and the resulting mixture was transferred away from the solid Na via cannula into a flame dried 100 mL flask. After cooling the flask to 0 °C, D_2O (2 mL) was added, and the mixture was stirred until the solution turned colorless. Hexane (50 mL) was added to the solution for extraction, and the mixture was allowed to stir for 30 min. The organic layer was transferred into a clean 100 mL Schlenk flask, and volatiles were removed *in vacuo*. The pure product was obtained as a clear liquid after the distillation of the dry organic layer at 130 °C under reduced pressure.

Deuterium labelling with diphenylphosphine-d_1

General procedure: Diphenylphosphine-d_1 (0.38 mmol) was added to a vial charged with styrene or ethyl acylate (0.038 mmol) and EtOH (0.4 mL). The solution was briefly mixed and then transferred to an NMR tube for NMR measurement.

Ethyl acrylate:
*note: number in parenthesis denotes %D-incorporation.

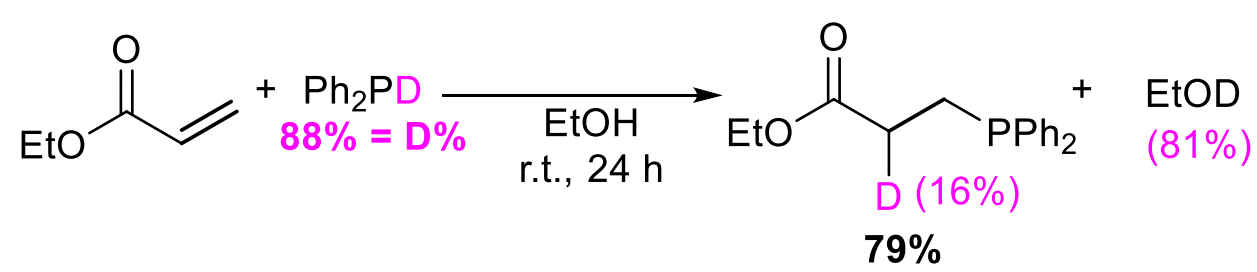

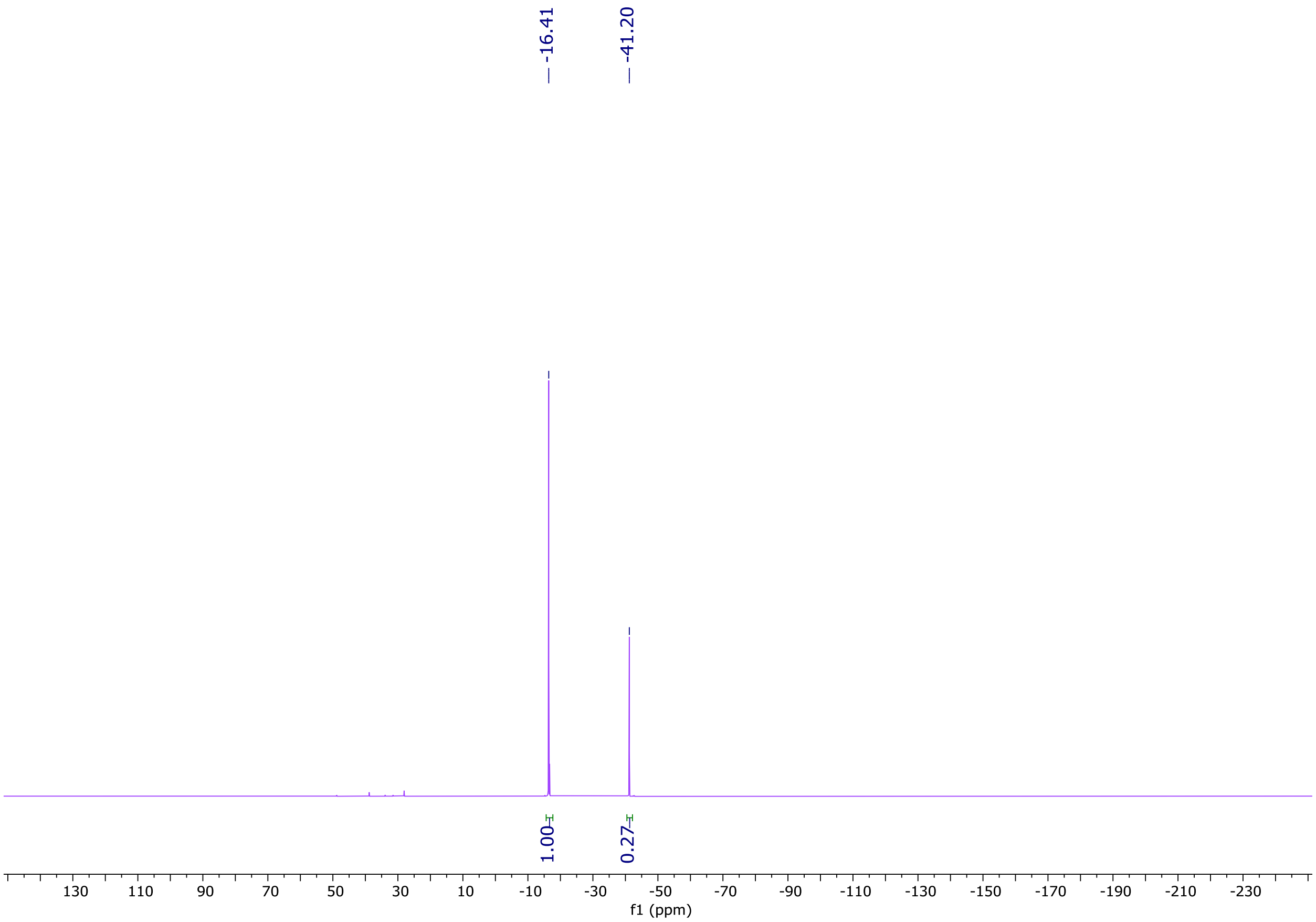

Figure S 11. $^{31}P\{^{1}H\}$ NMR spectrum (202 MHz, EtOH) of the reaction between ethyl acrylate and diphenylphosphine-d_1 in EtOH after 24 h (79% yield).

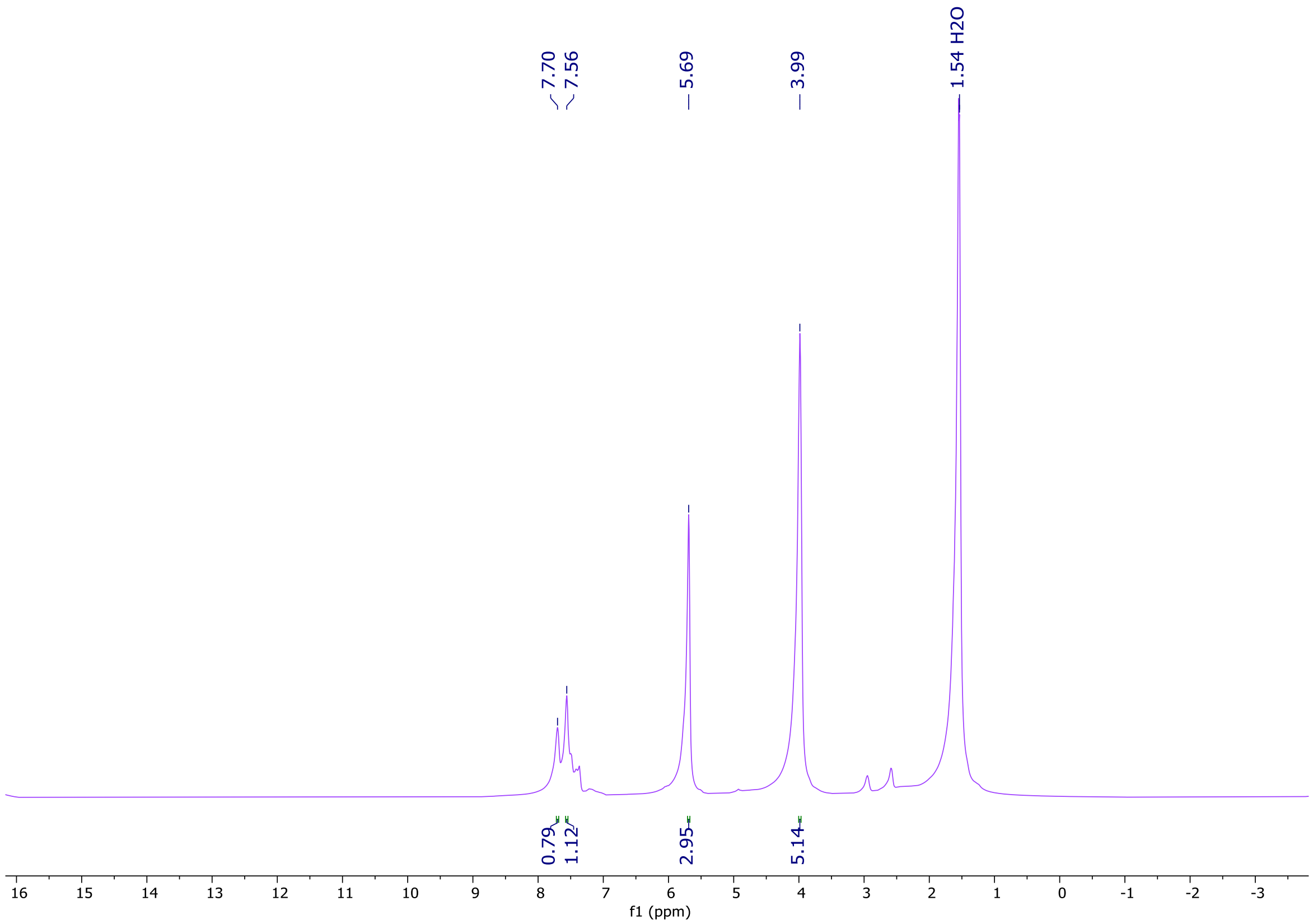

Figure S 12. ^1H NMR spectrum (500 MHz, EtOH) of the reaction between ethyl acrylate and diphenylphosphine-d_1 in EtOH after 24 h (79% yield).

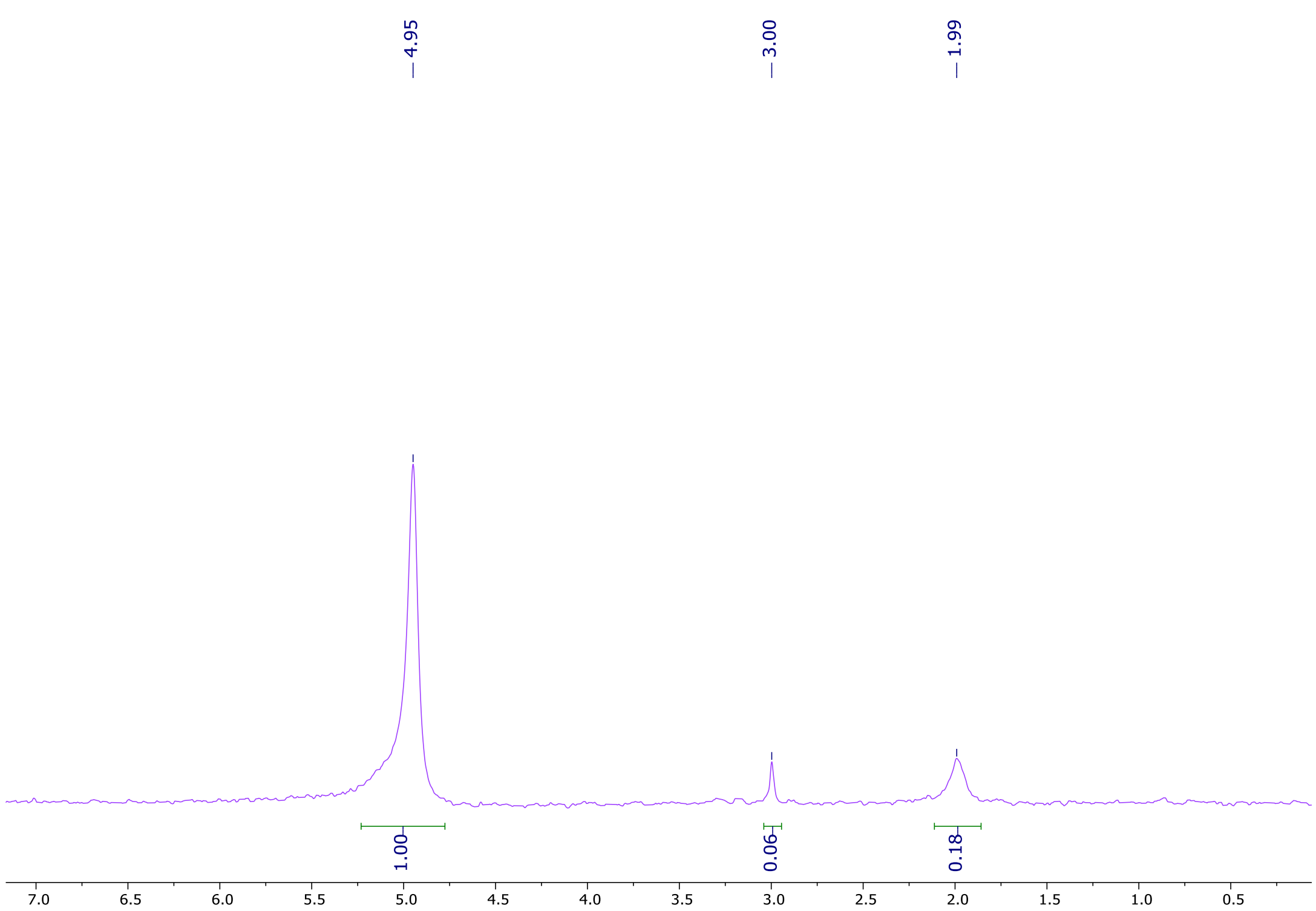

Figure S 13. ^2H NMR spectrum (77 MHz, EtOH) of the reaction between ethyl acrylate and diphenylphosphine-d_1 in EtOH after 24 h (79% yield).

Styrene:

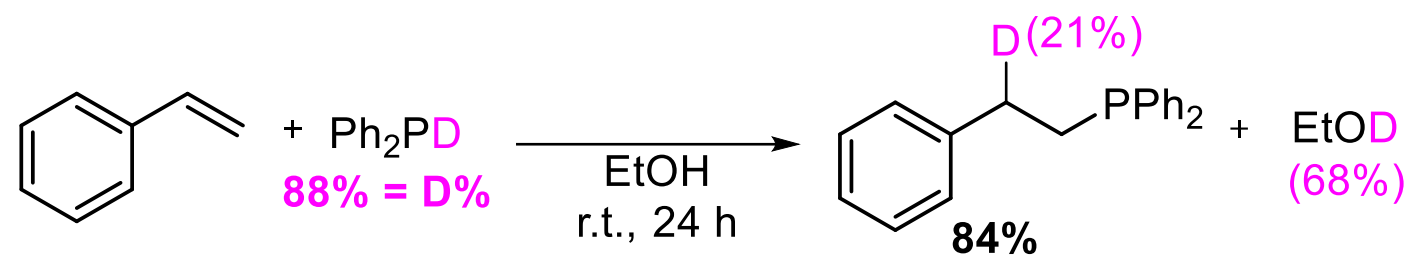

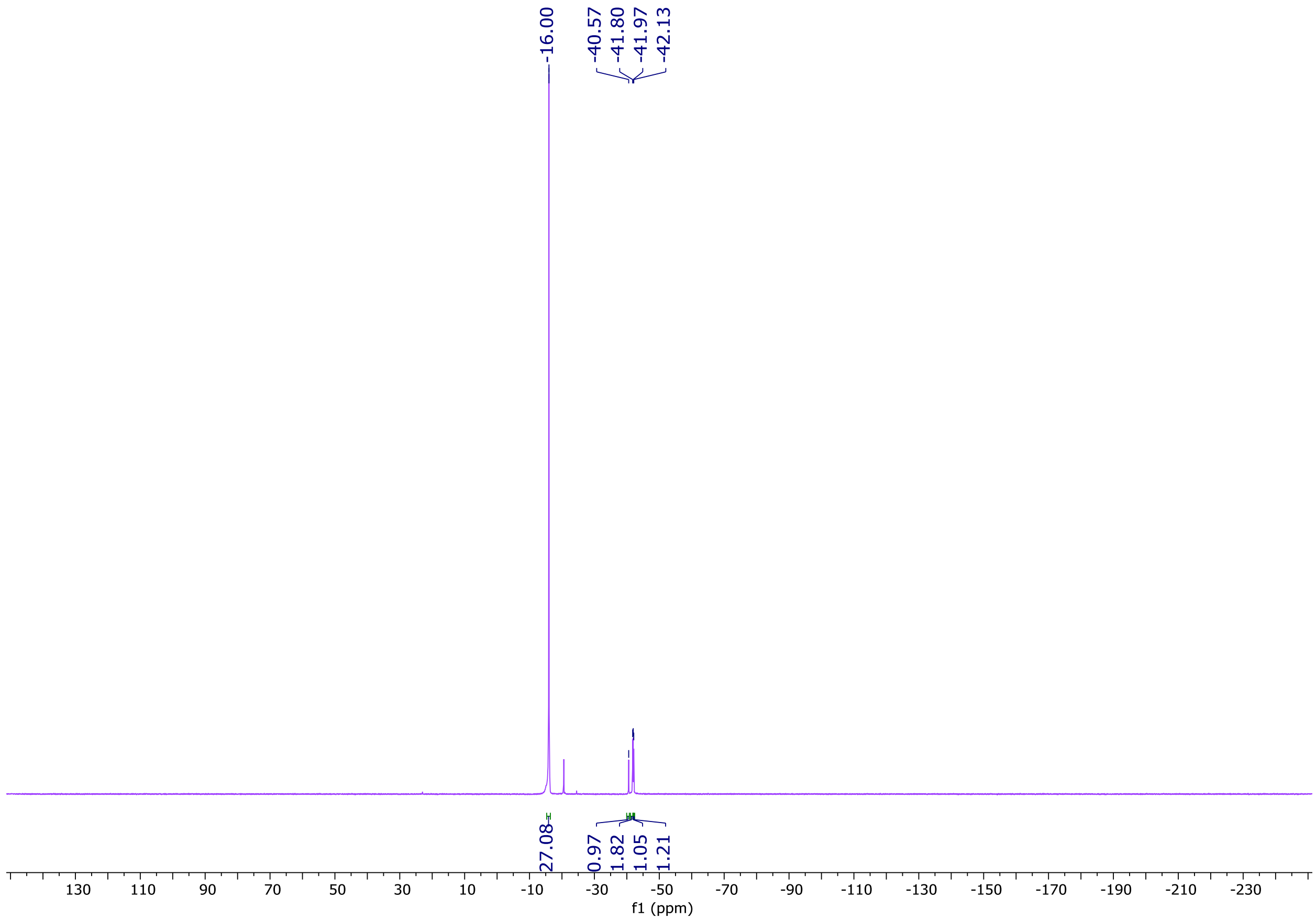

Figure S 14. $^{31}P\{^1H\}$ NMR spectrum (202 MHz, EtOH) of the reaction between styrene and diphenylphosphine-d_1 in EtOH after 24 h.

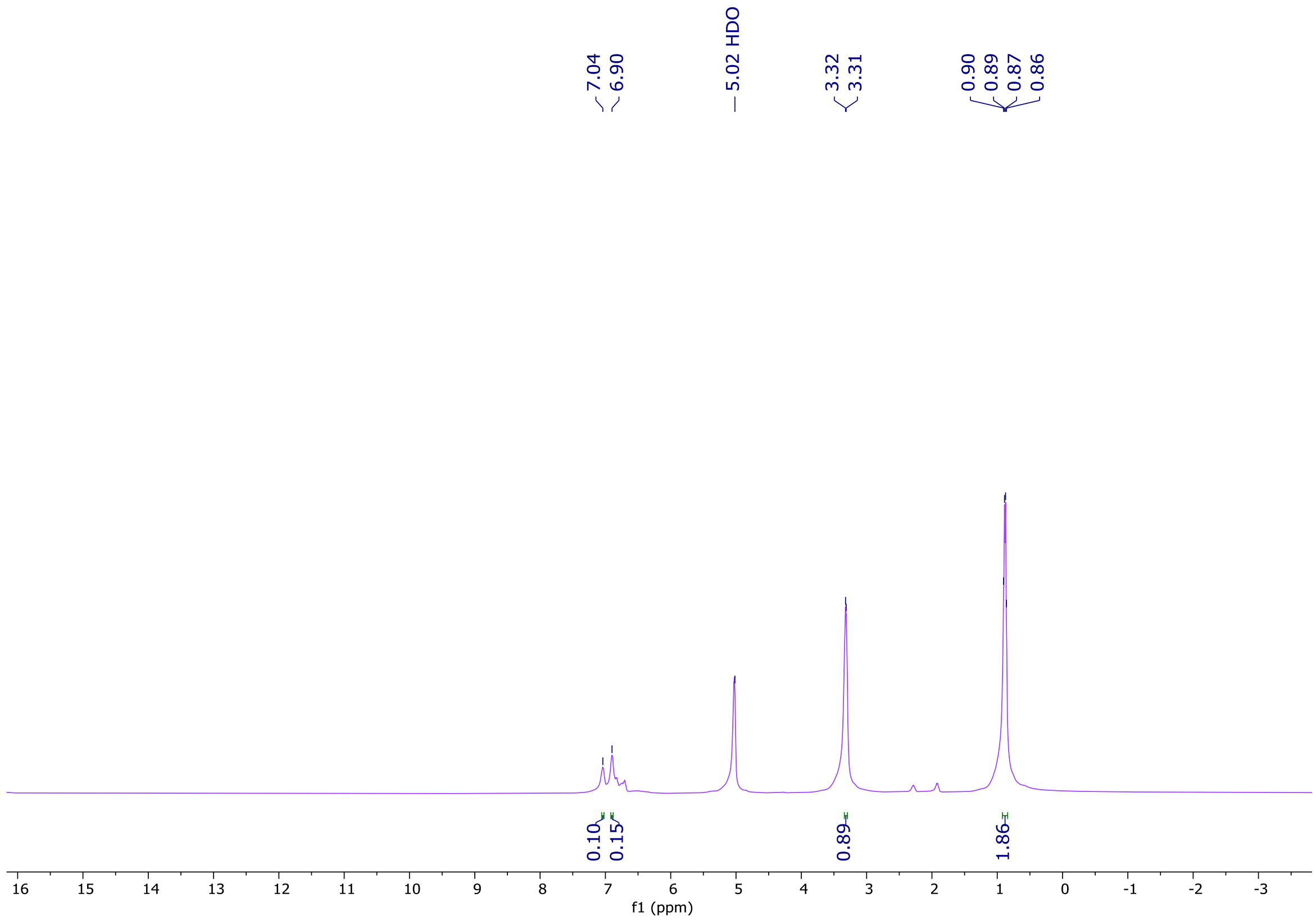

Figure S 15. ^{1}H NMR spectrum (500 MHz, EtOH) of the reaction between styrene and diphenylphosphine-d$_1$ in EtOH after 24 h.

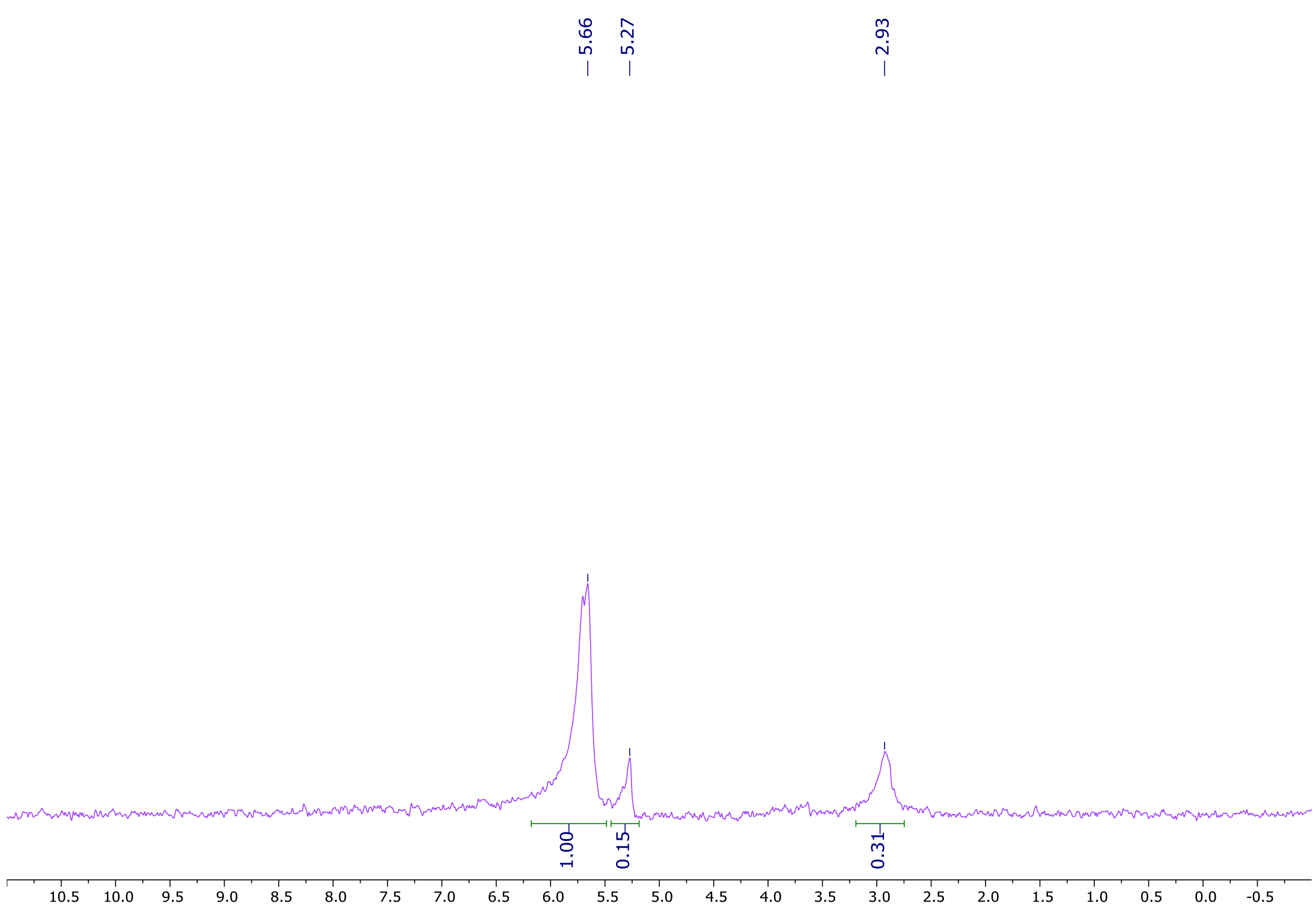

Figure S 16. ^{2}H NMR spectrum (77 MHz, EtOH) of the reaction between styrene and diphenylphosphine-*d*$_1$ in EtOH after 24 h.

In situ formation of diphenylphosphine-d_1

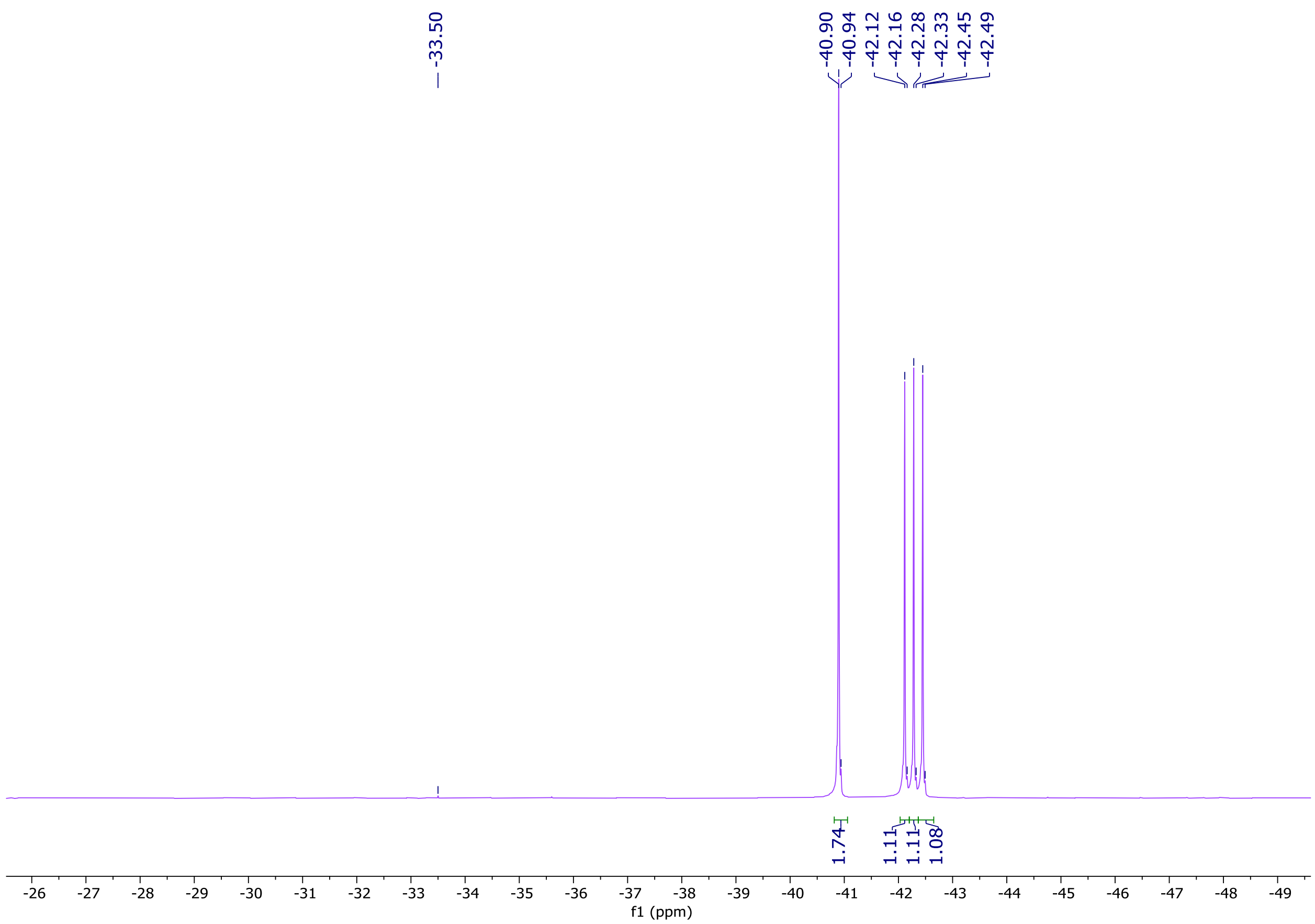

Figure S 17. $^{31}P\{^1H\}$ NMR spectrum (202 MHz, MeOH) of and diphenylphosphine in methanol-d_4 under 360 nm irradiation after 24 h.

UV/Visible spectra of Ph_2PH in EtOH

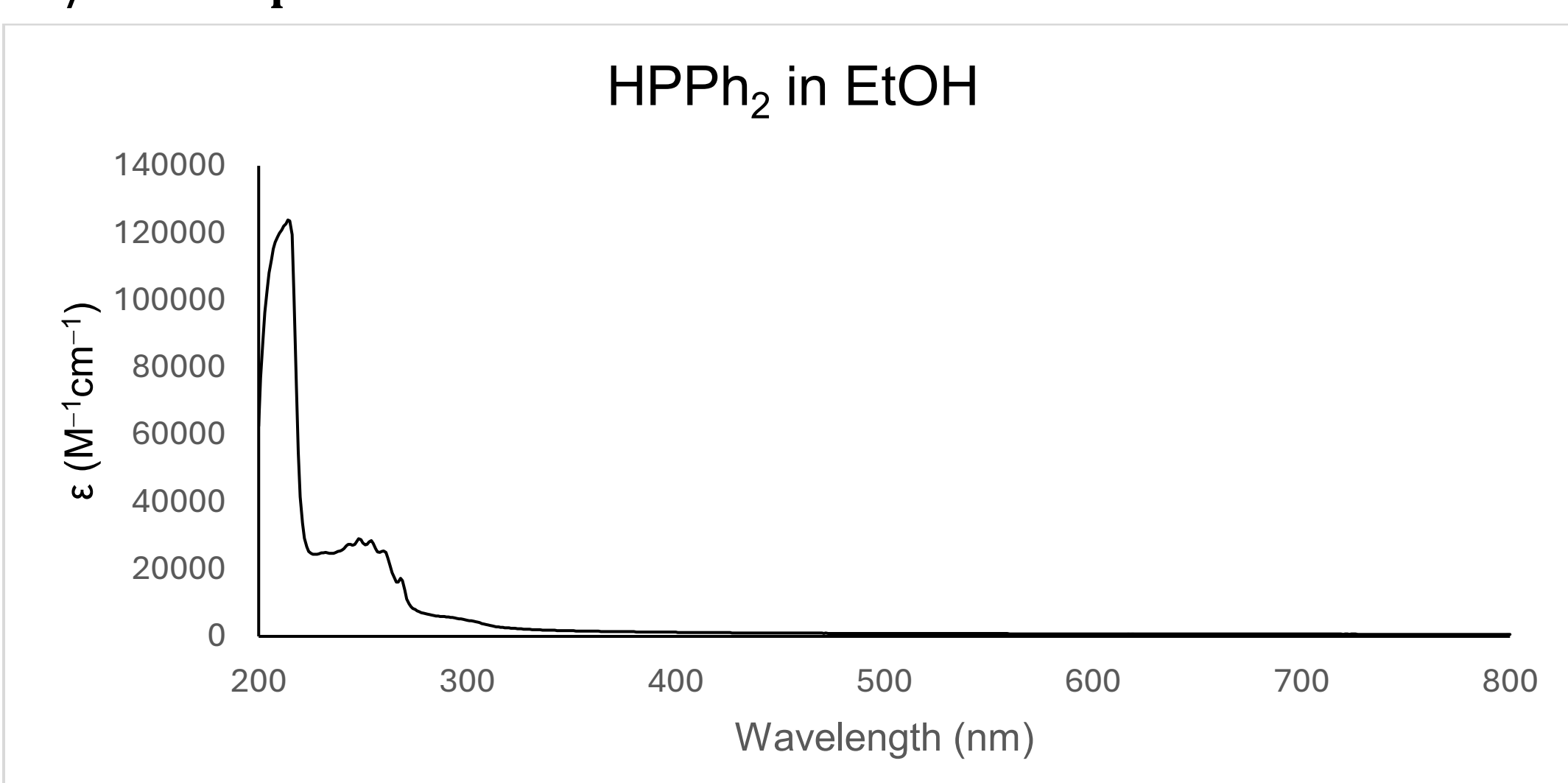

Figure S 18. UV/Vis spectra of diphenylphosphine in EtOH (2.69 x 10^{-5} M).

NMR spectra for catalyst-free reactions

Hydrophosphination of ethyl acrylate in various solvents

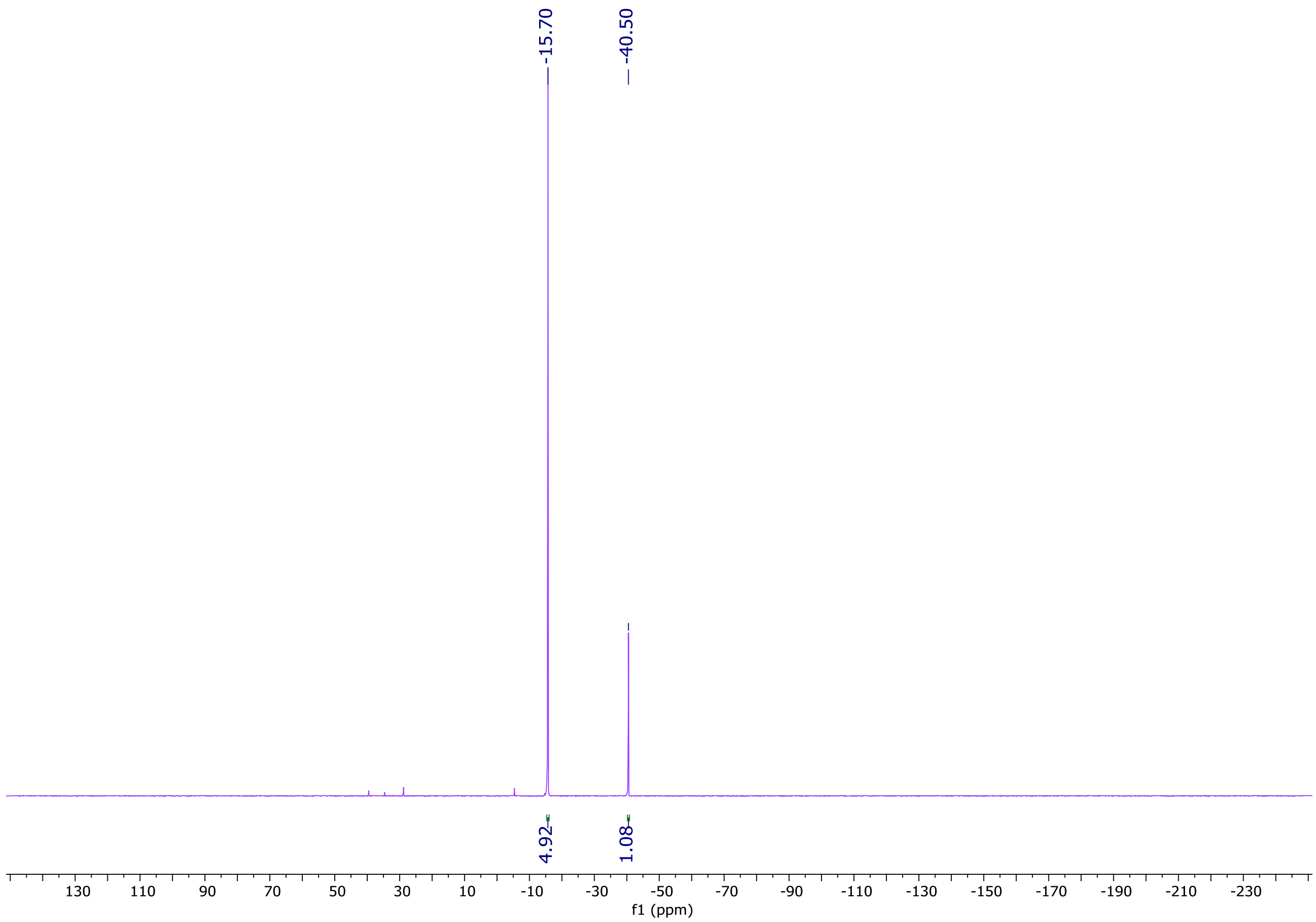

Figure S 19. $^{31}P\{^1H\}$ NMR spectrum (202 MHz, MeOH) of **11** (ethyl acrylate and diphenylphosphine) after 24 h.

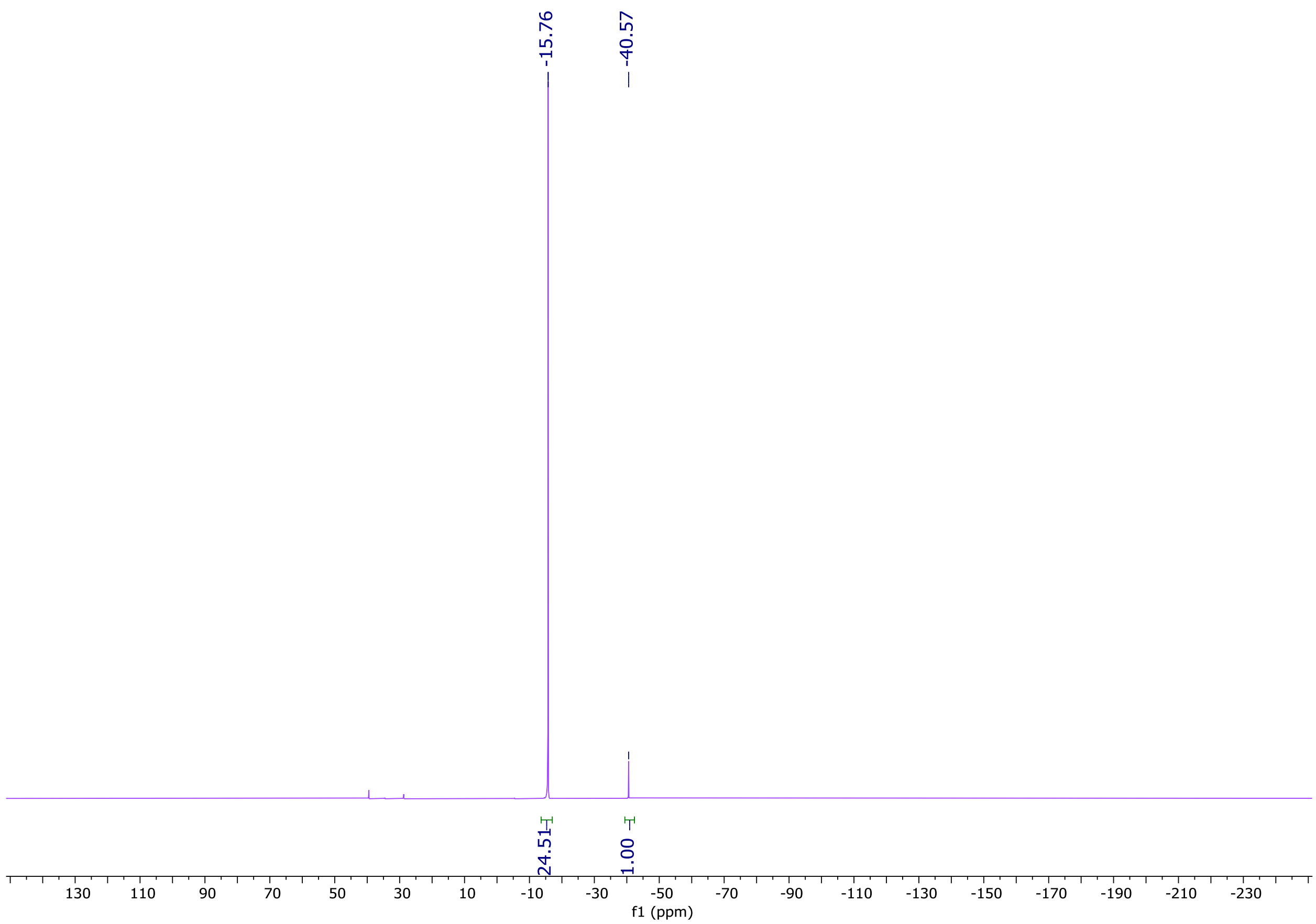

Figure S 20. ^{31}P NMR{^{1}H} spectrum (202 MHz, EtOH) of **11** (ethyl acrylate and diphenylphosphine) after 24 h.

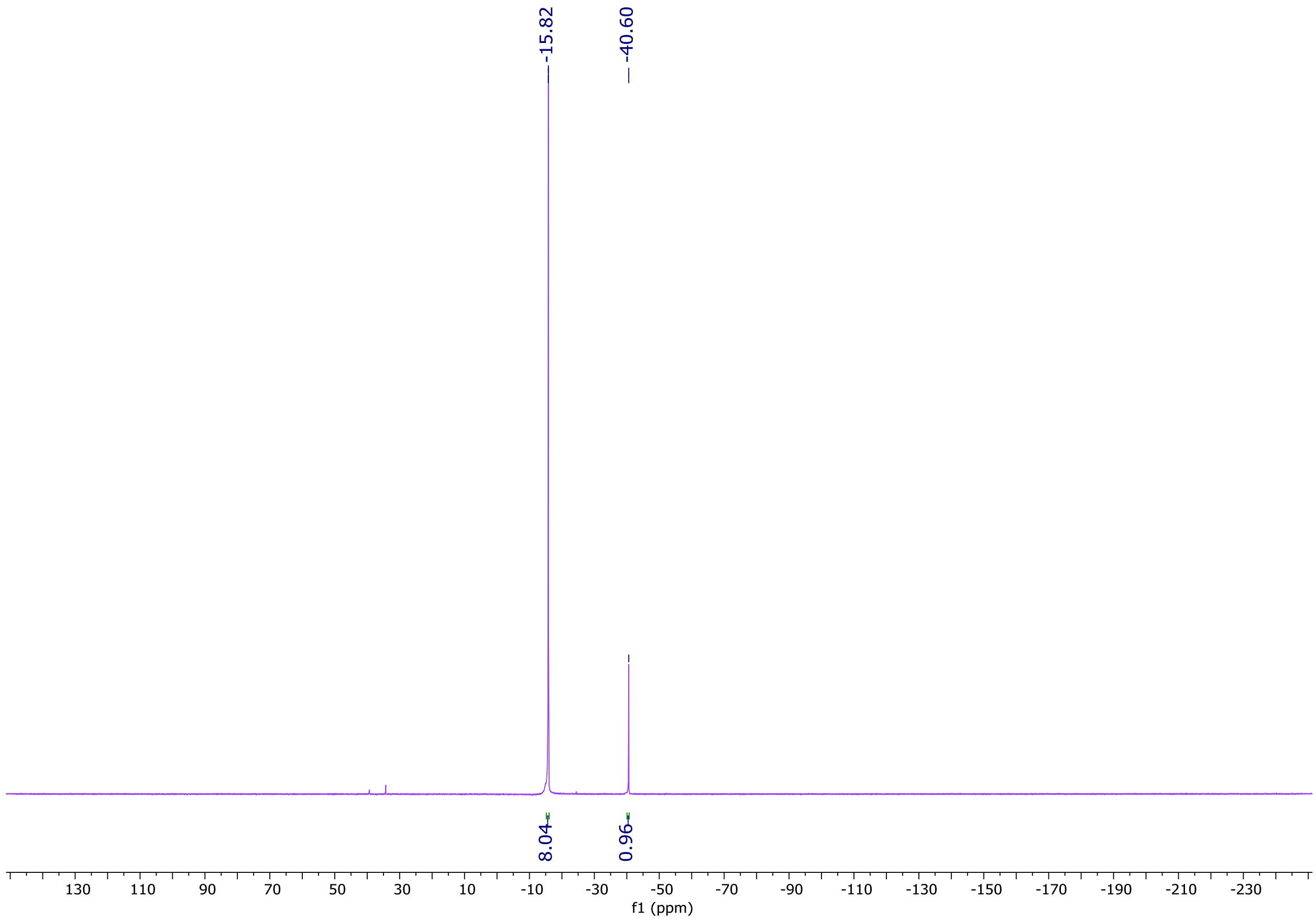

Figure S 21. $^{31}P\{^1H\}$ NMR spectrum (202 MHz, n-PrOH) of **11** (ethyl acrylate and diphenylphosphine) after 24 h.

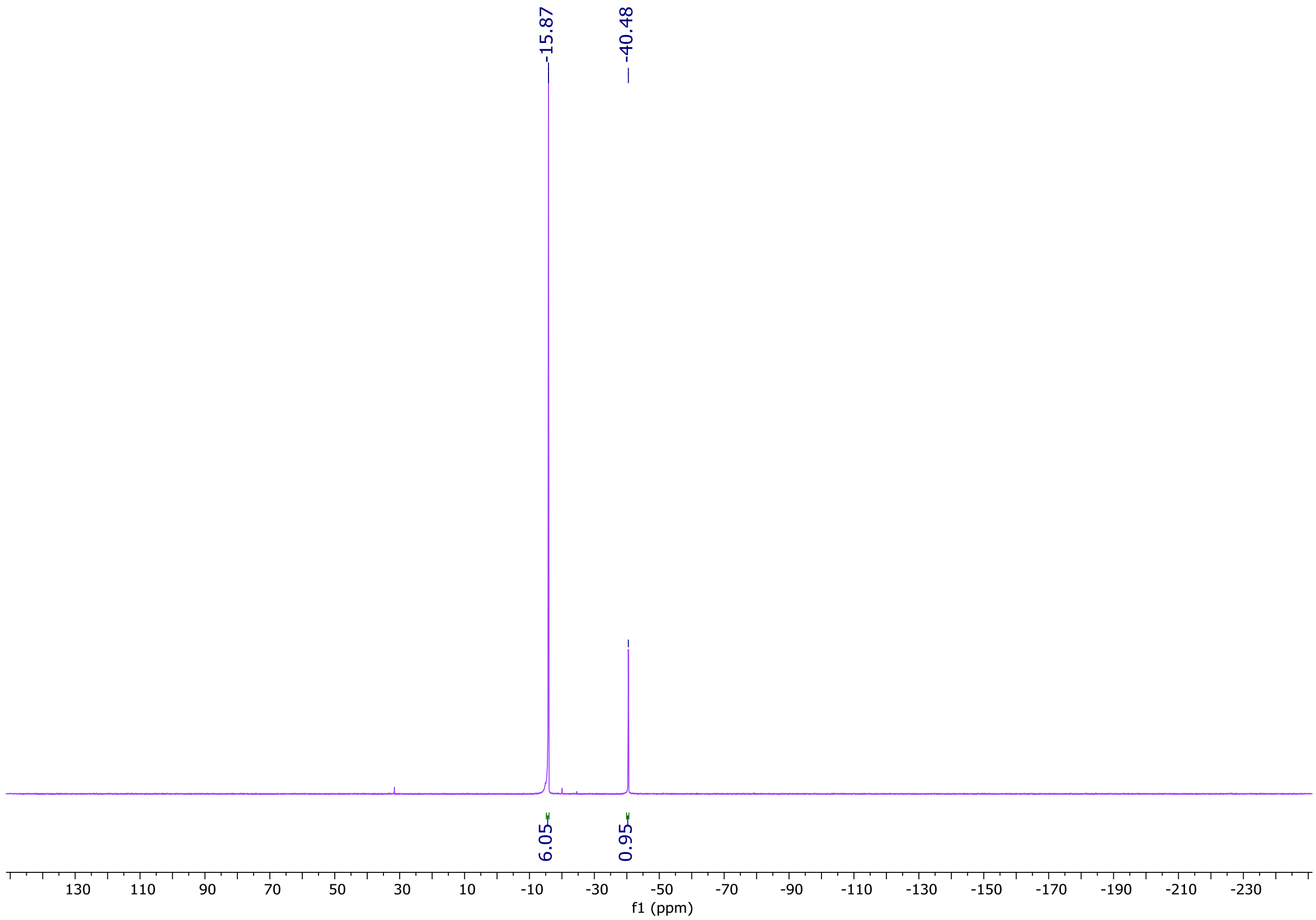

Figure S 22. $^{31}P\{^{1}H\}$ NMR spectrum (202 MHz, n-BuOH) of **11** (ethyl acrylate and diphenylphosphine) after 24 h.

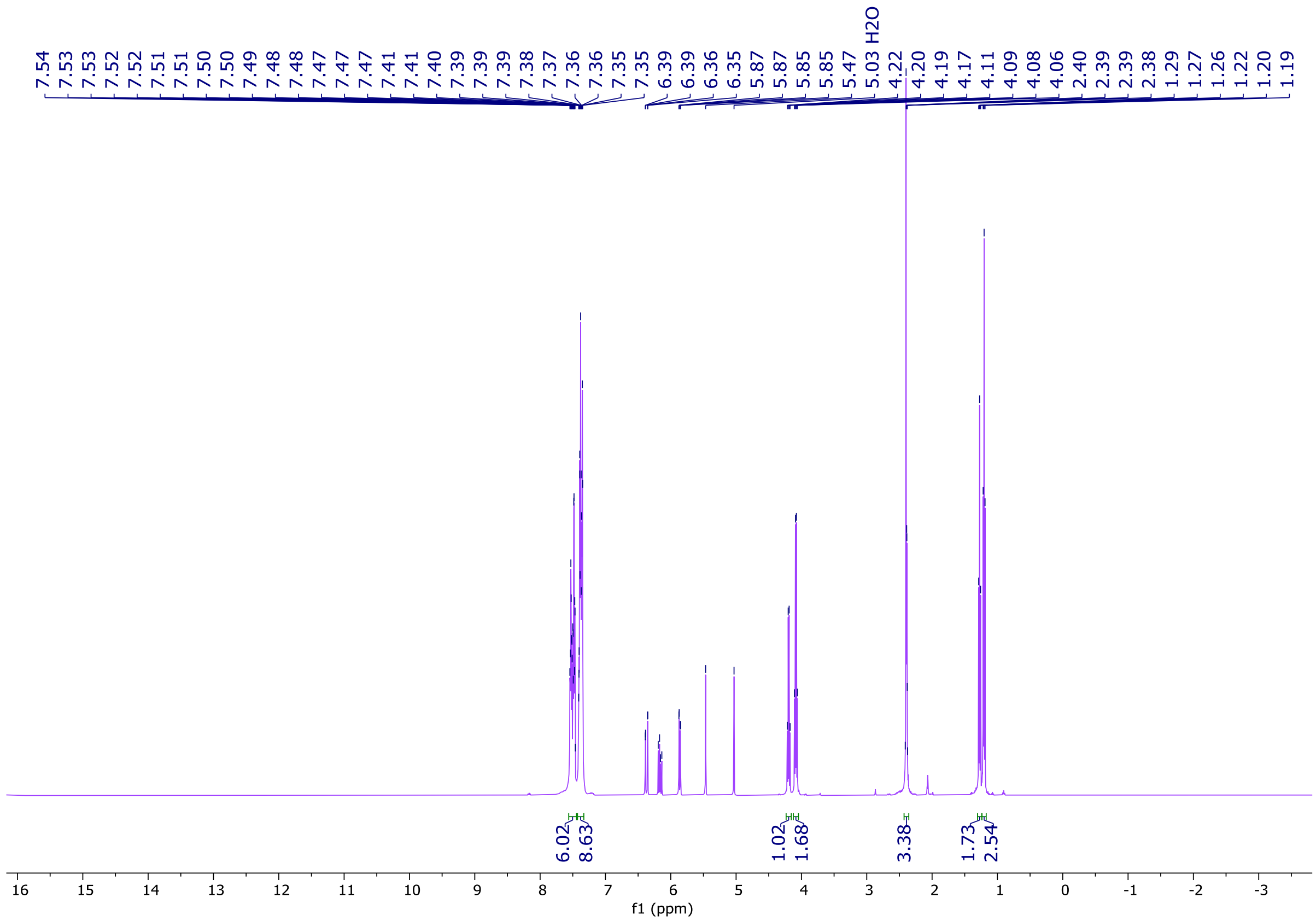

Figure S 23. ^{1}H NMR spectrum (500 MHz, Acetone-d6) of **11** (ethyl acrylate and diphenylphosphine) after 24 h.

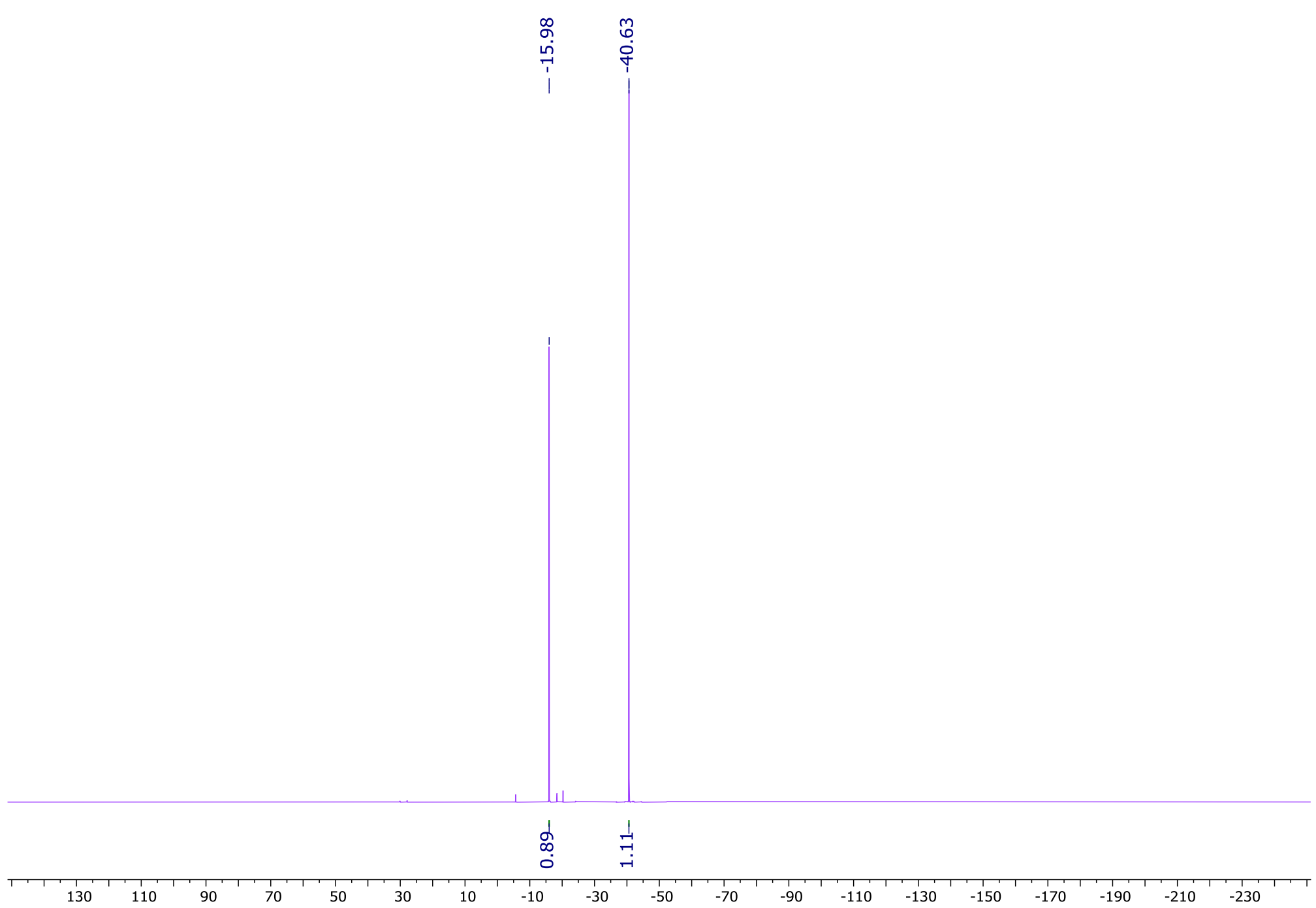

Figure S 24. $^{31}P\{^1H\}$ NMR spectrum (202 MHz, Acetone) of **11** (ethyl acrylate and diphenylphosphine) after 24 h.

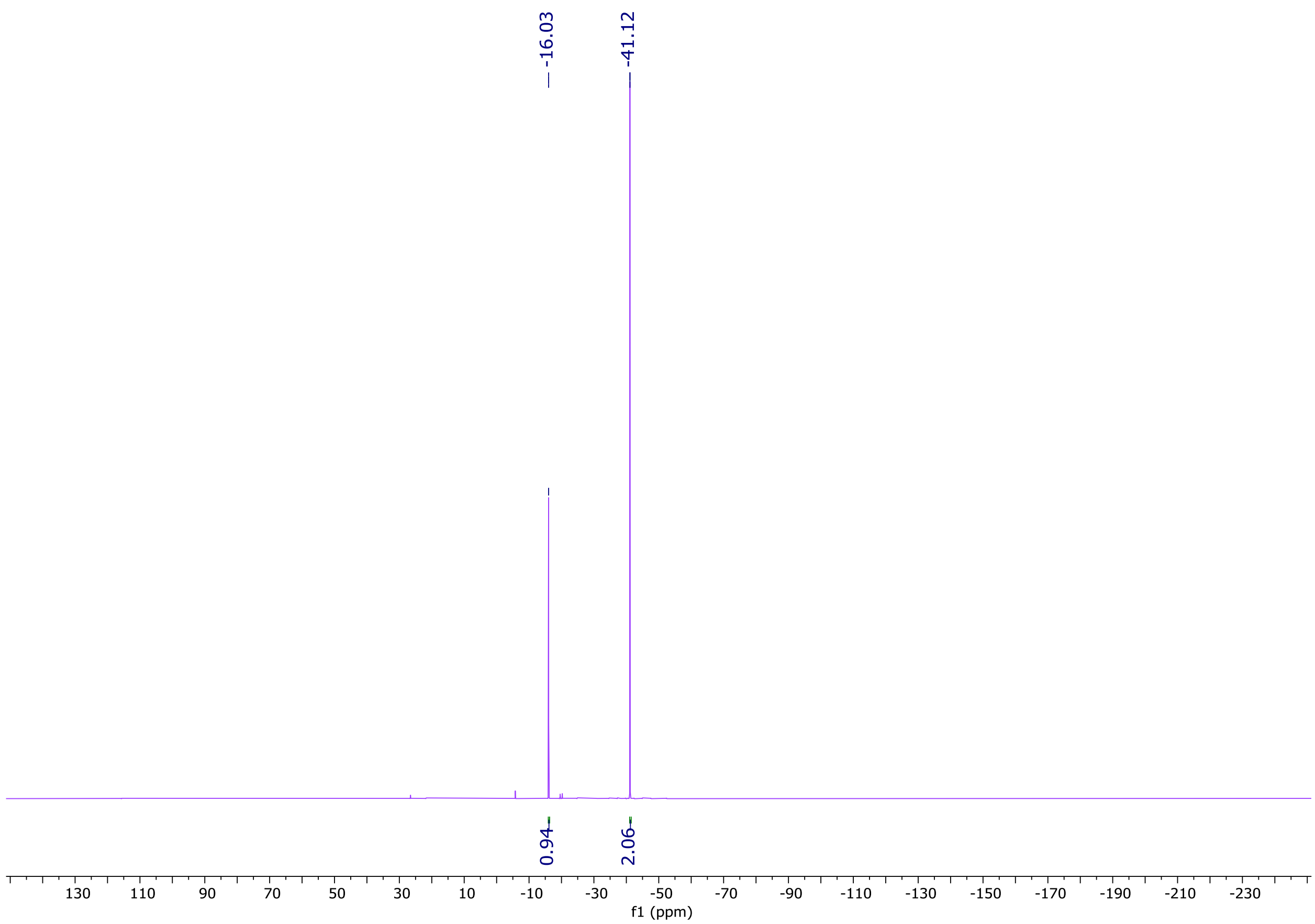

Figure S 25. $^{31}P\{^{1}H\}$ NMR spectrum (202 MHz, DMF) of **11** (ethyl acrylate and diphenylphosphine) after 24 h.

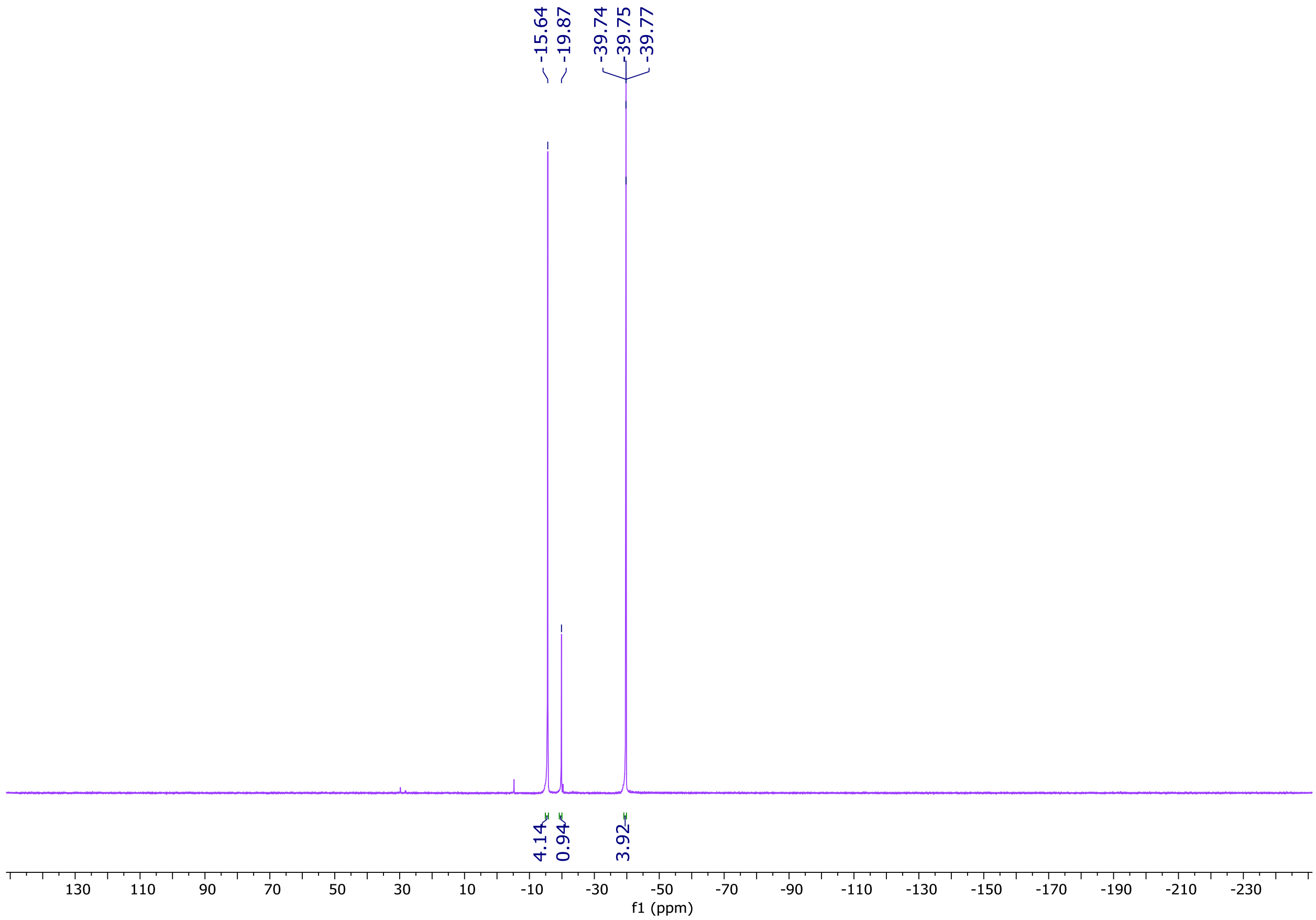

Figure S 26. $^{31}P\{^1H\}$ NMR spectrum (202 MHz, MeCN) of **11** (ethyl acrylate and diphenylphosphine) after 24 h.

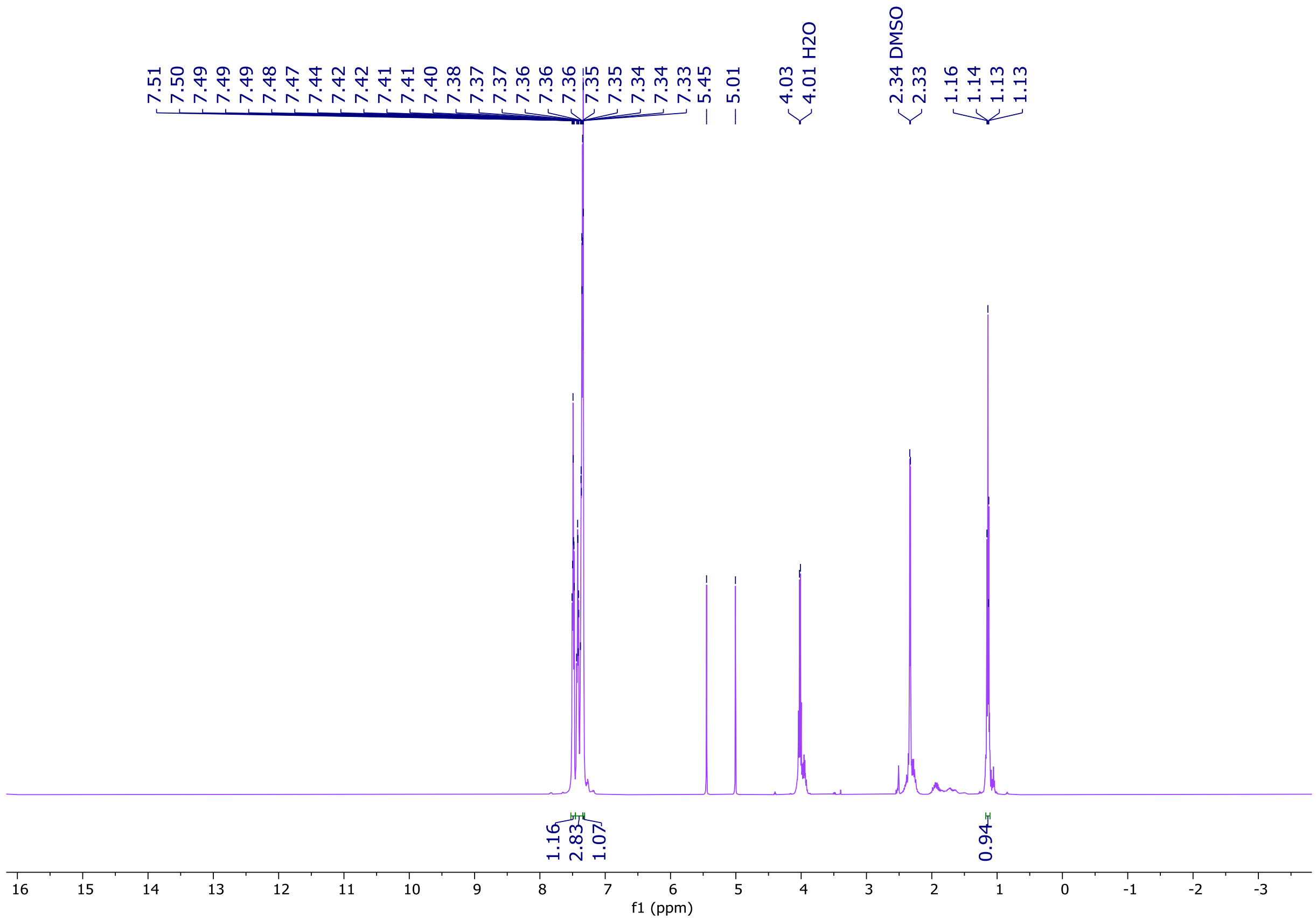

Figure S 27. ^{1}H NMR spectrum (500 MHz, DMSO-d6) of **11** (ethyl acrylate and diphenylphosphine) after 24 h.

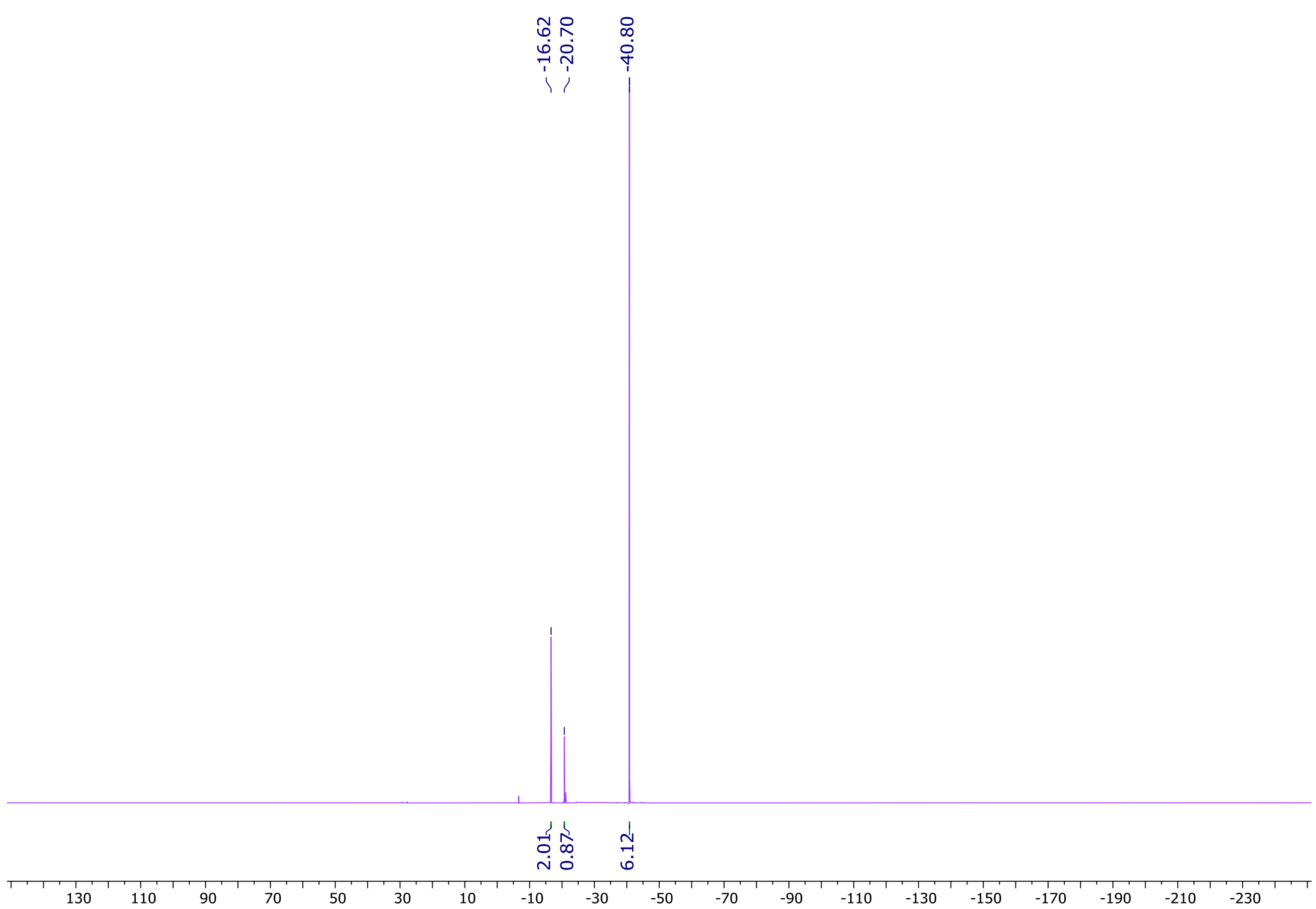

Figure S 28. $^{31}P\{^{1}H\}$ NMR spectrum (202 MHz, DMSO) of **11** (ethyl acrylate and diphenylphosphine) after 24 h.

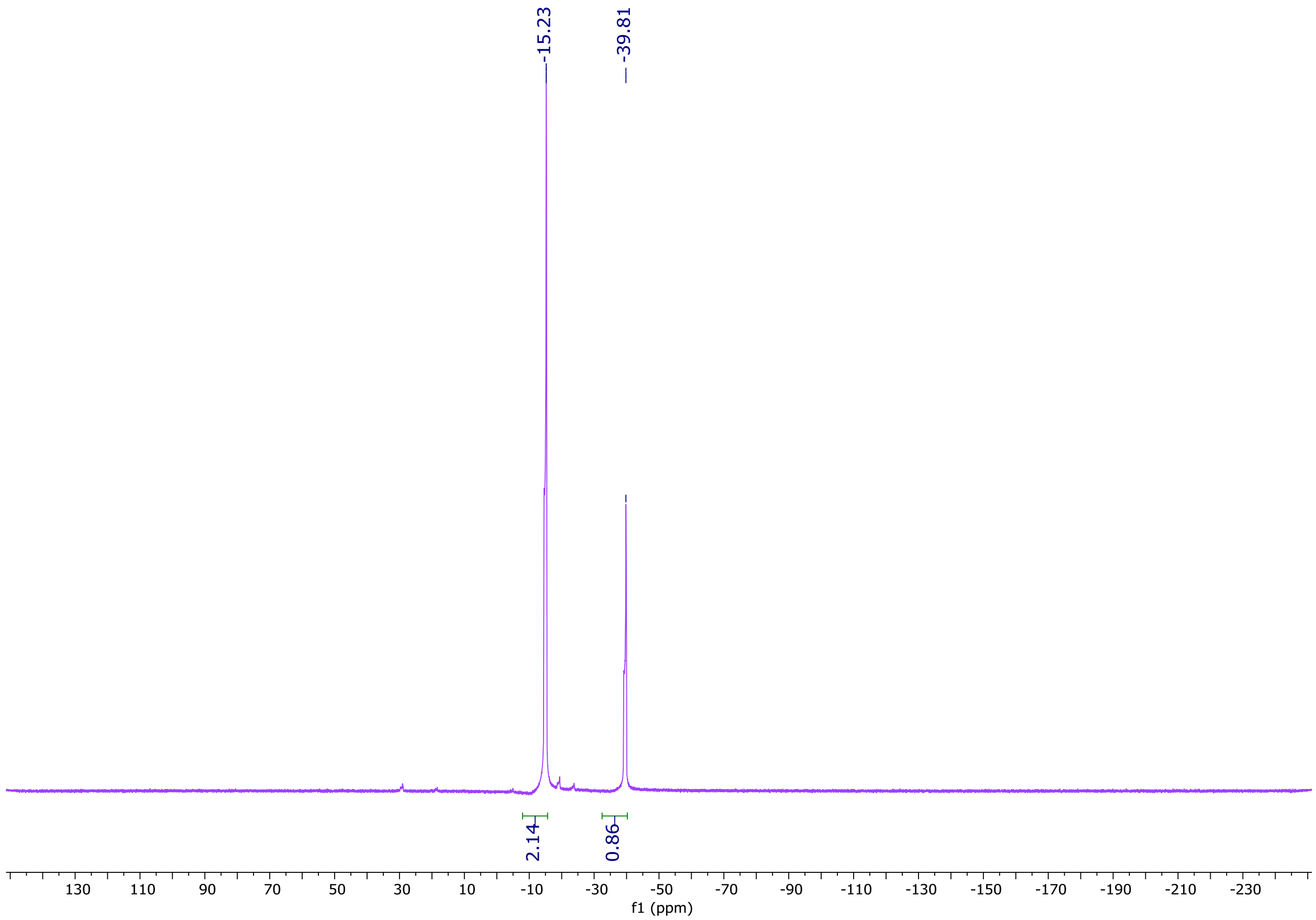

Figure S 29. $^{31}P\{^{1}H\}$ NMR spectrum (202 MHz, none) of **11** (ethyl acrylate and diphenylphosphine) after 24 h.

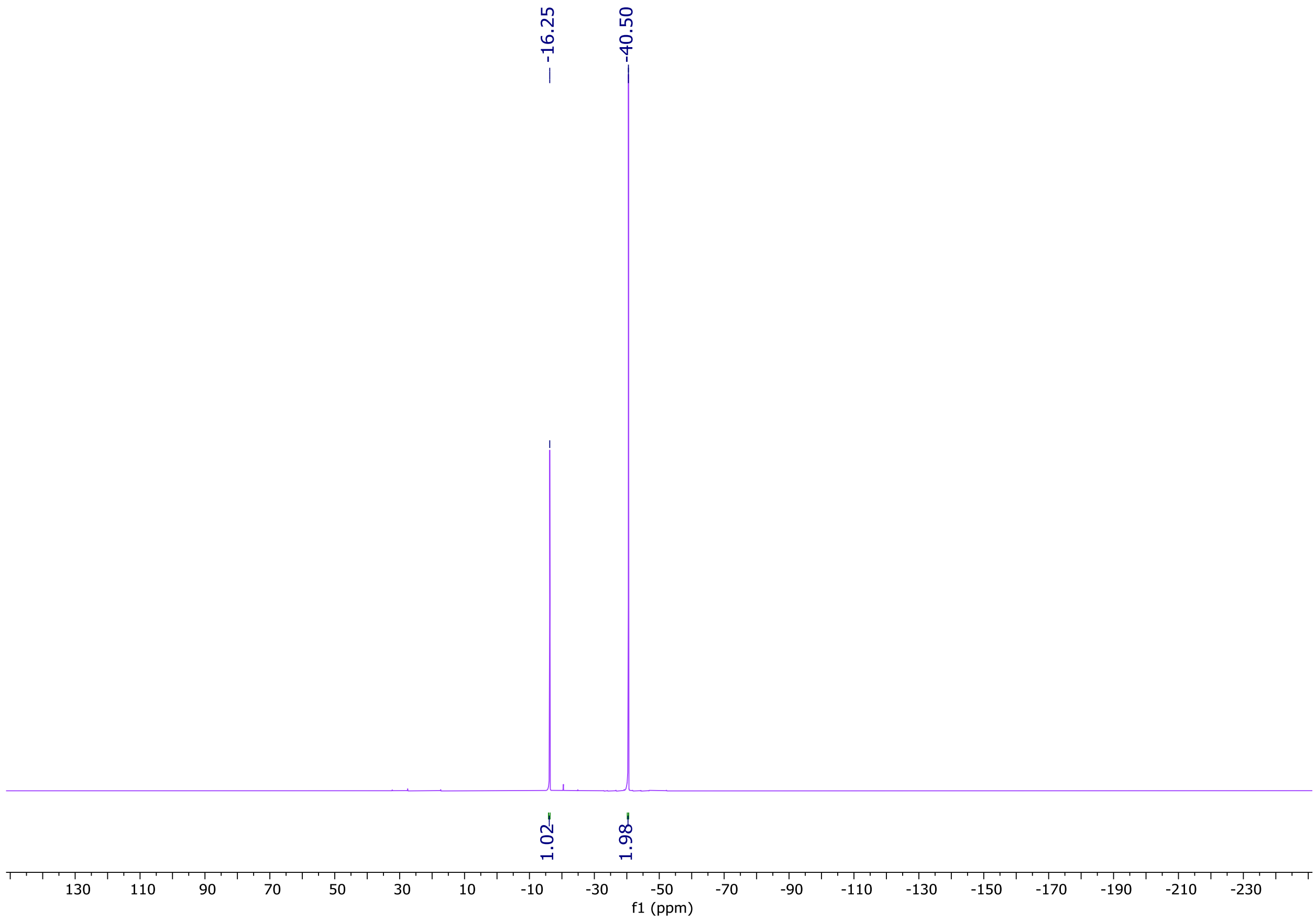

Figure S 30. $^{31}P\{^1H\}$ NMR spectrum (202 MHz, C_6H_5Br) of **11** (ethyl acrylate and diphenylphosphine) after 24 h.

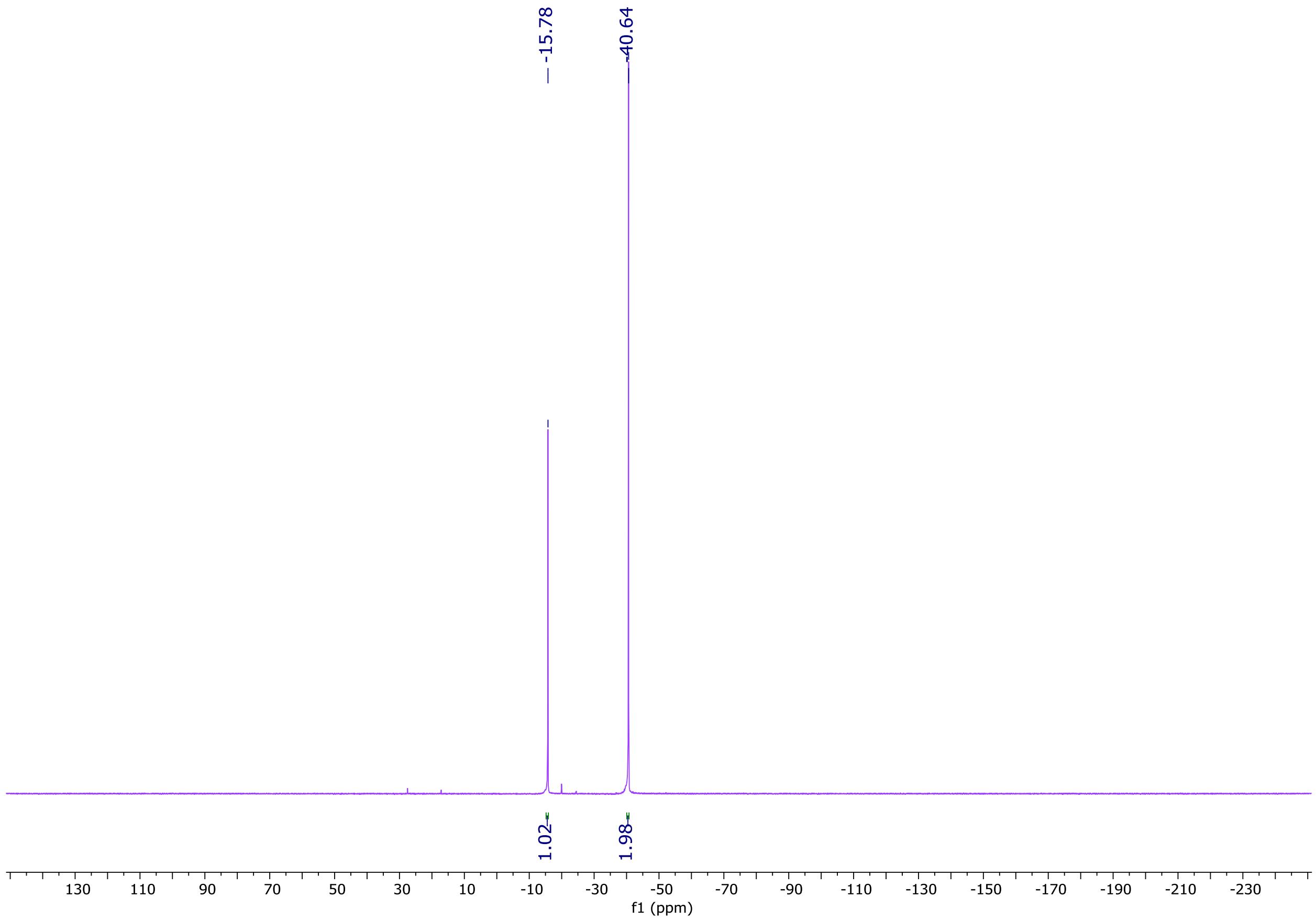

Figure S 31. $^{31}P\{^{1}H\}$ NMR spectrum (202 MHz, C_6D_6) of **11** (ethyl acrylate and diphenylphosphine) after 24 h.

Hydrophosphination of styrene in various solvents

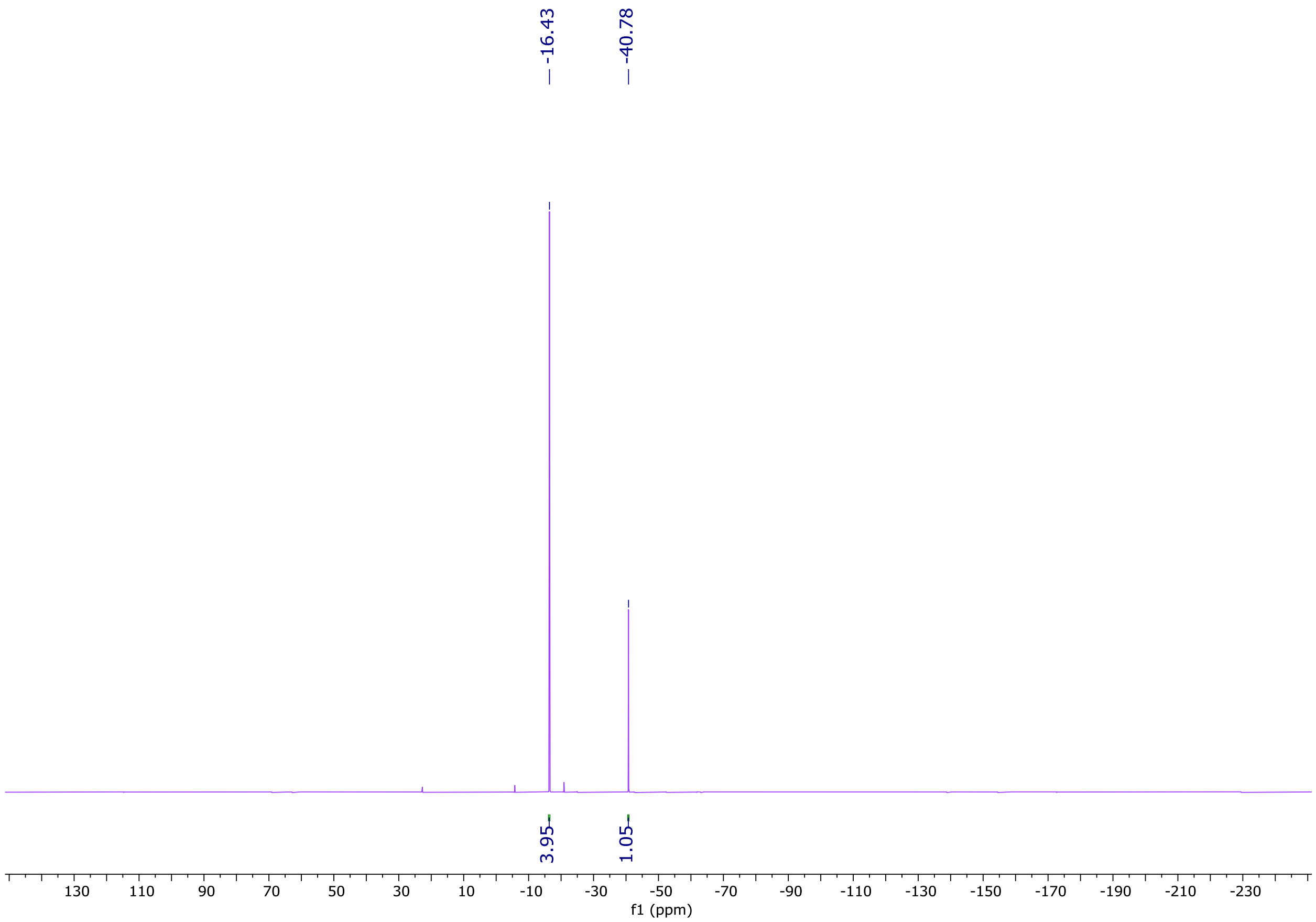

Figure S 32. $^{31}P\{^{1}H\}$ NMR spectrum (202 MHz, MeOH) of **1** (styrene and diphenylphosphine) after 24 h

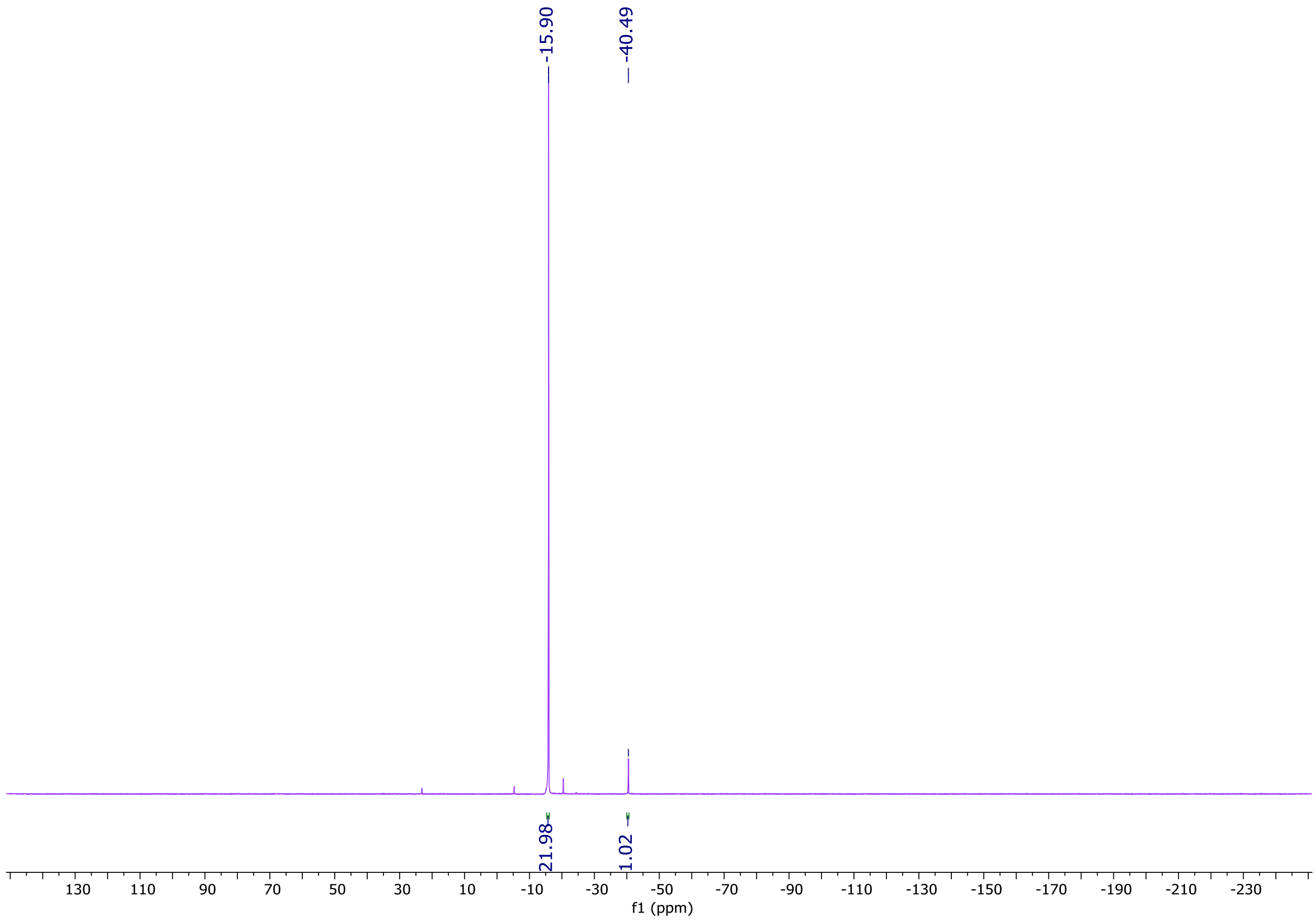

Figure S 33. $^{31}P\{^{1}H\}$ NMR spectrum (202 MHz, EtOH) of **1** (styrene and diphenylphosphine) after 24 h.

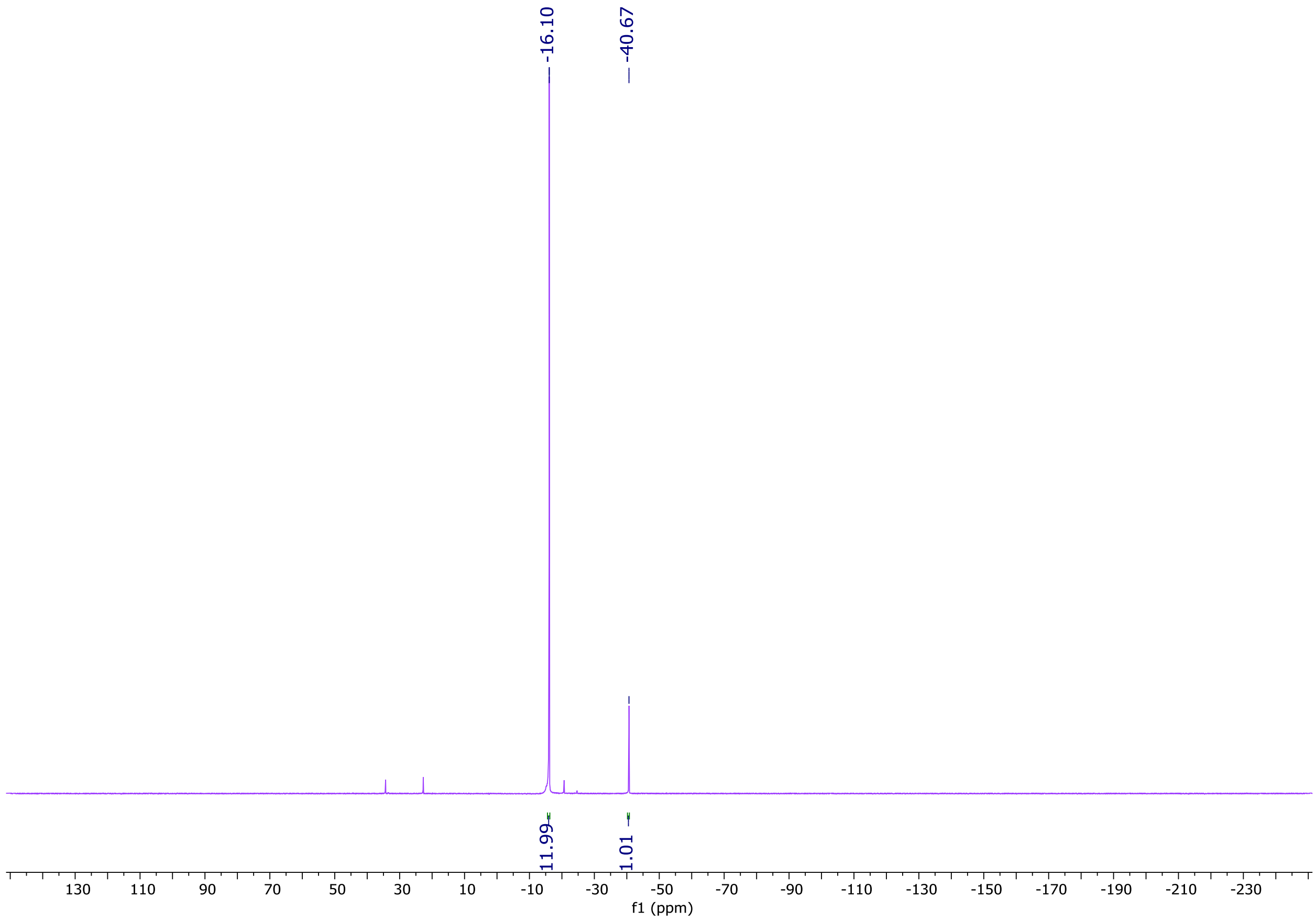

Figure S 34. $^{31}P\{^1H\}$ NMR spectrum (202 MHz, n-PrOH) of **1** (styrene and diphenylphosphine) after 24 h.

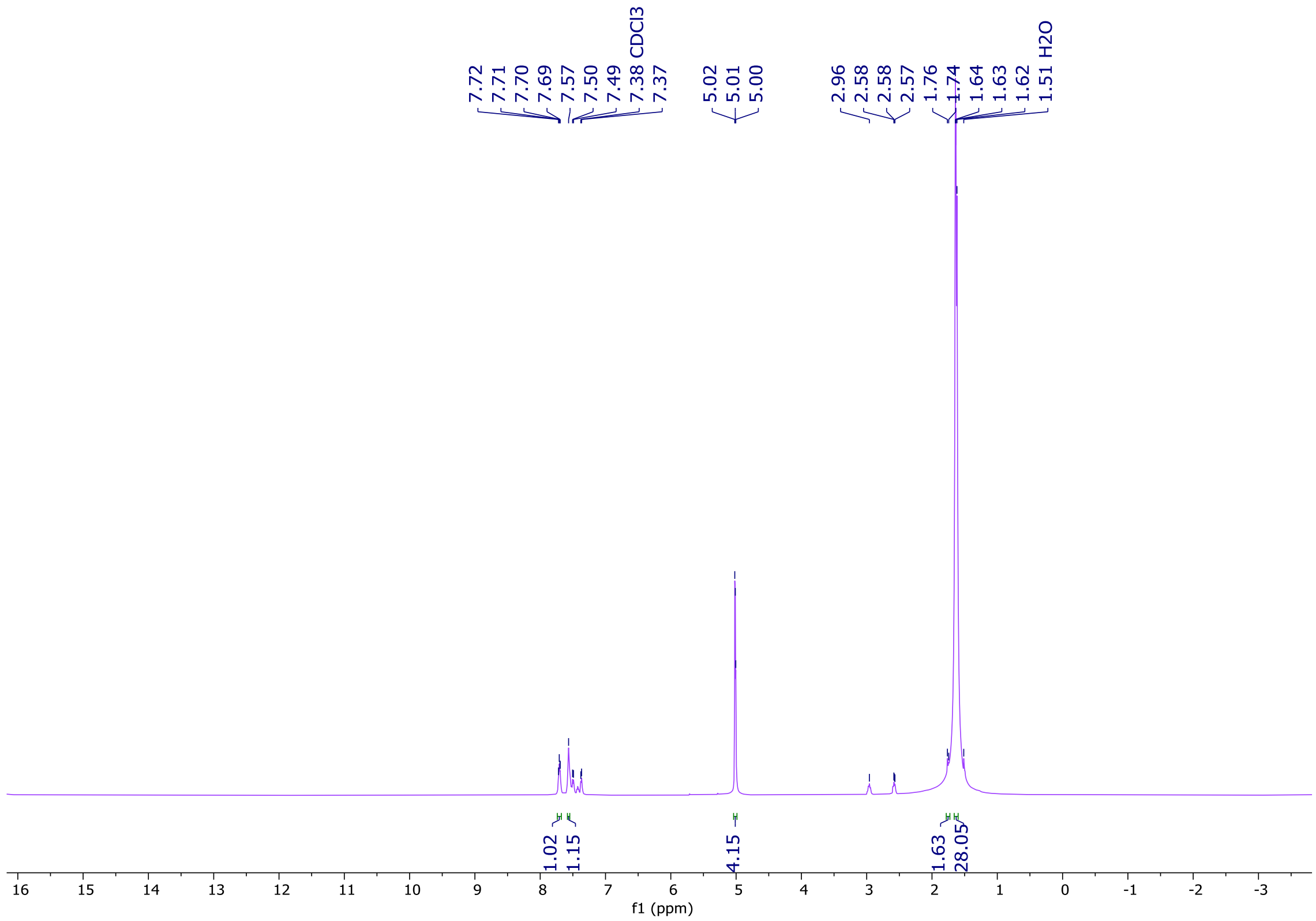

Figure S 35. ^{1}H NMR spectrum (500 MHz, n-BuOH) of **1** (styrene and diphenylphosphine) after 24 h.

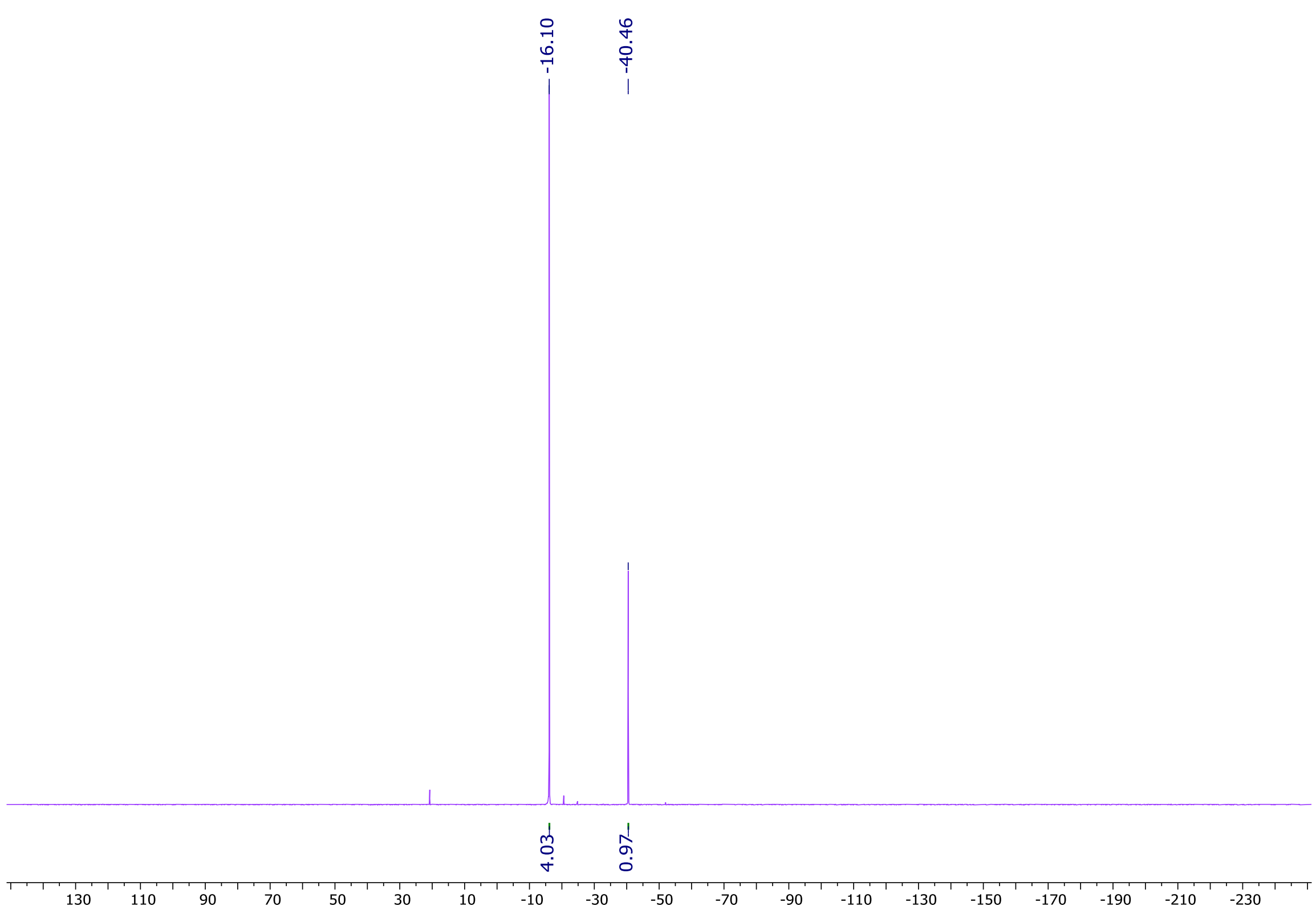

Figure S 36. $^{31}P\{^{1}H\}$ NMR spectrum (202 MHz, n-BuOH) of **1** (styrene and diphenylphosphine) after 24 h.

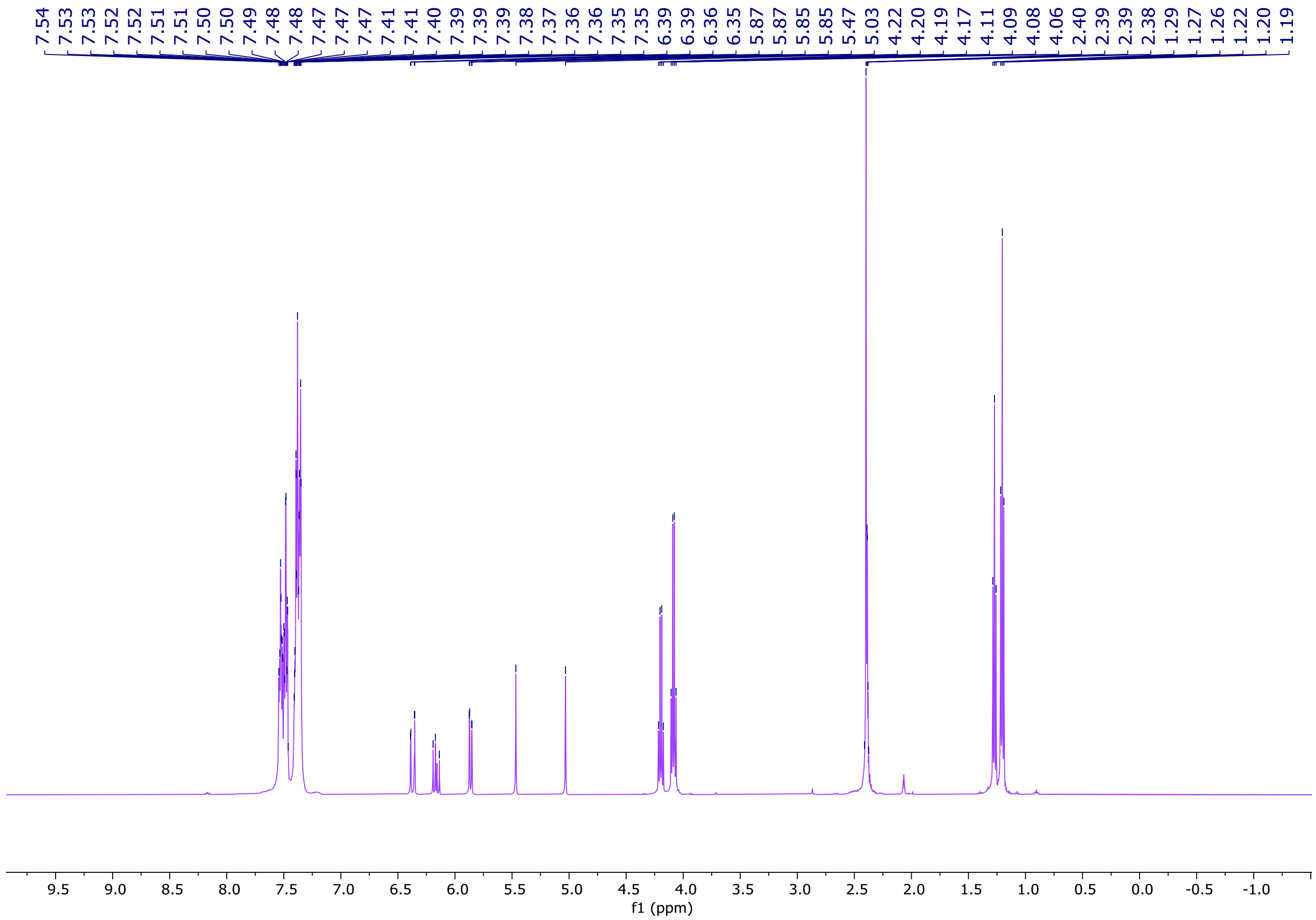

Figure S 37. ^{1}H NMR spectrum (500 MHz, Acetone-d_6) of **1** (styrene and diphenylphosphine) after 24 h.

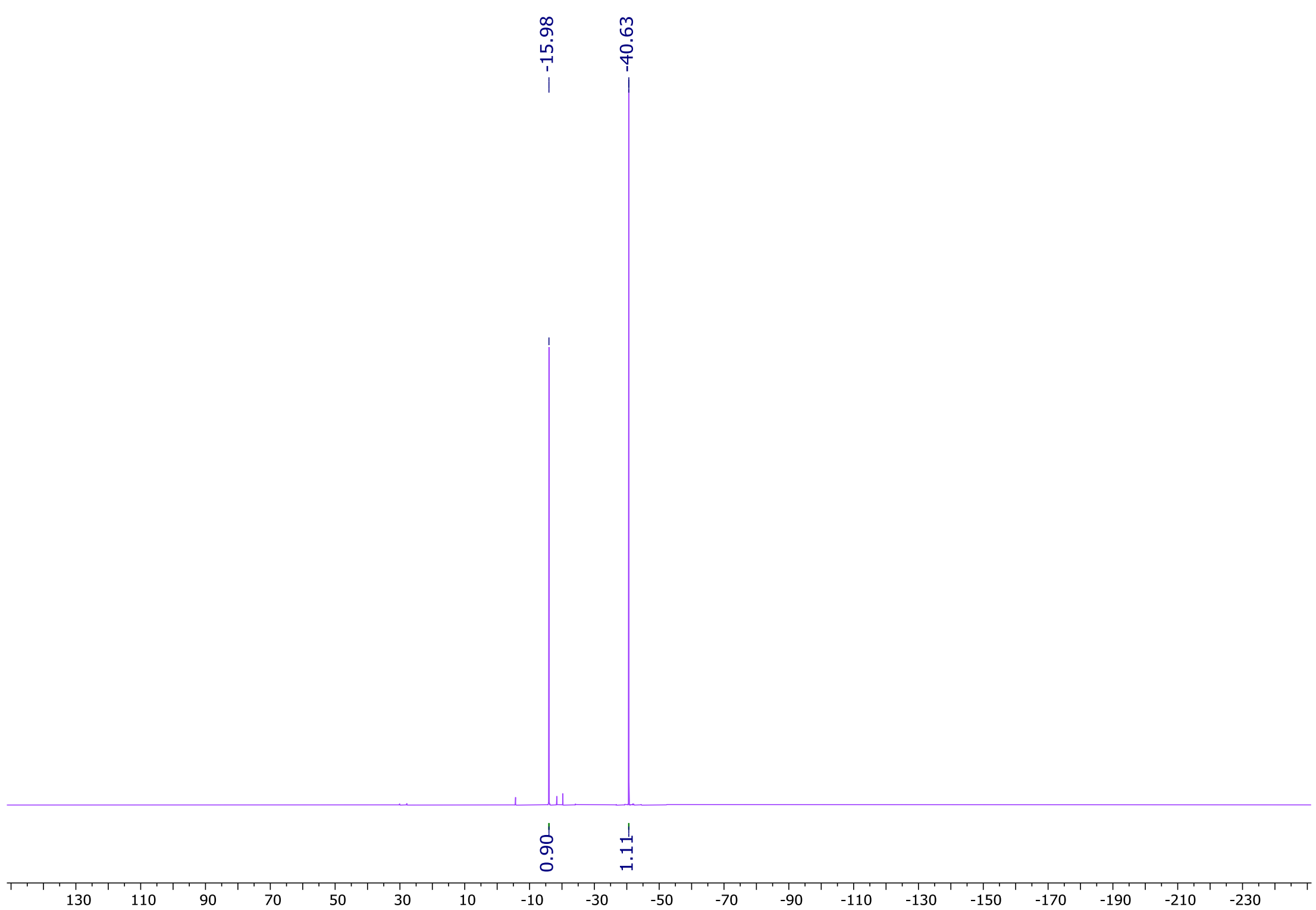

Figure S 38. $^{31}P\{^{1}H\}$ NMR spectrum (202 MHz, Acetone) of **1** (styrene and diphenylphosphine) after 24 h.

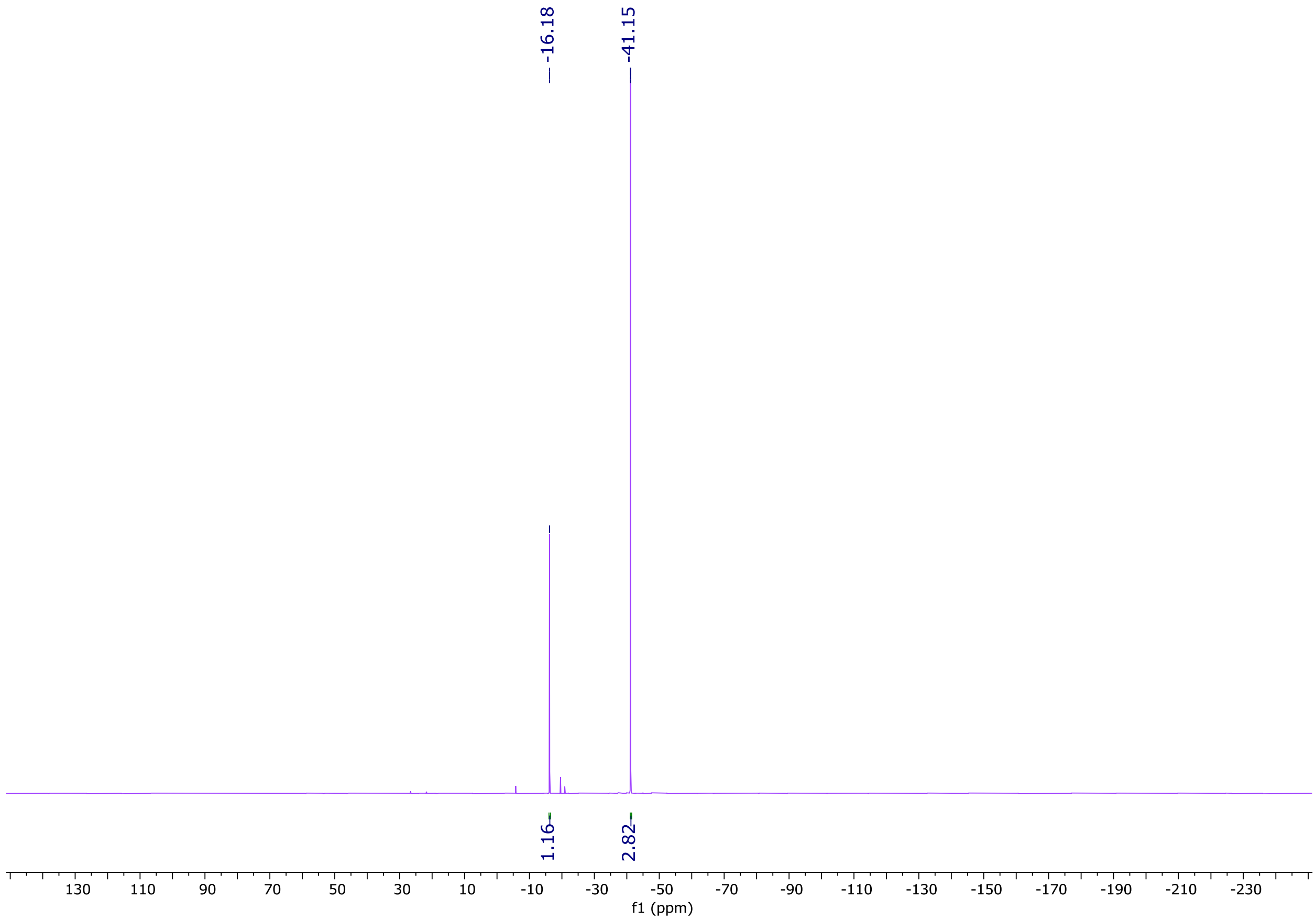

Figure S 39. $^{31}P\{^{1}H\}$ NMR spectrum (202 MHz, DMF) of **1** (styrene and diphenylphosphine) after 24 h.

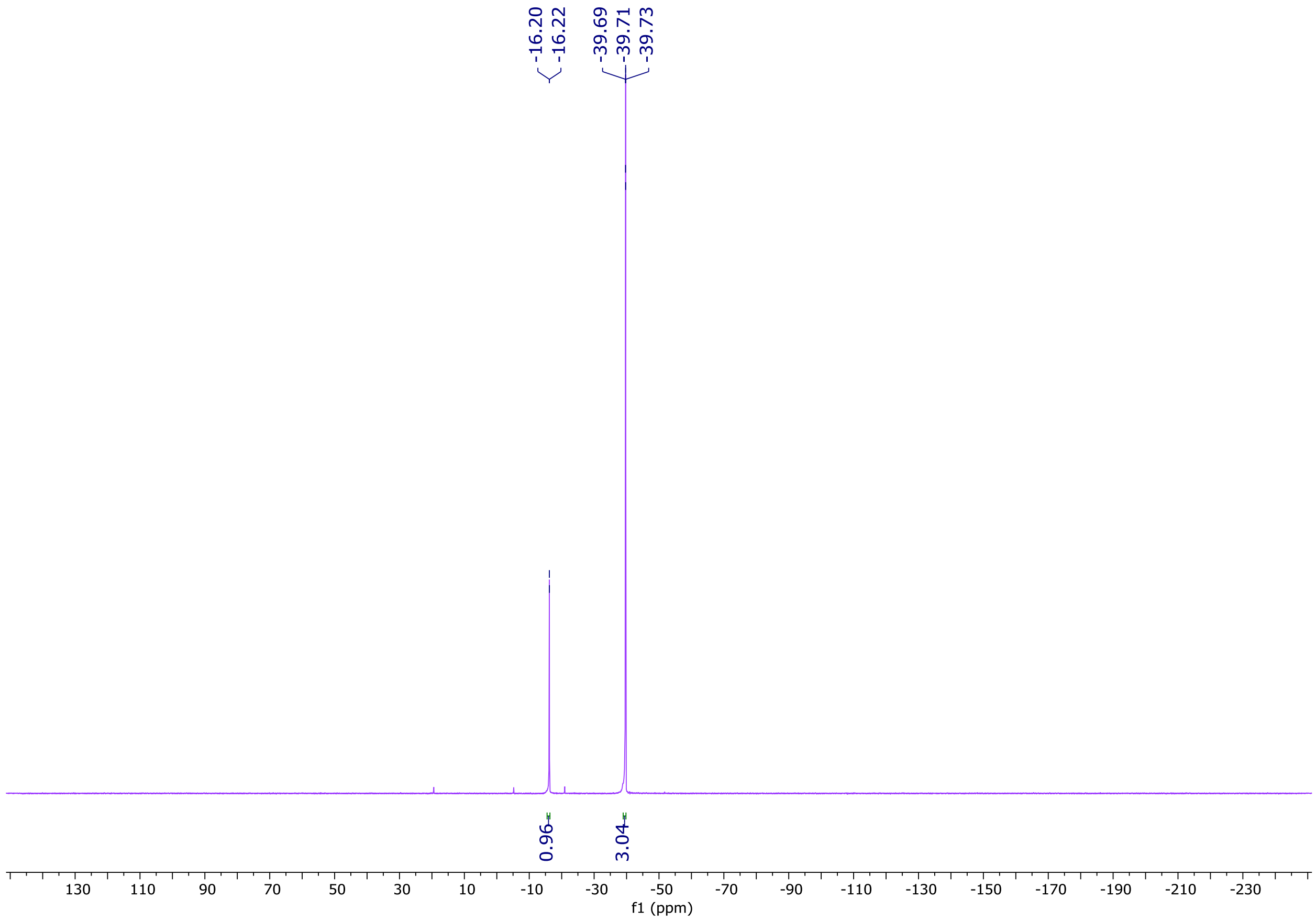

Figure S 40. $^{31}P\{^1H\}$ NMR spectrum (202 MHz, MeCN) of **1** (styrene and diphenylphosphine) after 24 h.

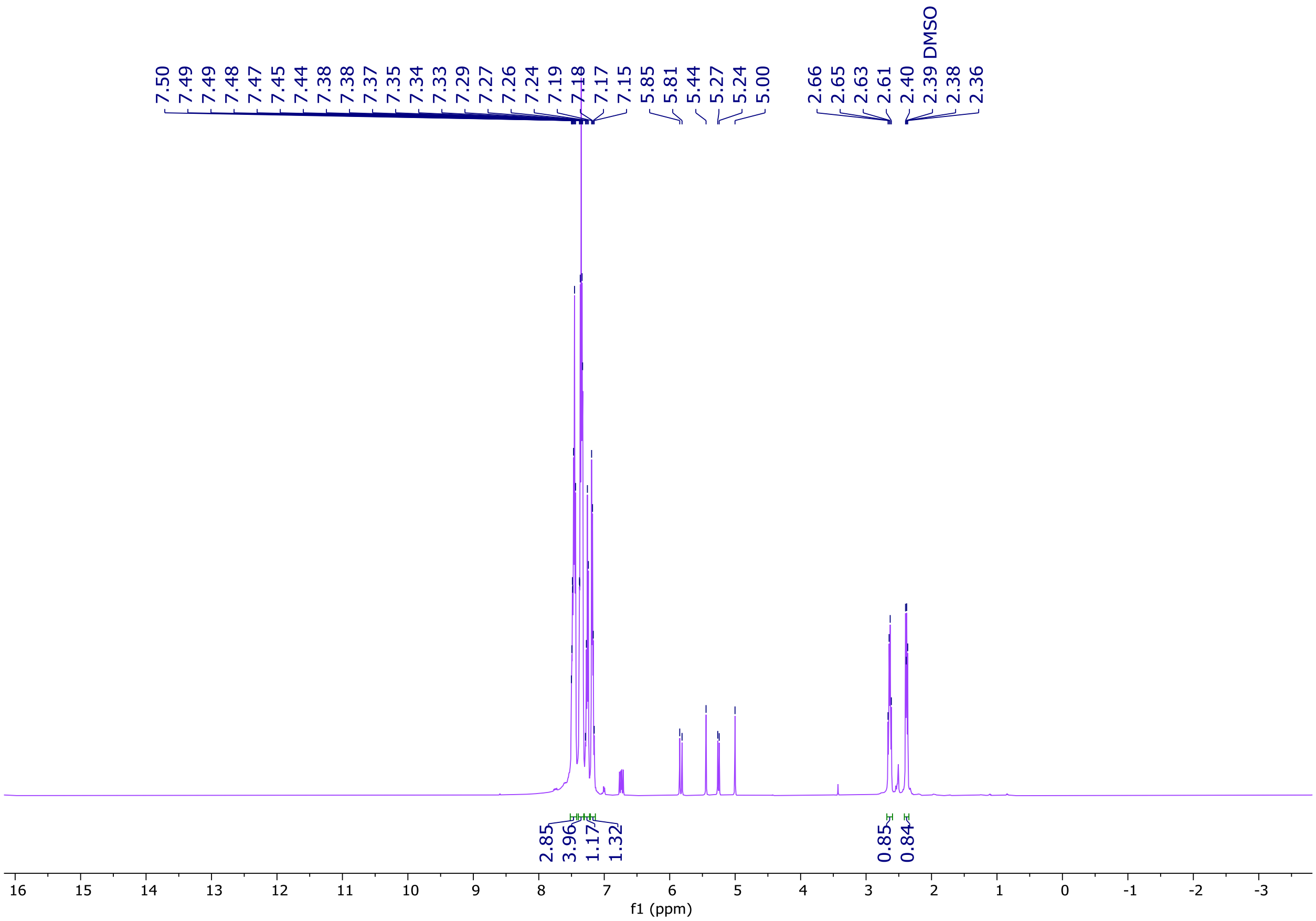

Figure S 41. ^{1}H NMR spectrum (500 MHz, DMSO-d_6) of **1** (styrene and diphenylphosphine) after 24 h.

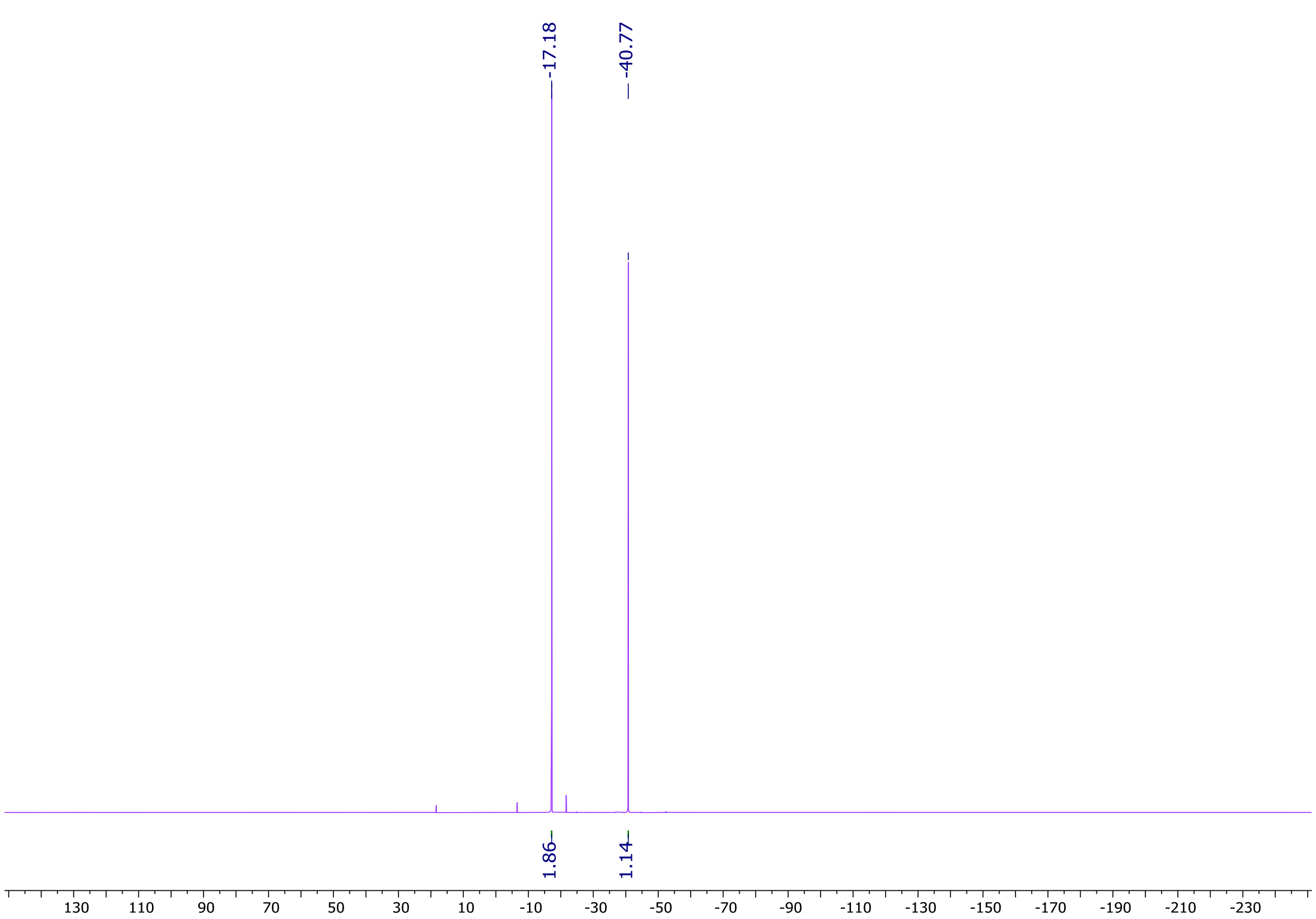

Figure S 42. $^{31}P\{^{1}H\}$ NMR spectrum (202 MHz, DMSO) of **1** (styrene and diphenylphosphine) after 24 h.

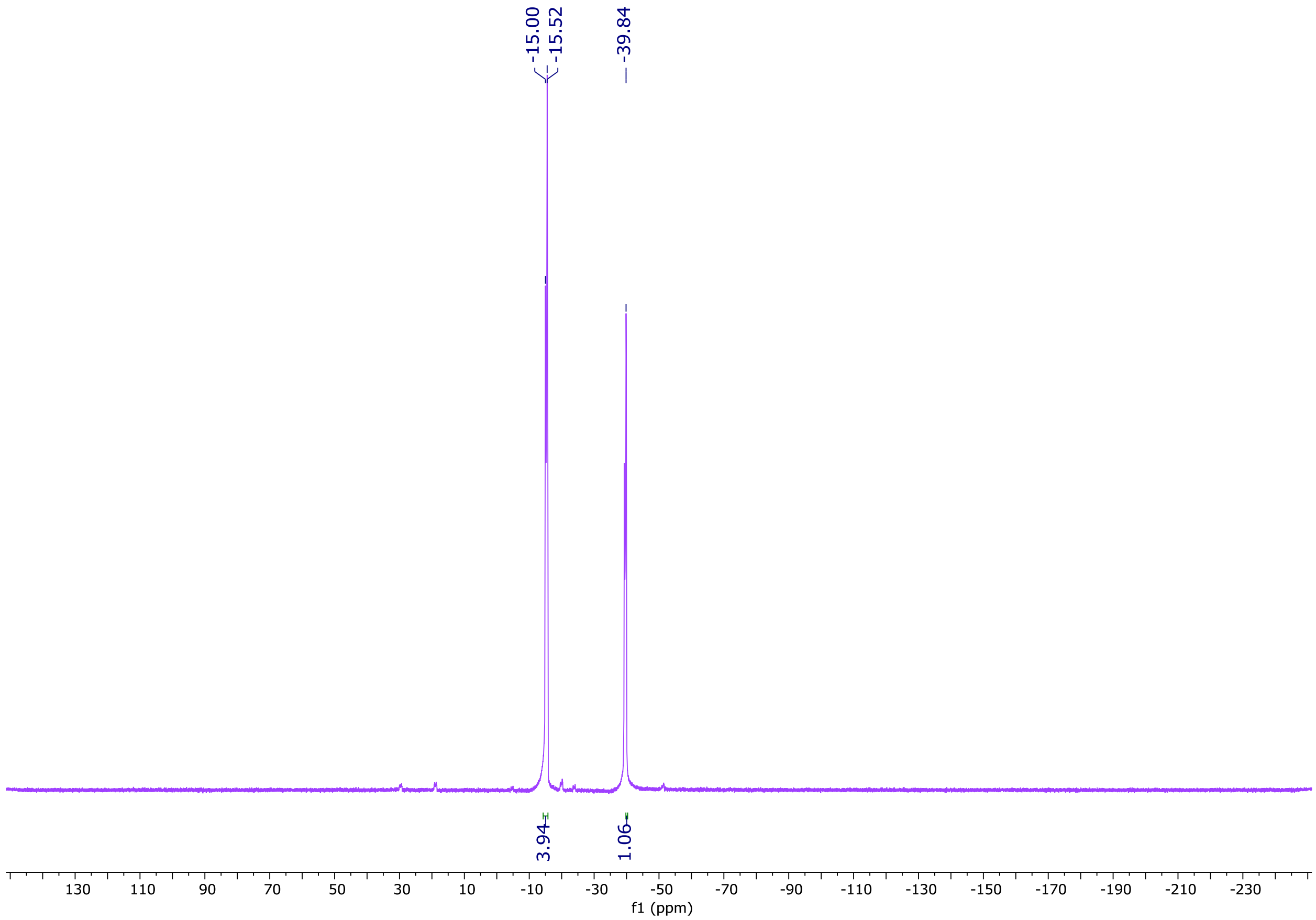

Figure S 43. $^{31}P\{^{1}H\}$ NMR spectrum (202 MHz, none) of **1** (styrene and diphenylphosphine) after 24 h.

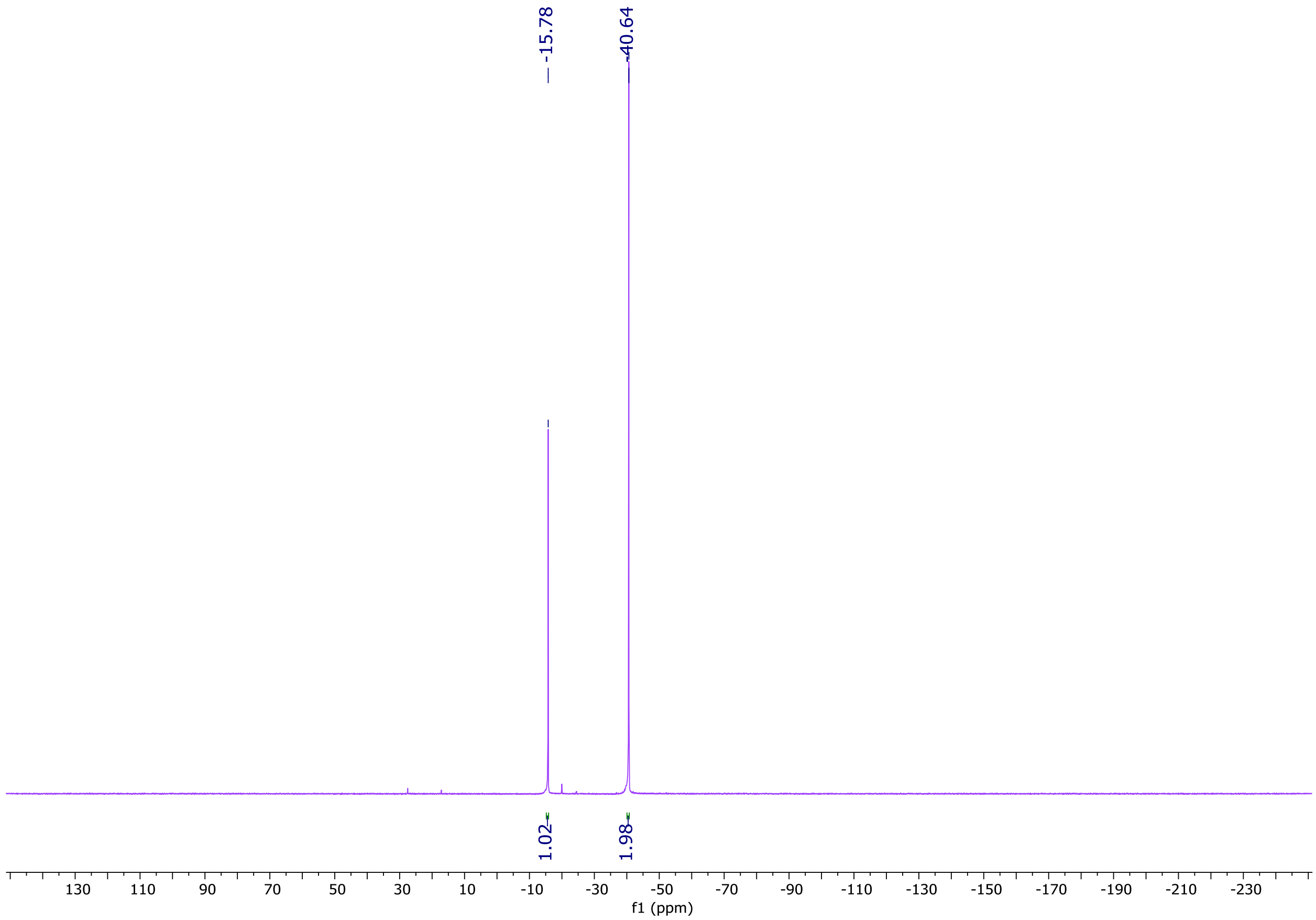

Figure S 44. $^{31}P\{^{1}H\}$ NMR spectrum (202 MHz, C_6H_6) of **1** (styrene and diphenylphosphine) after 24 h.

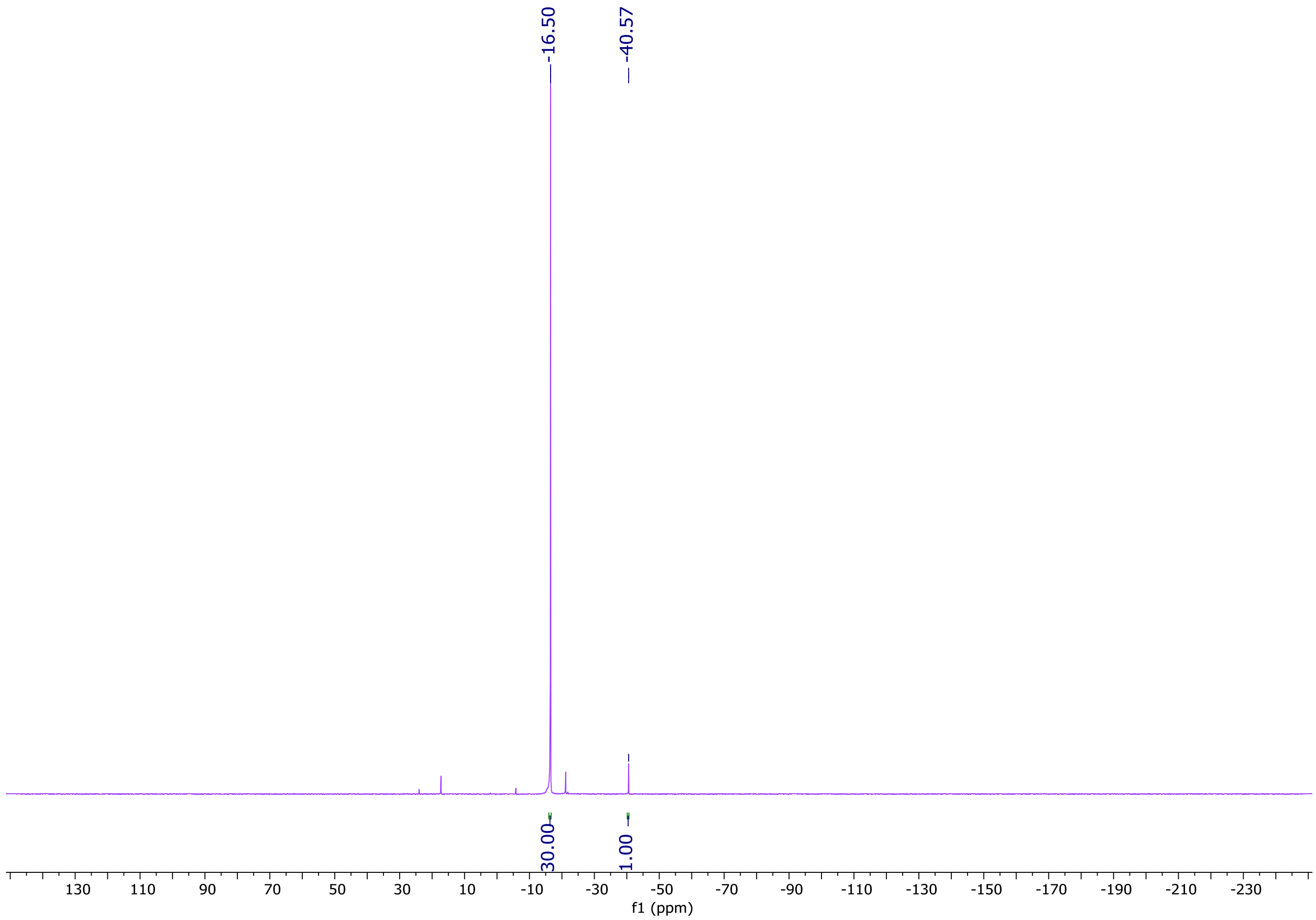

Figure S 45. $^{31}P\{^1H\}$ NMR spectrum (202 MHz, C_6H_5Br) of **1** (styrene and diphenylphosphine) after 24 h.

EtOH-mediated hydrophosphination of alkenes

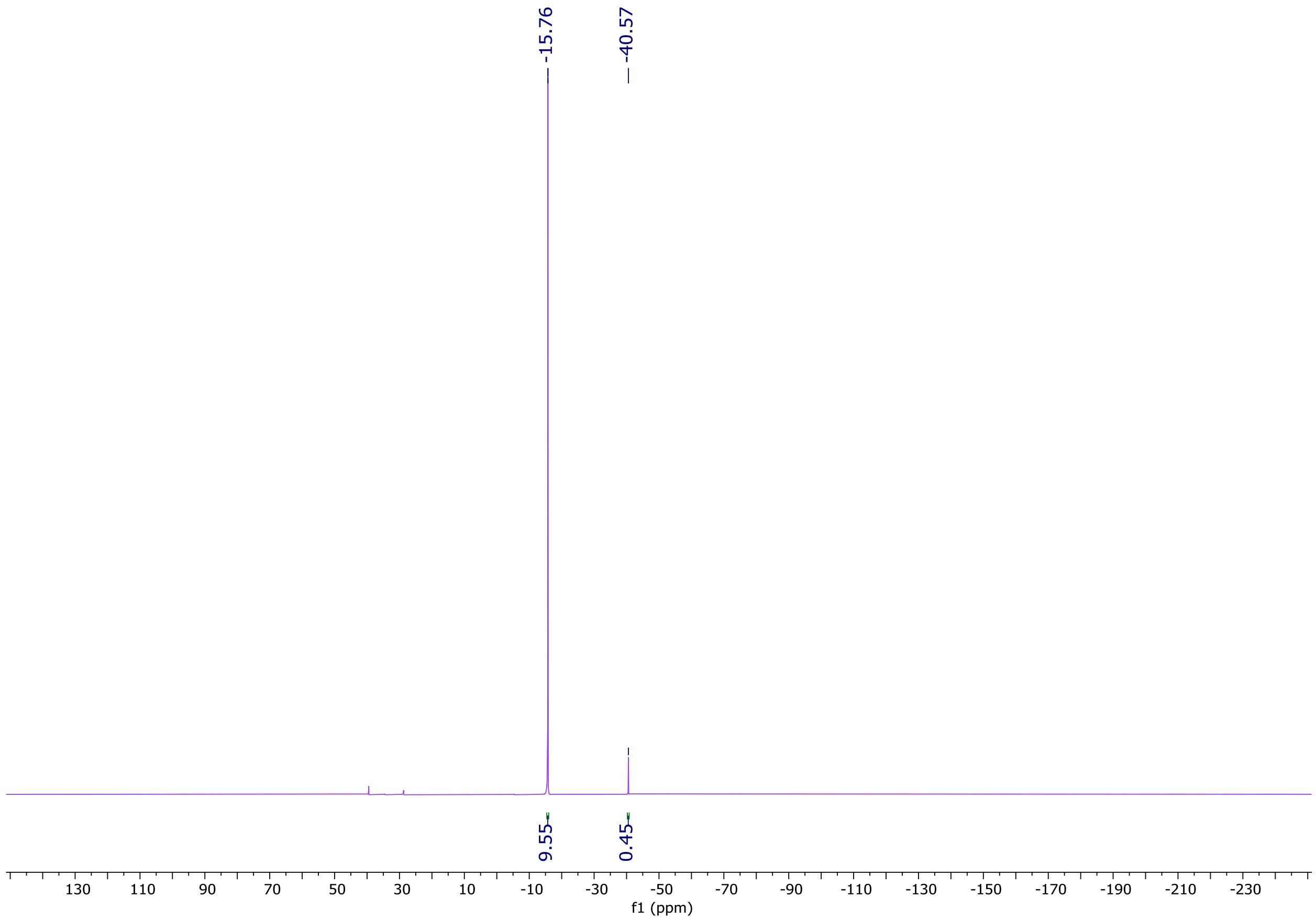

Figure S 46. $^{31}P\{^{1}H\}$ NMR spectrum (202 MHz, EtOH) of **11** (ethylacrylate and Ph_2PH).

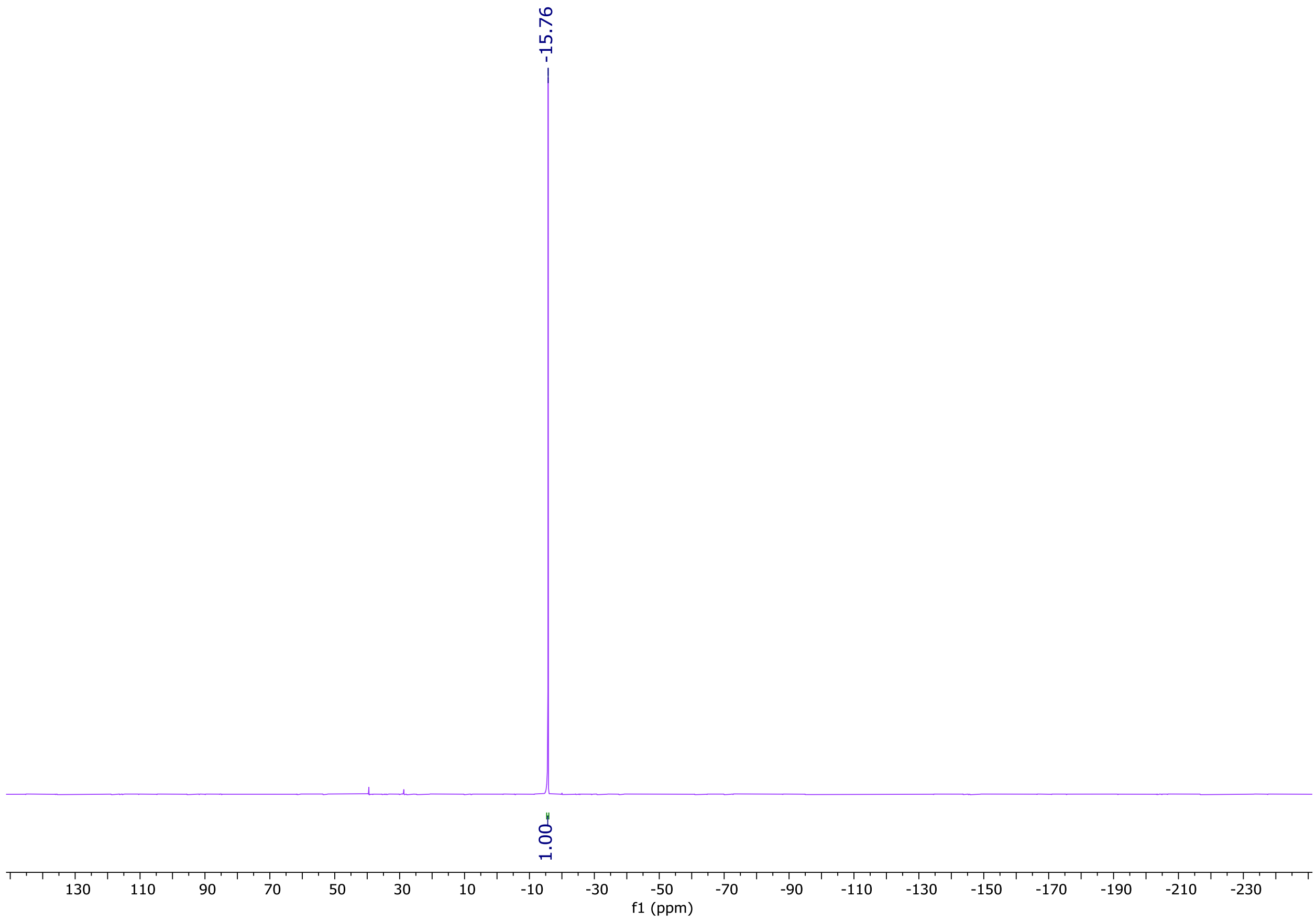

Figure S 47. $^{31}P\{^1H\}$ NMR spectrum (202 MHz, EtOH) of **12** (methylacrylate and Ph_2PH).

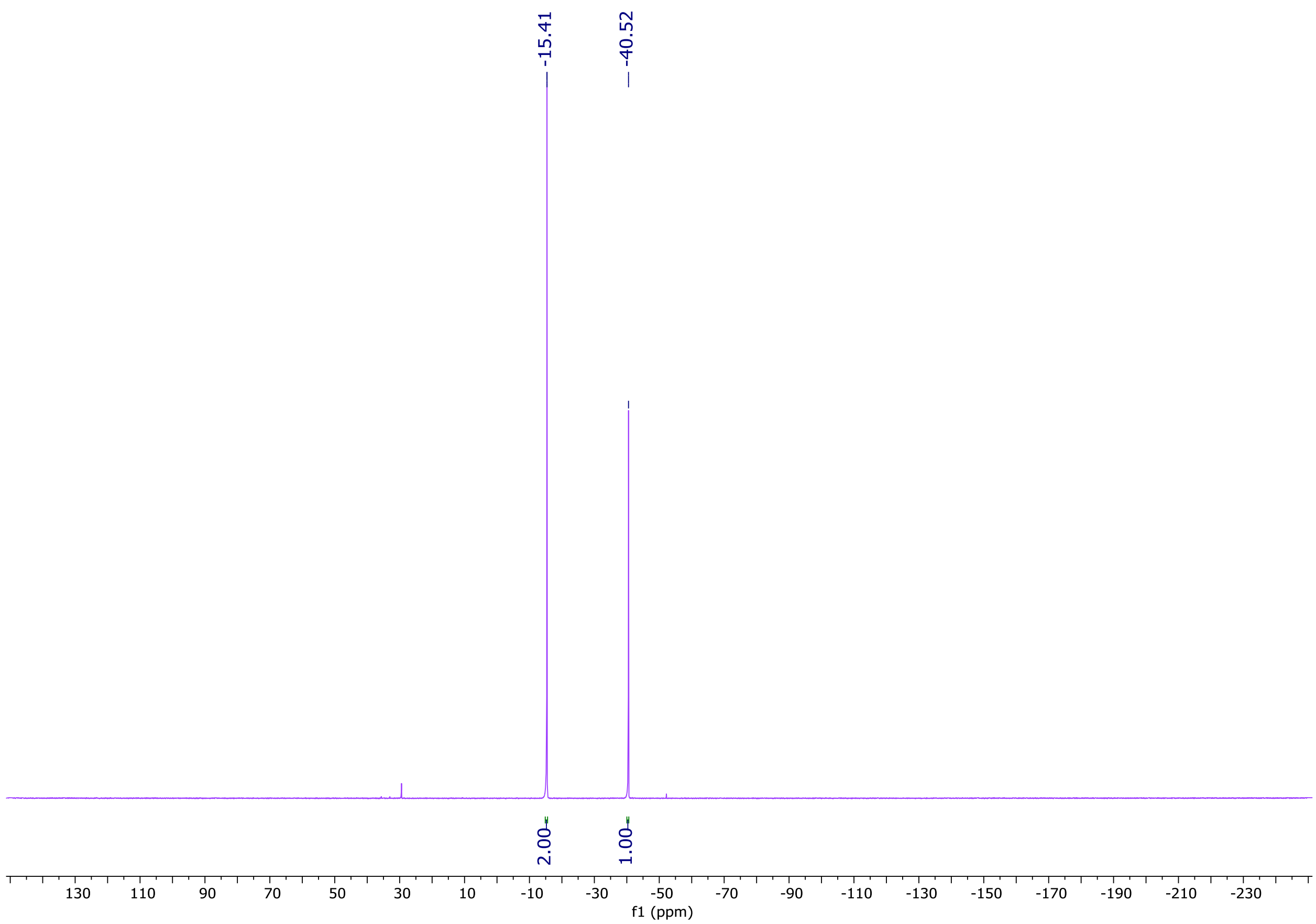

Figure S 48. $^{31}P\{^{1}H\}$ NMR spectrum (202 MHz, EtOH) of **14** (acrylonitrile and Ph_2PH).

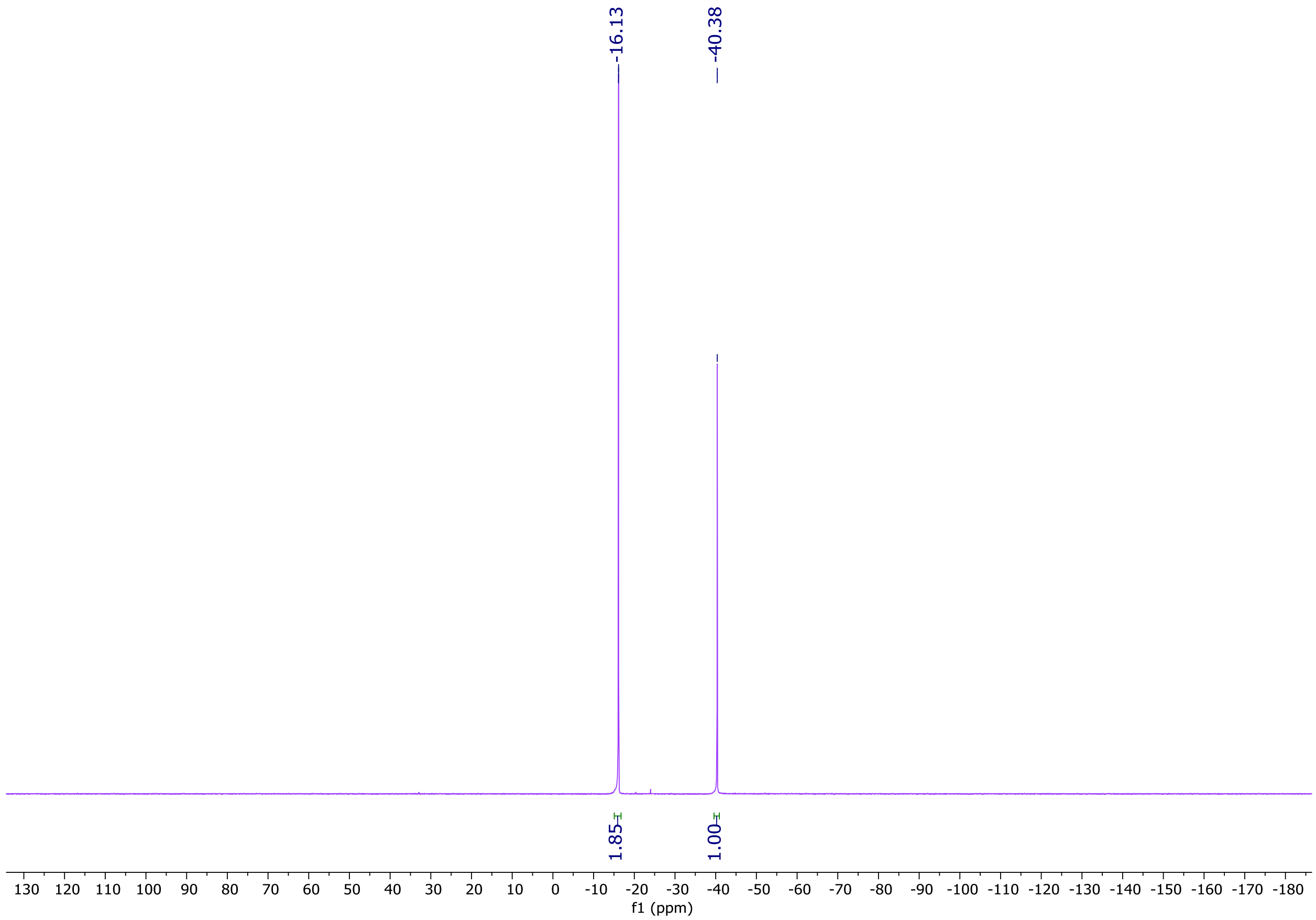

Figure S 49. $^{31}P\{^{1}H\}$ NMR spectrum (202 MHz, EtOH) of **13** (acrylonitrile and Ph_2PH).

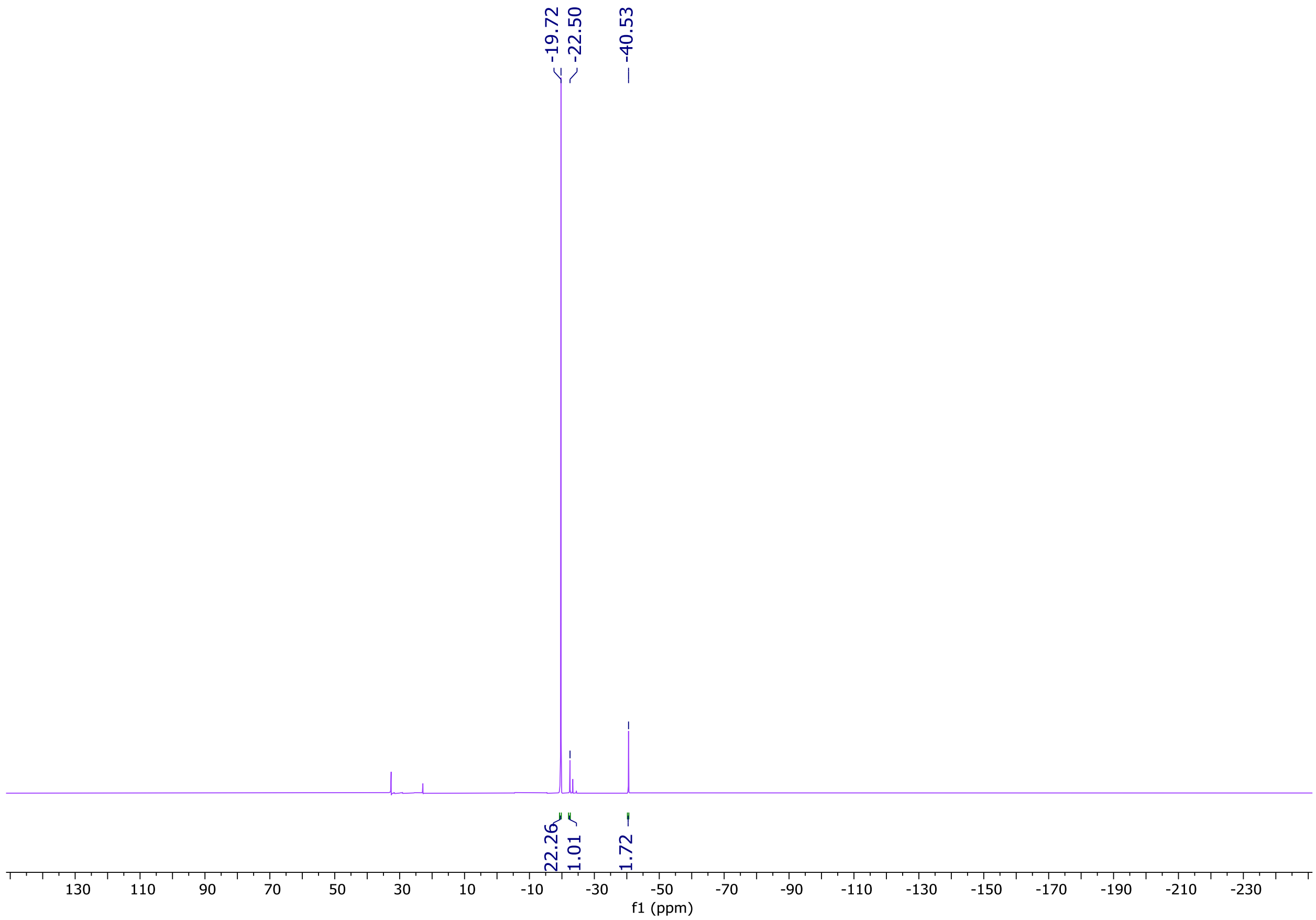

Figure S 50. $^{31}P\{^{1}H\}$ NMR spectrum (202 MHz, EtOH) of **15** (methylmethacrylate and Ph_2PH).

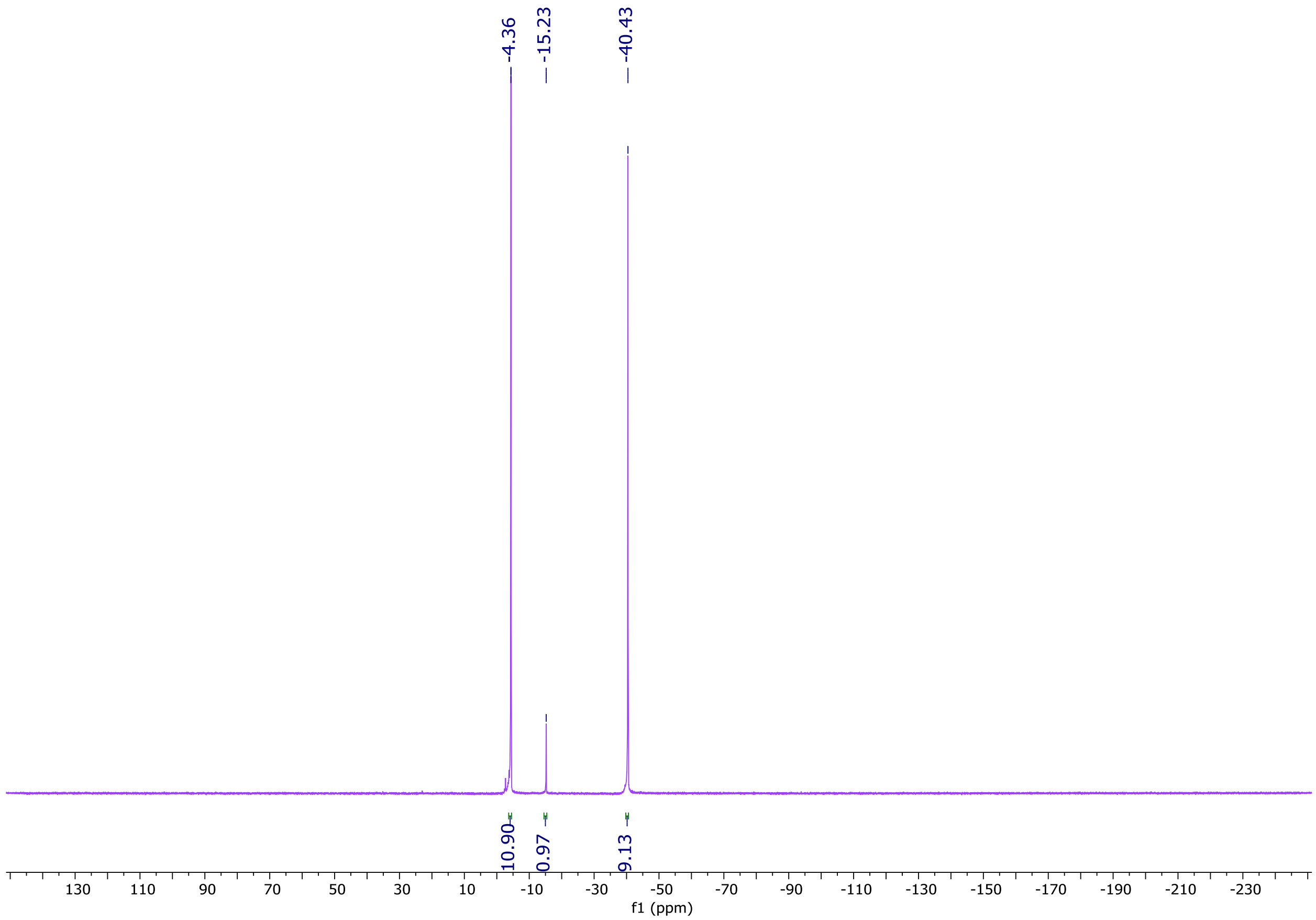

Figure S 51. $^{31}P\{^1H\}$ NMR spectrum (202 MHz, EtOH) of **16** (cyclohexanone and Ph_2PH).

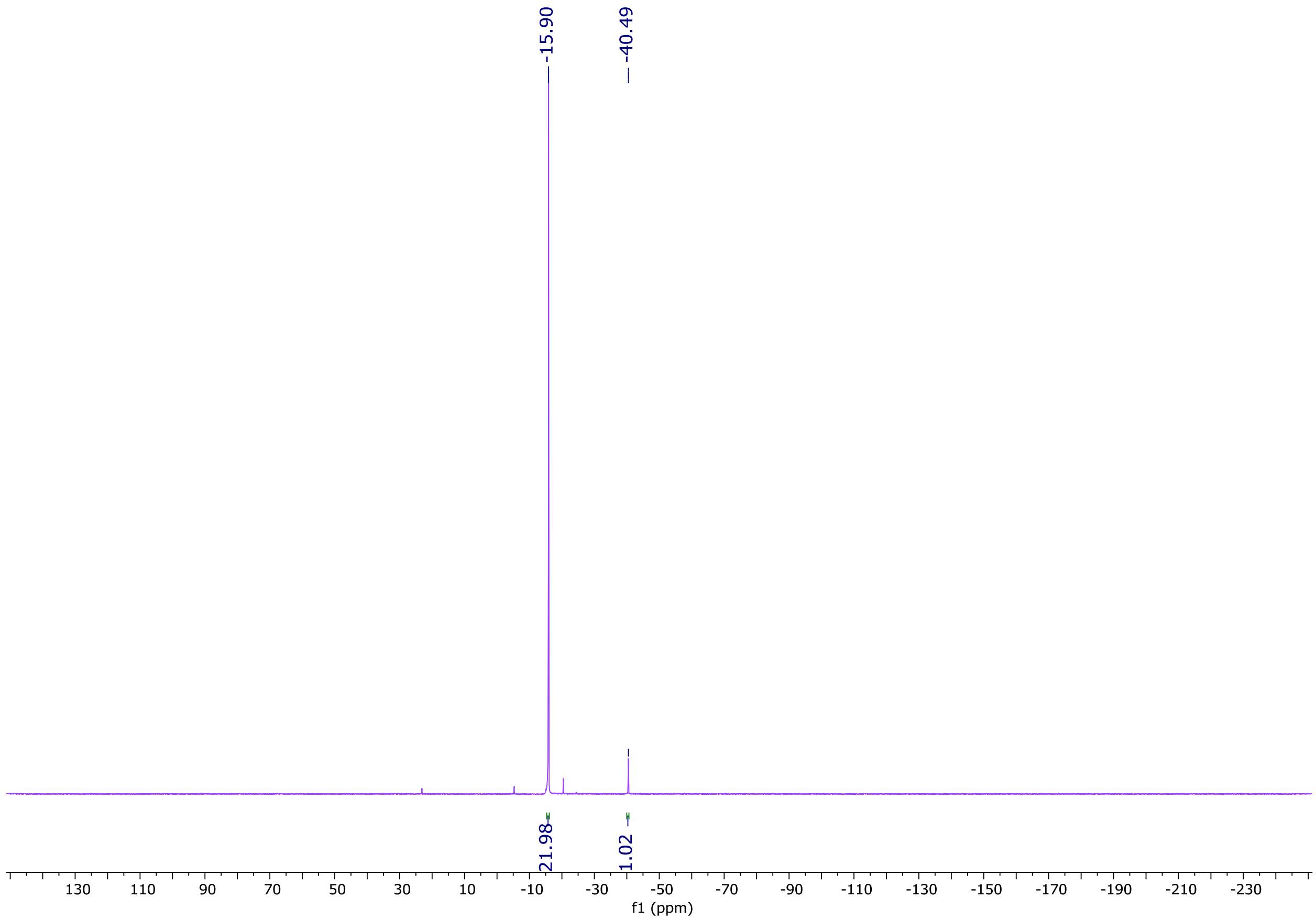

Figure S 52. $^{31}P\{^{1}H\}$ NMR spectrum (202 MHz, EtOH) of **1** (styrene and Ph_2PH).

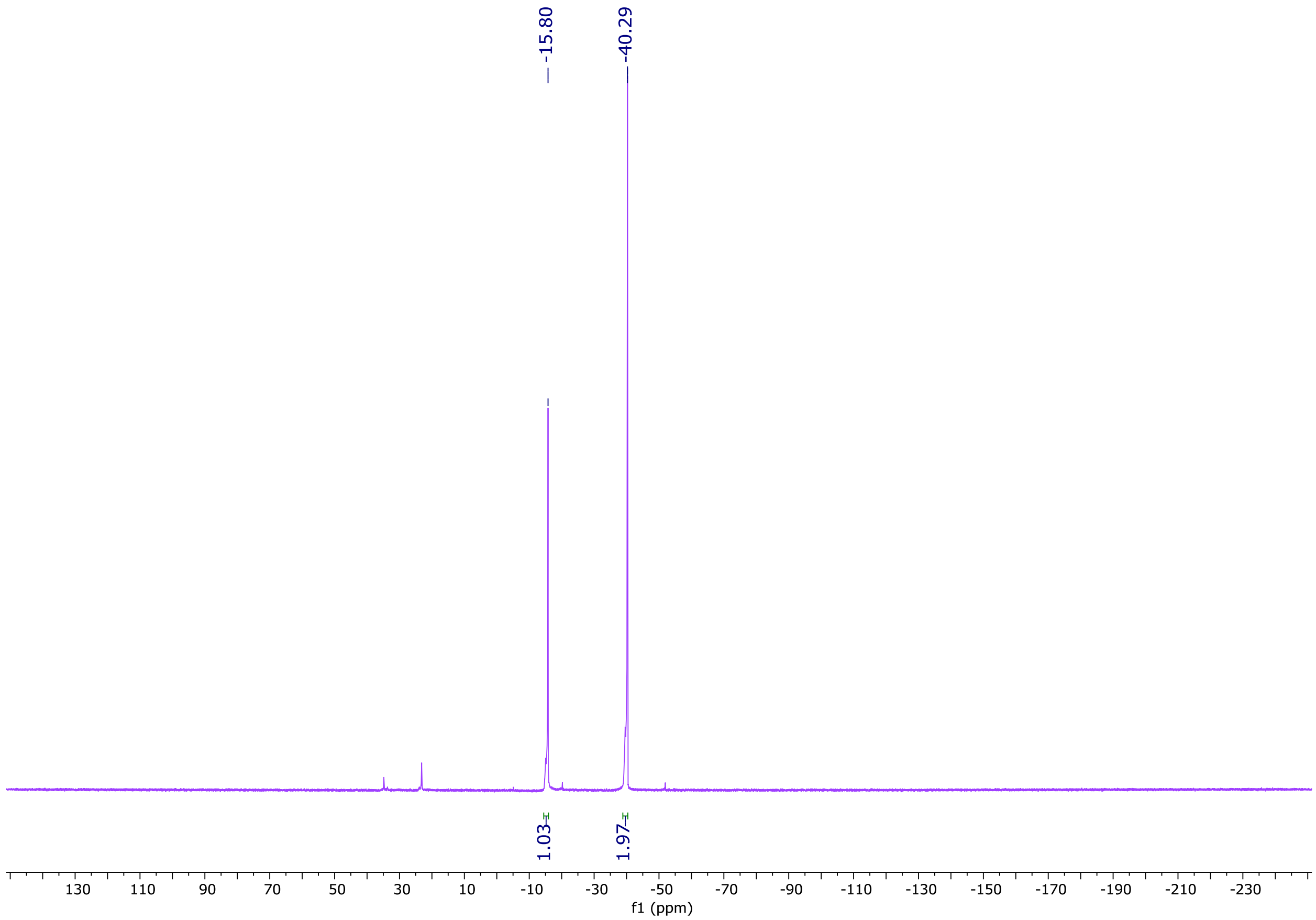

Figure S 53. $^{31}P\{^{1}H\}$ NMR spectrum (202 MHz, EtOH) of **2** (p-methylstyrene and Ph_2PH).

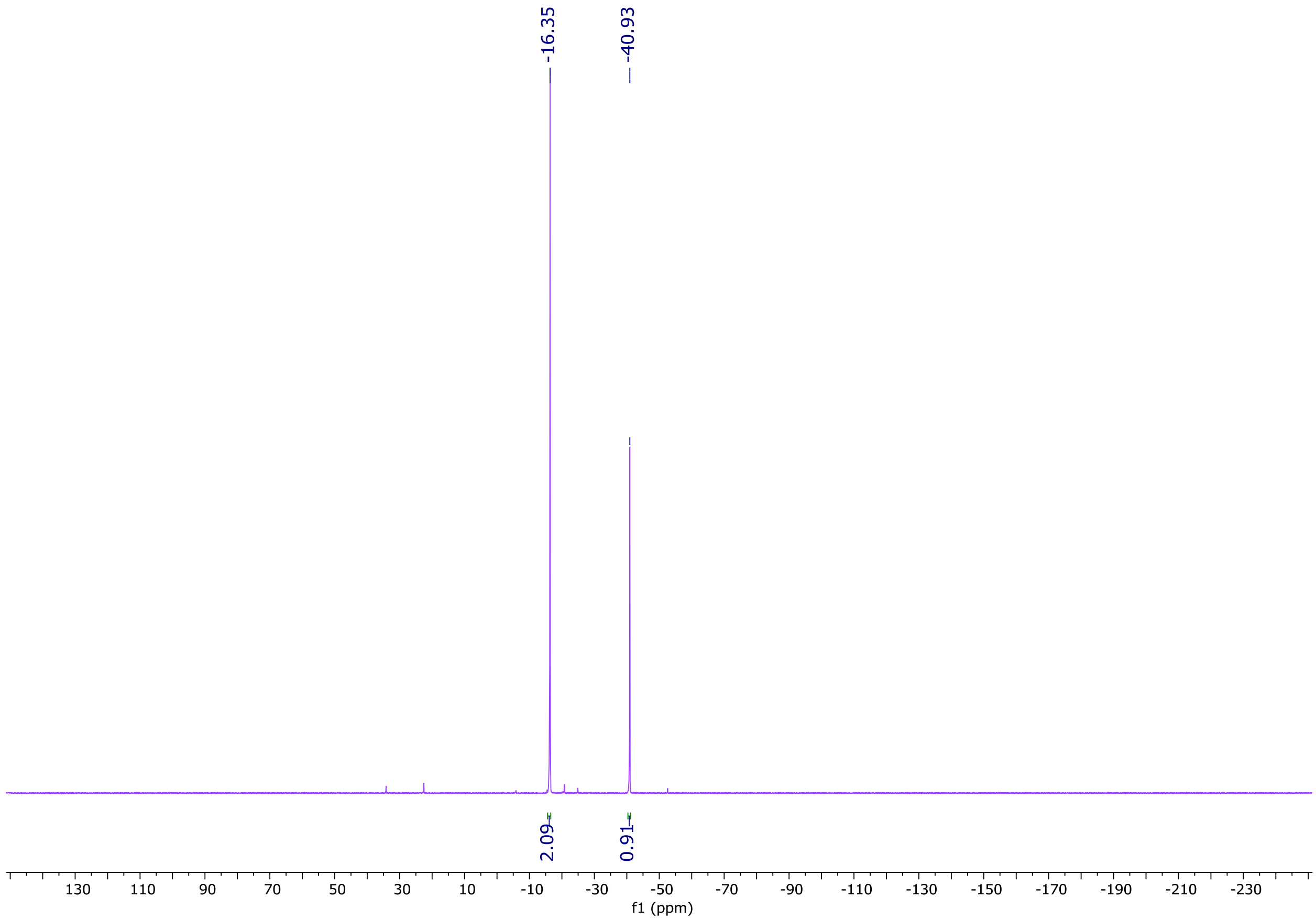

Figure S 54. $^{31}P\{^{1}H\}$ NMR spectrum (202 MHz, EtOH) of **3** (p-*tBu*styrene and Ph_2PH).

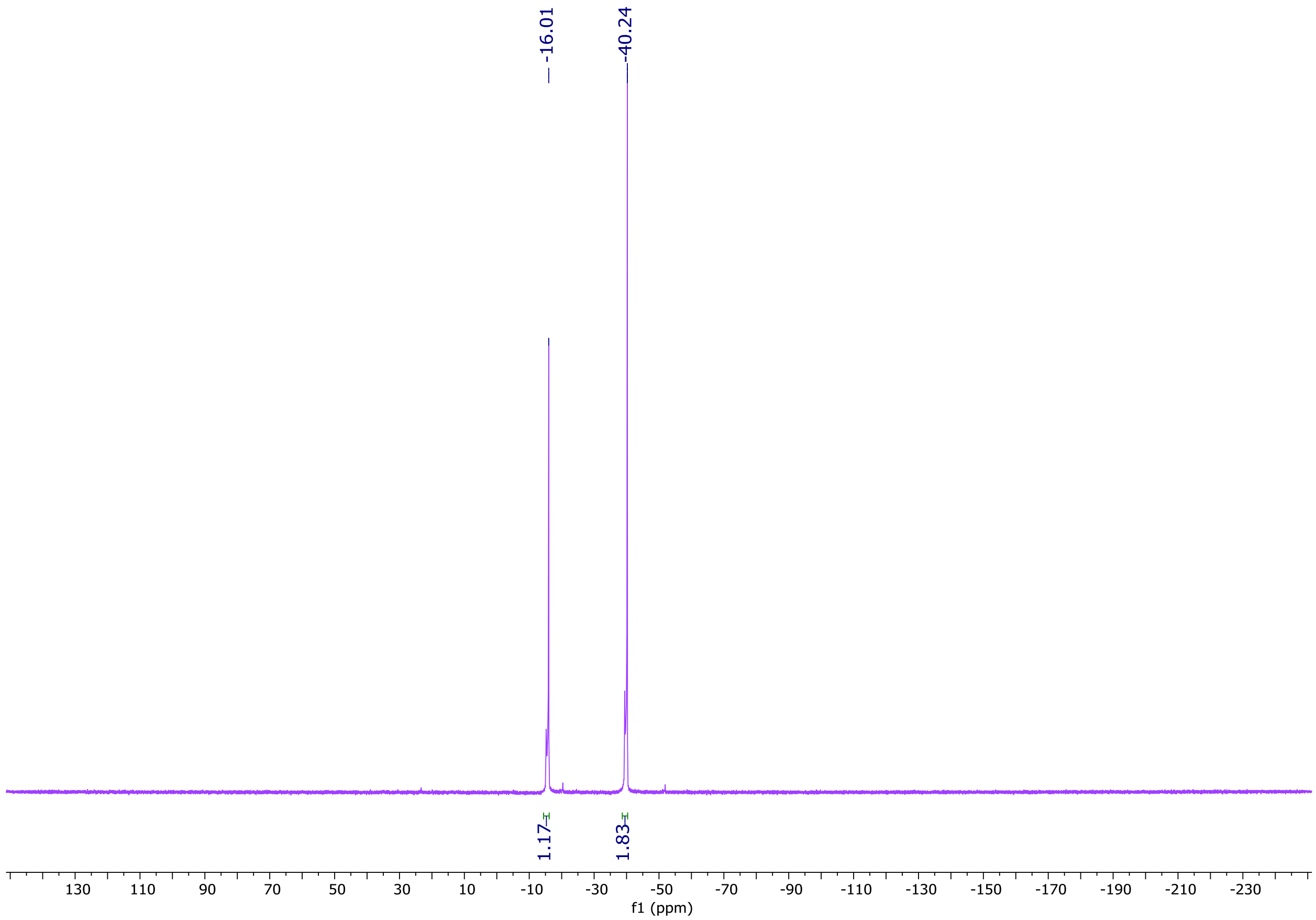

Figure S 55. $^{31}P\{^1H\}$ NMR spectrum (202 MHz, EtOH) of **4** (p-fluorostyrene and Ph_2PH).

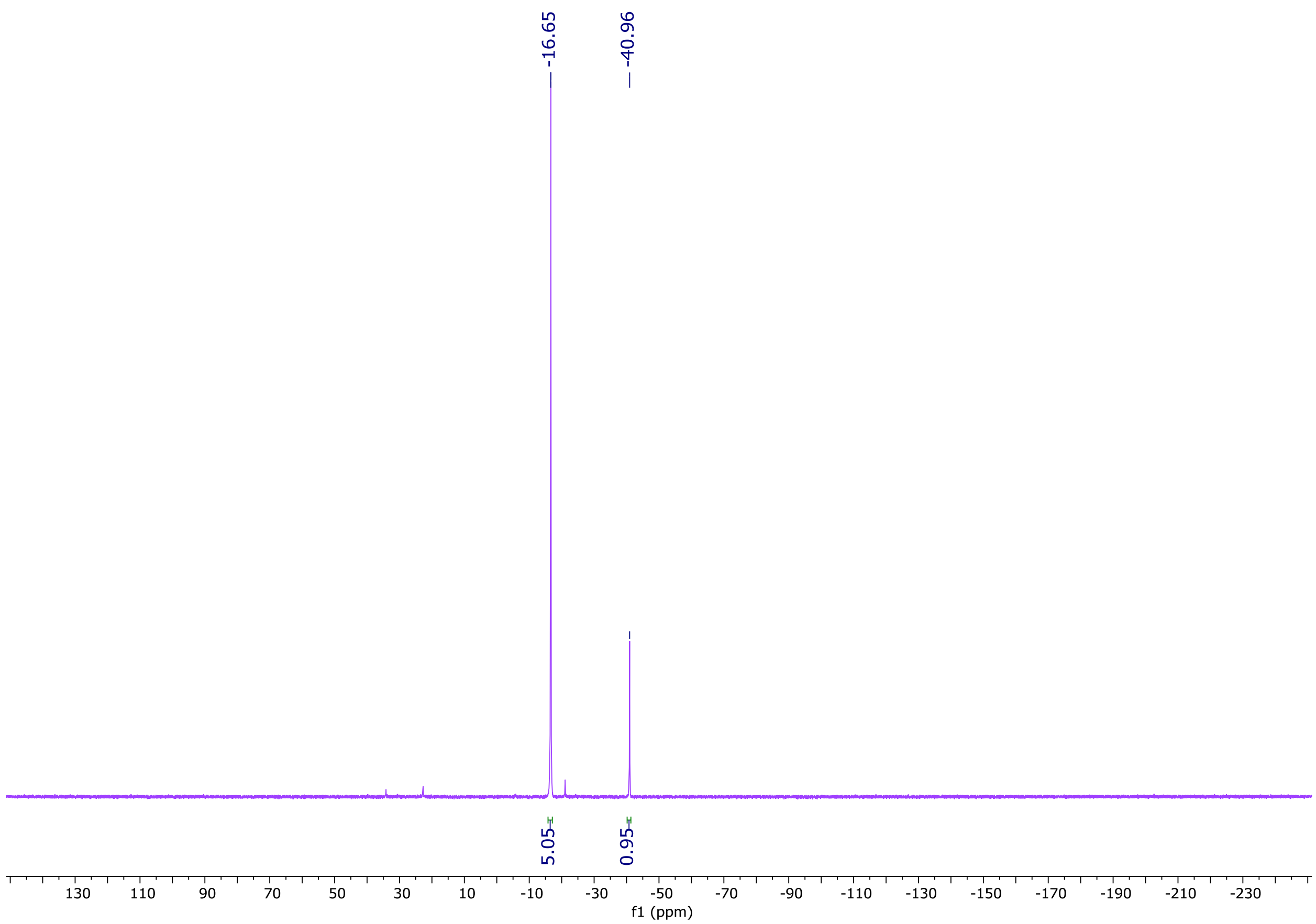

Figure S 56. $^{31}P\{^{1}H\}$ NMR spectrum (202 MHz, EtOH) of **5** (p-bromostyrene and Ph_2PH).

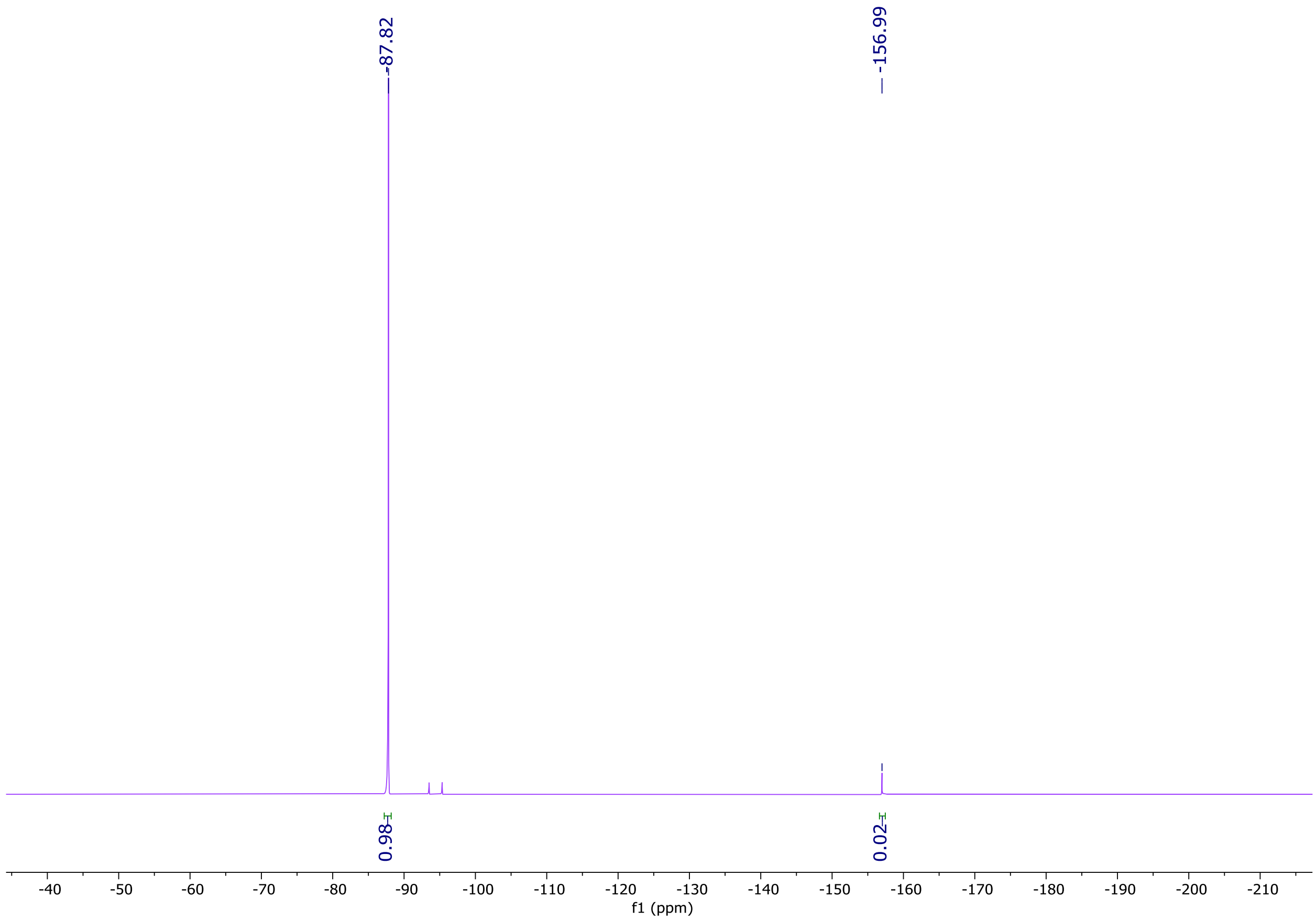

Figure S 57. $^{31}P\{^{1}H\}$ NMR spectrum (202 MHz, EtOH) of **8** (styrene and $MesPH_2$).

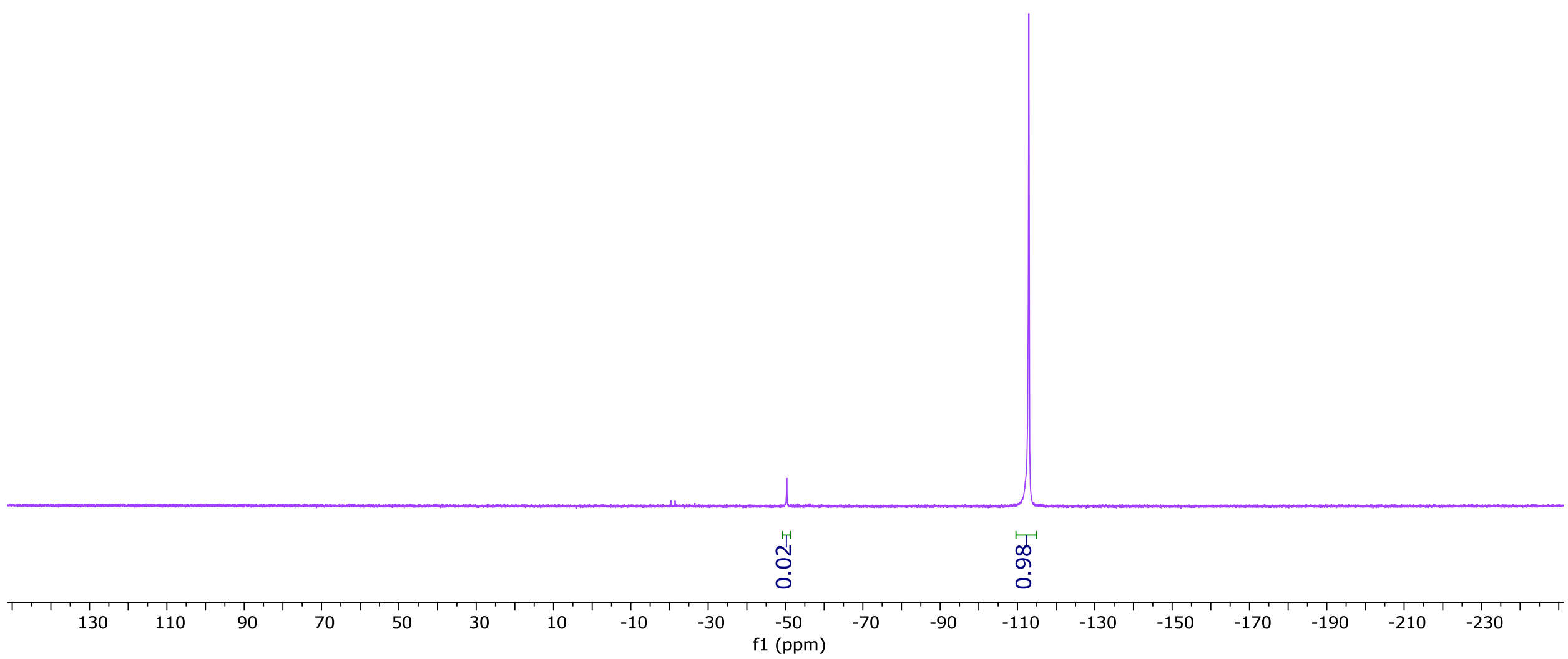

Figure S 58. $^{31}P\{^1H\}$ NMR spectrum (202 MHz, EtOH) of **10** (styrene and $CyPH_2$).

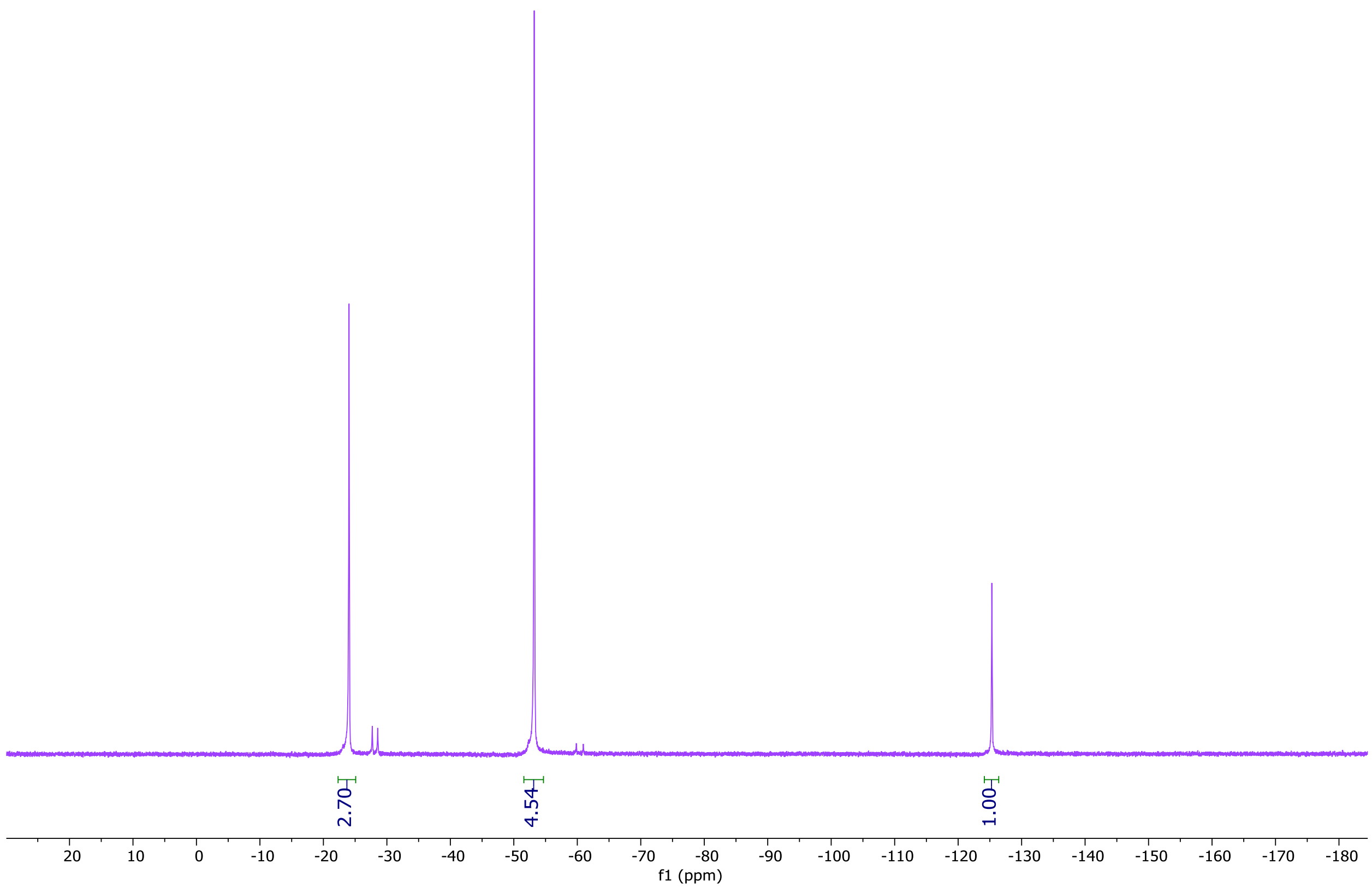

Figure S 59. $^{31}P\{^1H\}$ NMR spectrum (202 MHz, EtOH) of **6** and **7** (styrene and 0.5 $PhPH_2$).

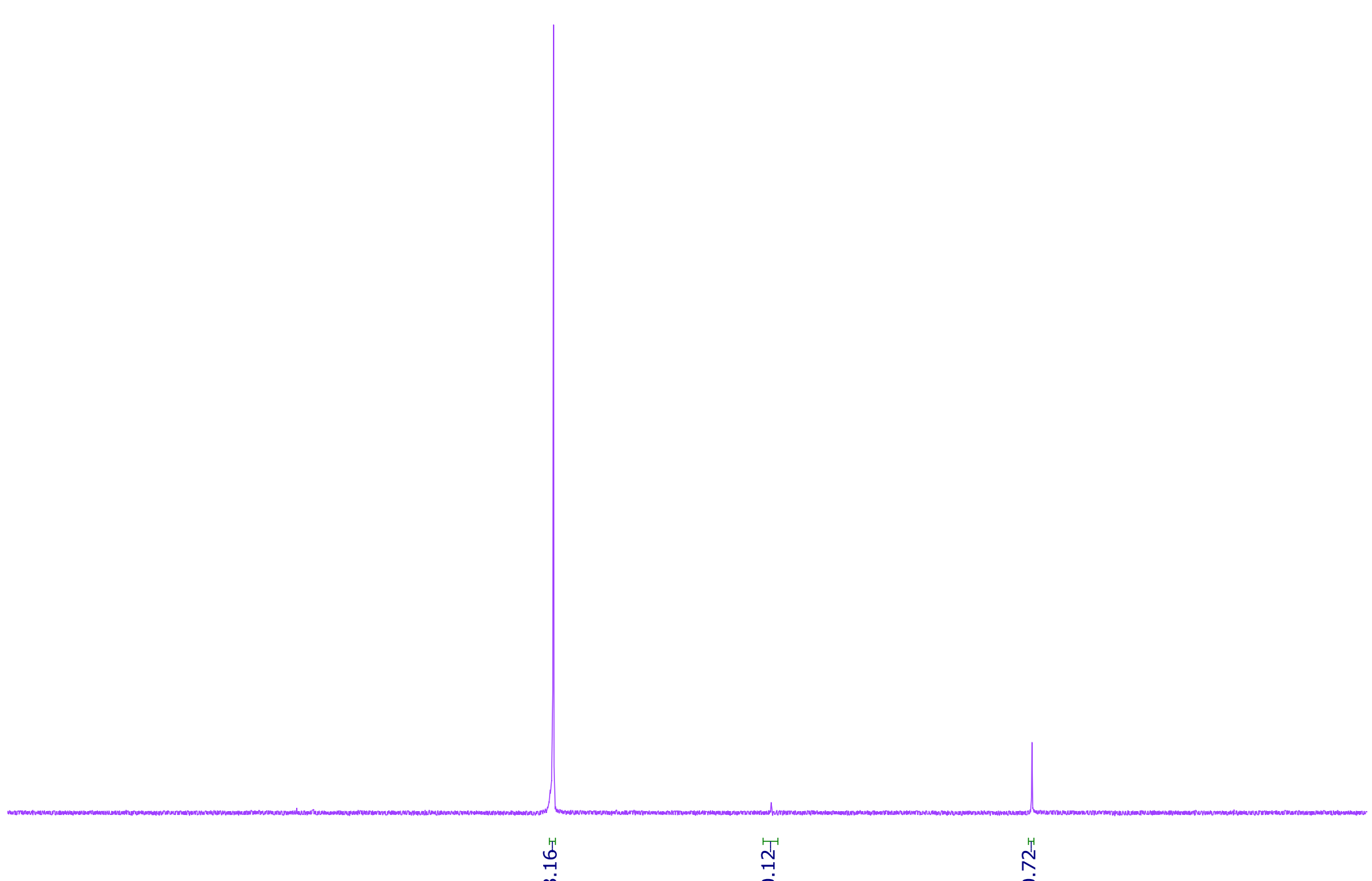

Figure S 60. $^{31}P\{^1H\}$ NMR spectrum (202 MHz, EtOH) of **6** and **7** (styrene and 1 $PhPH_2$).

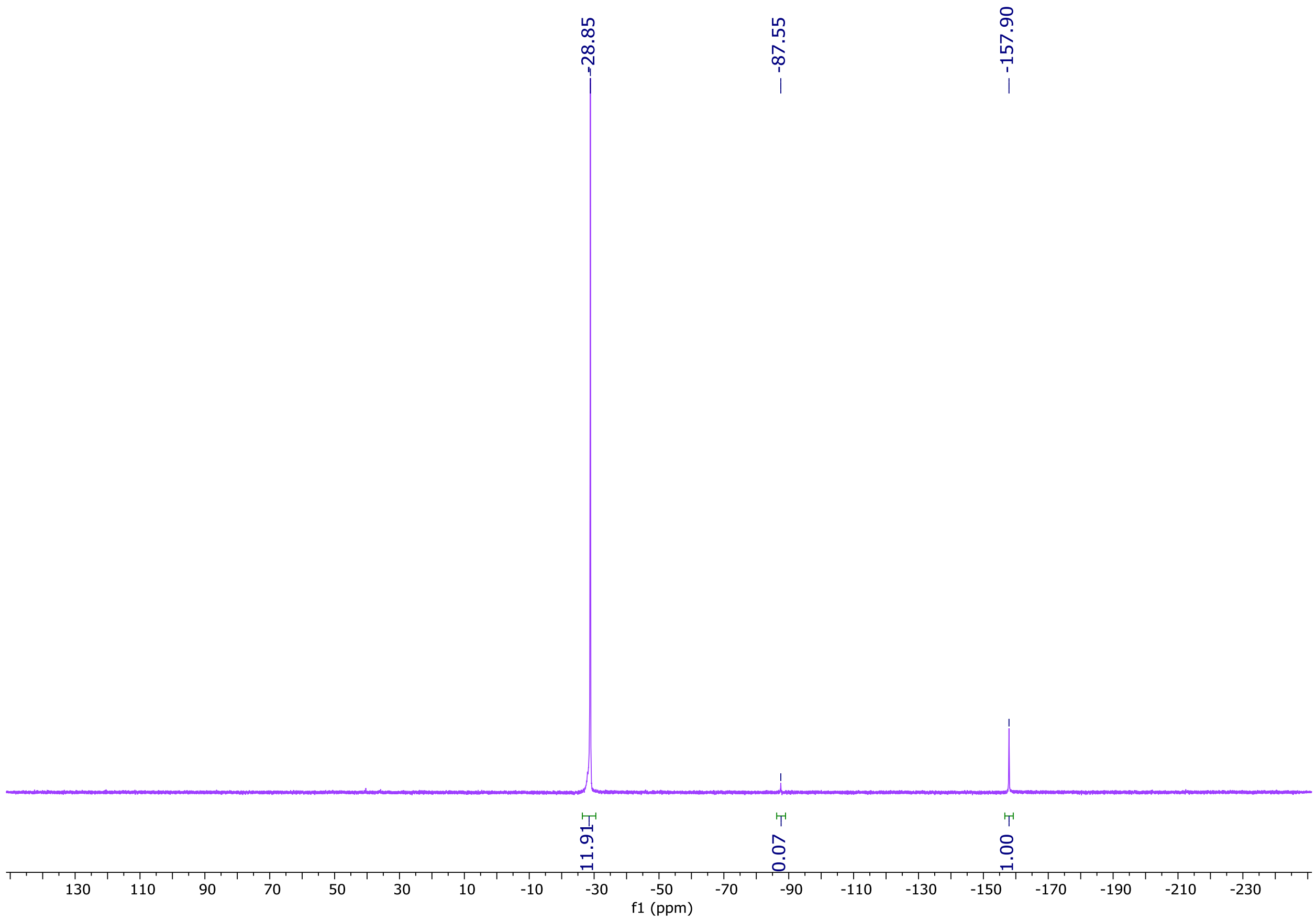

Figure S 61. $^{31}P\{^{1}H\}$ NMR spectrum (202 MHz, EtOH) of **17** (ethyl acrylate and $MesPH_2$).

References

(1) Bedard, J.; Caschera, A.; Foucher, D. A. Access to thermally robust and abrasion resistant antimicrobial plastics: synthesis of UV-curable phosphonium small molecule coatings and extrudable additives. *RSC Adv.* **2021**, *11* (10), 5548-5555, 10.1039/D1RA00555C. DOI: 10.1039/D1RA00555C.

(2) Dannenberg, S. G.; Seth, D. M., Jr.; Finfer, E. J.; Waterman, R. Divergent Mechanistic Pathways for Copper(I) Hydrophosphination Catalysis: Understanding That Allows for Diastereoselective Hydrophosphination of a Tri-substituted Styrene. *ACS Catal.* **2023**, *13* (1), 550-562. DOI: 10.1021/acscatal.2c05221.

(3) Finfer, E. J.; Waterman, R. Neoteric solvents for exploratory catalysis: hydrophosphination catalysis with CHEM21 solvents. *Green Chem.* **2025**, *27* (2), 432-437, 10.1039/D4GC05160B. DOI: 10.1039/D4GC05160B.
